# An anaerobic pathogen rewires host metabolism to fuel oxidative growth in the inflamed gut

**DOI:** 10.1016/j.cell.2026.04.012

**Published:** 2026-04-30

**Authors:** Luisella Spiga, Ryan T. Fansler, Yifan Wu, Alexandra Grote, Madison Langford-Butler, Asia K. Miller, Maxwell Neal, Owen F. Hale, Deepanshu Singla, M. Wade Calcutt, Abigail E. Rose, Madeline M. Bresson, Alexandra C. Schrimpe-Rutledge, Brittany Berdy, Simona G. Codreanu, Mary Kay Washington, Benjamin P. Bratton, Stacy D. Sherrod, John A. McLean, Karsten Zengler, Cynthia L. Sears, Megan G. Behringer, Andreas Gnirke, Lili Tao, Jonathan Livny, Danyvid Olivares-Villagómez, Ashlee M. Earl, Wenhan Zhu

**Affiliations:** 1 Department of Pathology, Microbiology, and Immunology, Vanderbilt University Medical Center, Nashville, TN, USA; 2 Vanderbilt Institute for Infection, Immunology, and Inflammation, Vanderbilt University Medical Center, Nashville, TN, USA; 3 Infectious Disease and Microbiome Program, Broad Institute of MIT and Harvard, Cambridge, MA, USA; 4 Department of Medicine, Northwestern University Feinberg School of Medicine, Chicago, IL 60611, USA; 5 Department of Pediatrics, University of California, San Diego, La Jolla, CA, USA; 6 Department of Biological Sciences, Vanderbilt University, Nashville, TN, USA; 7 Evolutionary Studies Initiative, Vanderbilt University, Nashville, TN, USA; 8 Mass Spectrometry Research Center, Department of Biochemistry, Vanderbilt University, Nashville, TN, USA; 9 Center for Innovative Technology and Department of Chemistry, Vanderbilt University, Nashville, TN, USA; 10 Department of Pathology, Vanderbilt University Medical Center, Nashville, TN, USA; 11 Department of Pediatrics, University of California, San Diego, La Jolla, CA, USA; 12 Center for Microbiome Innovation, University of California, San Diego, La Jolla, CA, USA; 13 Program in Materials Science and Engineering, University of California, San Diego, La Jolla, CA, USA; 14 Department of Microbiology and Molecular Immunology, Bloomberg School of Public Health, Baltimore, MD, USA; 15 Department of Medicine, Division of Infectious Diseases, Johns Hopkins University School of Medicine, Baltimore, MD, USA; 16 Department of Oncology, Johns Hopkins University School of Medicine, Baltimore, MD, USA; 17 Division of Laboratory Medicine, Department of Pathology, Microbiology and Immunology, Vanderbilt University Medical Center, Nashville, TN, USA; 18 Department of Infectious Diseases, Vanderbilt University Medical Center, Nashville, TN, USA; 19 These authors contributed equally; 20 Lead contact

## Abstract

To colonize their host and cause disease, enteric pathogens must deploy their virulence factors to establish distinct nutrient niches. How anaerobic pathogens construct nutrient niches in the densely populated large intestine remains poorly understood. Enterotoxigenic *Bacteroides fragilis* (ETBF) is a classically anaerobic bacterium implicated in inflammation-associated diseases, including colitis and colorectal cancer. Here, we show that ETBF uses its virulence factor, *Bacteroides fragilis* toxin (BFT), to generate and adapt to a localized oxidative niche that supports gut colonization. BFT manipulates colonic epithelial signaling and the bile acid recycling pathway, inducing a metabolic shift in the epithelium from oxidative phosphorylation to glycolysis. This shift increases local concentrations of lactate and oxygen, nutrients that support oxidative metabolism in ETBF. These findings reveal an unexpected strategy by which a classically anaerobic pathogen leverages host metabolic remodeling to generate and exploit an oxidative niche in the inflamed gut.

## In brief

Enterotoxigenic *Bacteroides fragilis* (ETBF), a classically anaerobic pathogen, uses its toxin, BFT, to remodel host metabolism and generate a localized oxidative niche in the colon. By shifting epithelial cells toward fermentative metabolism, ETBF increases lactate and oxygen availability, allowing this anaerobe to deploy oxidative metabolism to adapt to and thrive in the inflamed gut.

## Graphical Abstract

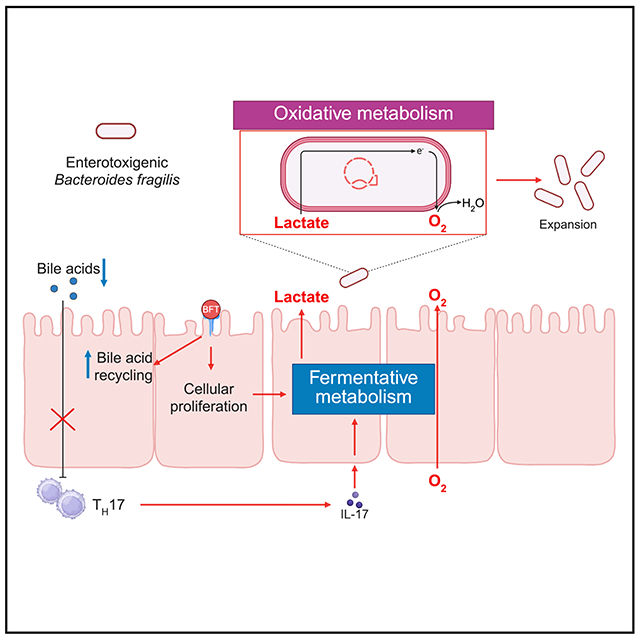

## INTRODUCTION

Enteric infections are a leading cause of acute diarrheal diseases and have recently been implicated in the development of chronic conditions, including colitis and colorectal cancer (CRC).^1,2^ This latter association is particularly important, given that CRC develops at the largest host-microbe interface in the human body^3^ and is predominantly sporadic, with most cases lacking well-defined host germline mutations.^4,5^ Consequently, environmental factors, particularly exposure to enteric pathogens, are increasingly recognized as key contributors to CRC pathogenesis.^2,6–9^ Despite these associations, the mechanisms by which enteric pathogens colonize the gut and influence disease progression remain poorly understood.

Enterotoxigenic *Bacteroides fragilis* (ETBF), a toxin-producing subclass of the common gut commensal *B. fragilis*, has been implicated in both inflammatory diarrheal diseases and CRC across independent human studies.^10–23^ ETBF also promotes chronic inflammatory colitis^24^ and colonic tumorigenesis in gnotobiotic^25^ or conventional^15,26–29^ murine models. These pathogenic effects are primarily driven by the virulence factor *Bacteroides fragilis* toxin (BFT), which elicits a range of physiological alterations in host cells.^26,30–35^ However, the specific mechanisms by which BFT facilitates ETBF niche establishment and promotes persistent colonization in the gut remain largely undefined.

Colonocyte metabolism is central to host-microbe interactions, as it shapes the composition and functions of the gut microbiome.^36^ In the healthy gut, colonocytes generate energy through β-oxidation of microbiota-derived short-chain fatty acids, such as butyrate.^37,38^ This process consumes oxygen from the underlying mucosal surface, effectively maintaining an anaerobic environment in the colonic lumen.^39^ As a result, obligate anaerobic bacteria, which rely on anaerobic fermentation to generate energy, dominate the gut microbiota.^40^ To successfully colonize the densely populated intestine, pathogens must employ virulence factors to manipulate host responses, carving out new nutrient niches.^41^ For instance, facultative anaerobes such as *Salmonella enterica serovar* Typhimurium and *Citrobacter rodentium* subvert colonocyte metabolism to increase luminal oxygen availability.^42–44^ This strategy enables them to engage in respiratory metabolism,^42–44^ thereby outcompeting the resident anaerobic bacteria that rely on the energetically less-efficient anaerobic fermentation to grow.^36^

In contrast to these facultative anaerobic pathogens, members of the *Bacteroidetes* phylum, including ETBF (*B. fragilis*), are classically viewed as anaerobes that exhibit limited growth or viability upon oxygen exposure.^45,46^ As such, they are thought to reside in anoxic niches, such as the mammalian colon, and derive energy primarily through anaerobic fermentation of dietary or host-derived glycans.^47,48^ Current dogma holds that these anaerobes, including ETBF, lack α-ketoglutarate dehydrogenase (SucAB), succinyl-coenzyme A (CoA) synthetase (SucCD), and succinate dehydrogenase (Sdh). This results in a bifurcation of the citric acid cycle (TCA),^49^ incomplete oxidation of acetyl-CoA, and, consequently, less efficient energy production than that of a fully oxidative TCA.

How ETBF, a classically anaerobic pathogen, successfully persists in the inflamed, oxygen-enriched gut it induces,^50^ has remained unclear. Here, we demonstrate that ETBF leverages its virulence factor, BFT, to reprogram epithelial cell metabolism, thereby reshaping the gut nutritional landscape. This reprogramming leads to increased levels of lactate and oxygen, which fuel ETBF’s unique oxidative metabolism. Unexpectedly, BFT also facilitates ETBF adaptation to an oxidative niche independently of host cells. These findings refine the prevailing view of *B. fragilis* physiology and uncover a mechanism by which a classically anaerobic pathogen reshapes its environment to establish an oxidative metabolic niche that promotes its expansion in the inflamed gut.

## RESULTS

### ETBF operates an oxidative metabolism that supports oxygen respiration

**Figure 1.**
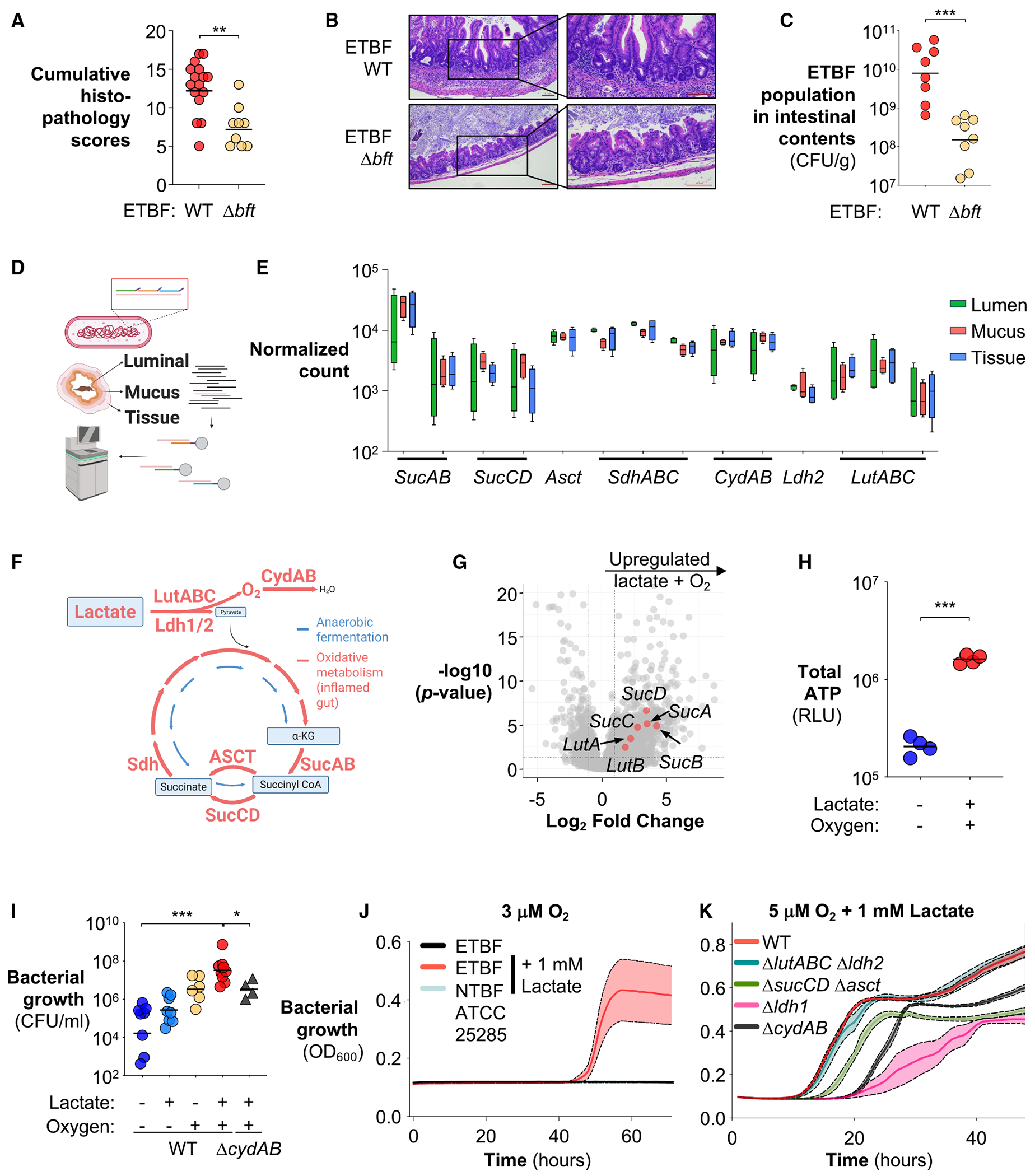
ETBF engages oxidative metabolism during intestinal colonization (A–C) Groups of C57BL/6 mice were colonized with either wild-type ETBF or an isogenic Δ*bft* mutant for 7 days. (A) Histopathology scores of ETBF-infected animals. Histopathology scores were generated using the same scoring criteria across all experiments. (B) Representative H&E-stained images of intestinal tissue. Scale bars, 100 μm and 10 μm (zoomed view). (C) ETBF populations in intestinal contents were quantified by plating on selective media. (D–F) hsRNA-seq was performed on luminal contents, mucus, and cecal tissue to profile the ETBF transcriptome during infection. Transcriptomic data were integrated into a genome-scale metabolic model. (D) Schematic overview of the hsRNA-seq approach. (E) Normalized transcript counts of indicated metabolic genes across sample sites. (F) Predicted metabolic flux through genes involved in lactate oxidation, oxygen respiration, and oxidative central metabolism during ETBF infection, based on transcript abundance (highlighted in red). (G) Wild-type ETBF was subcultured in brain heart infusion (BHI) medium supplemented with 1 mM lactate under microaerobic conditions (1 μM O_2_). Differentially expressed genes relative to control conditions were visualized by volcano plot. (H–K) Indicated ETBF strains were cultured in media supplemented with 1 mM lactate and exposed to the indicated oxygen levels. (H) Total cellular ATP levels. (I–K) *In vitro* growth of ETBF strains under the indicated conditions. In (A), (C), (E), (H), and (I), bars represent the geometric means. In (J) and (K), connected lines and shaded areas represent the mean and SEM. *p < 0.05; **p < 0.01; ***p < 0.001. See also Figures S1–S4 and S13.

Because most nutritional niches in the gut are already occupied by the resident microbiota, enteric pathogens often employ virulence strategies to elicit host responses, facilitating the establishment of novel niches that enable their colonization.^42–44,51–55^ To explore whether BFT, a recognized virulence factor previously shown to induce a series of inflammatory changes, reshapes the intestinal nutritional landscape to facilitate ETBF colonization, we colonized groups of C57BL/6 mice with the ETBF wild-type strain and an isogenic toxin-deficient Δ*bft* mutant and assessed their fitness and host inflammatory status. Indeed, loss of *bft* markedly dampened inflammation and reduced pathogen load compared with the wild-type counter-part (Figures 1A–1C).

Next, we sought to determine how ETBF adapts to the inflammatory niches sculpted by BFT. Because bacteria tailor their transcriptional programs to the available nutrients, the bacterial transcriptome can serve as a proxy for the functional state of ETBF metabolism *in vivo*.^56,57^ However, pathogen mRNA is vastly outnumbered by host transcripts^58^ and those from the diverse commensal bacteria.^59,60^ To overcome this, we employed hybrid-selection RNA sequencing (hsRNA-seq),^61^ using capture probes tiled across all predicted ETBF open reading frames. This approach enables the selective capture of ETBF transcripts from total RNA isolated from host tissues and the gut microbial community without introducing substantial enrichment bias (Figures 1D and S1A). Transcriptomic analysis by RNA-seq without target capture revealed that host tissues mount robust responses to BFT, likely reshaping the gut nutritional landscape (Figures S1B and S1C). In turn, ETBF adapts its transcriptome to the nutritional landscape altered by BFT, with 35 pathways significantly enriched among the differentially expressed genes in the lumen (Figure S1D). Notably, these pathways include “ribosome,” “gene expression,” and “translation,” all of which are energy-demanding processes, suggesting that BFT-induced host responses create a nutrient niche that enhances ETBF fitness in the inflamed gut.

Strikingly, transcriptomic analysis revealed that ETBF encodes and actively transcribes key enzymes required for a complete TCA, including Sdh, SucAB, and SucCD, in the inflamed gut (Figure 1E). ETBF also expresses acetate:succinate CoA-transferase (Asct),^62^ which catalyzes the succinyl-CoA-to-succinate conversion, similar to SucCD, but with the added production of acetyl-CoA (Figure 1F), further supporting ETBF’s capacity for oxidative central metabolism. Because these genes are dispensable for fermentative growth and are generally suppressed under anaerobic conditions,^63,64^ their robust expression *in vivo* may reflect an adaptation to increased oxygen availability during inflammation (Figures 1E and 1F), refining the prevailing view of *B. fragilis* as a classically anaerobic organism. Supporting this, with the notable exceptions of *sdh* and *asct*, transcript levels of oxidative metabolic genes were generally highest in bacteria associated with intestinal tissue, compared with those in mucus or lumen fractions (Figure 1E), likely reflecting the tissue-to-lumen oxygen gradient. Integrating the *in vivo* transcriptome into genome-scale metabolic modeling predicted substantial flux through three lactate dehydrogenases (Ldh1, Ldh2 [RS13195], and LutABC), along with components of oxygen respiration (CydAB) and the oxidative TCA (SucAB, SucCD, and Asct) (Figure 1F). In contrast, the flux through these genes is minimal under anaerobic conditions (Figure 1F). Interestingly, *sucAB* (E1 subunit), *sucCD*, and *ldh2* display mosaic distribution within the *Bacteroides* genus, with higher prevalence in those members with relatively higher oxygen tolerance, suggesting that these genes may participate in adaptation to oxidative niches^65^ (Figure S1E). Together, these findings suggest that ETBF operates a metabolic program that diverges from anaerobic fermentation pathways typically associated with other *Bacteroides*.^66^

### Oxygen and lactate drive an oxidative metabolism in ETBF

Cellular respiration involves the oxidation of an electron donor (e.g., lactate) and the transfer of the resulting electrons through an electron transport chain to an electron acceptor, such as oxygen.^67^ By allowing for more complete oxidation of carbon sources and more efficient energy conservation, respiration generates ATP more efficiently, conferring a metabolic advantage over anaerobic fermentation.^68^ Oxygen is the most favorable electron acceptor, given the high free energy yield; however, most anaerobes exhibit limited growth or viability upon exposure to oxygen or derived reactive oxygen species (ROS), precluding its use for respiration.^45,69^ Notably, recent work revealed that a subset of anaerobes (nanaerobes), including *B. fragilis*, exhibit enhanced oxygen tolerance^70^ and can use nanomolar concentrations of oxygen as an electron acceptor for additional fitness boosts.^65,71,72^ However, the mechanisms that enable oxygen respiration in nanaerobes and their physiological relevance within the mammalian host remain largely unknown.

In facultative anaerobes, the expression levels of the TCA enzymes primarily respond to the presence of oxygen and carbon sources, such as lactate.^63,73,74^ The abundant transcripts of genes involved in lactate utilization suggest that lactate may be a candidate carbon source for ETBF in the inflamed gut (Figure 1E). We thus asked whether oxygen and lactate drive a complete TCA in ETBF. *In vitro* exposure to oxygen and lactate upregulated genes involved in lactate oxidation and oxidative central metabolism (Figure 1G). As an excellent electron acceptor, oxygen enables the utilization of otherwise poorly fermentable carbon sources, such as lactate,^75^ thereby facilitating ATP production.^76^ Consistent with this, oxygen and lactate synergistically increased both ATP production and bacterial fitness of ETBF *in vitro* (Figures 1H and 1I).

To further test this hypothesis, we inoculated ETBF at low density (2.5 × 10^4^ colony-forming unit [CFU]) into an open, microanaerobic atmosphere with 1–10 μM O_2_ (corresponding to ~0.1%–1%) to minimize the impact of collective scavenging^77^ of oxygen that can occur at high bacterial loads and the resulting decrease in oxygen tension due to microbial consumption in sealed vessels.^65^ Remarkably, lactate supported ETBF growth in the presence of up to 10 μM oxygen, an order of magnitude higher concentration than the nanomolar concentrations previously reported for nanaerobes,^65,71,72^ in a rich, semi-defined medium where other carbon sources are available (Figures 1J and S1F). At 5 μM O_2_, ETBF entered the exponential phase earlier than under anaerobic conditions, consistent with enhanced metabolic efficiency (Figure S1F). In contrast, a non-toxigenic *B. fragilis* (NTBF) strain (ATCC 25285) failed to benefit from these nutrients, even at 3 μM O_2_ (Figure 1J). Notably, the key enzymes that support oxidative metabolism in ETBF are largely conserved in the non-toxigenic strain (Figure S1E), suggesting that differences in transcriptional regulation or enzyme function may underlie the distinct metabolic adaptations to oxygenated environments between the two strains.

### BFT licenses oxidative metabolism and niche adaptation

Because ETBF is genetically distinguished from NTBF primarily by the presence of the *Bacteroides fragilis* pathogenicity island encoding BFT,^78,79^ the fitness defects of the ETBF Δ*bft* mutant in the inflamed gut suggested that BFT itself may facilitate adaptation to this altered metabolic niche. To directly test this hypothesis, we compared microanaerobic growth of the ETBF Δ*bft* strain with that of a complemented derivative in which a single copy of *bft* was reintroduced under its native promoter at a neutral chromosomal site. Remarkably, expression of *bft* alone was sufficient to restore microanaerobic growth (Figure S2A). Moreover, heterologous expression of *bft* enabled multiple NTBF strains, including ATCC 25285, TM4000, and an independent clinical isolate BF-25-0092798, to grow under 0.1% O2 (Figures S2B–S2D). Inducible expression of *bft* from a synthetic promoter^80^ phenocopied native *bft* expression, indicating that BFT itself, rather than the surrounding regulatory elements,^81^ is sufficient to confer this phenotype (Figure S2E). Notably, expression of BFT isoforms 2 and 3, but not isoform 1,^82–85^ enabled hypoxic growth of NTBF strains, revealing isoform-specific differences in the capacity to support hypoxic fitness (Figure S2B). Consistent with prior reports,^86^ hypoxic growth capacity varied across *B. fragilis* isolates and did not segregate cleanly by toxigenic status (Figures S2F–S2I), underscoring the importance of isogenic comparisons for isolating the contribution of BFT.

We next asked whether BFT promotes adaptation to the inflamed gut environment *in vivo*. In single-colonization assays, an isogenic NTBF strain expressing *bft* induced inflammatory gene expression and exhibited a marked fitness advantage relative to its parental strain (Figures S3A–S3C), demonstrating that BFT expression alone is sufficient to promote colonization in the inflamed intestine. To test whether this advantage reflects a strain-intrinsic benefit rather than shared access to a remodeled host environment, we performed competitive colonization experiments using isogenic NTBF and NTBF::*bft* strains. Under these conditions, NTBF::*bft* induced modest inflammation and significantly outcompeted the wild-type NTBF strain (Figures S3D–S3F), indicating that BFT expression confers a competitive advantage even when both strains experience identical host and nutrient environments.

We next asked whether this advantage is amplified in the context of ETBF-driven inflammation. In co-colonization experiments, wild-type ETBF strongly outcompeted NTBF and elicited robust inflammation, whereas the isogenic ETBF Δ*bft* mutant exhibited minimal competitive advantage (Figures S3G–S3I), demonstrating that BFT is required for ETBF’s preferential advantage in this setting. Finally, to test whether BFT can function *in trans*, we isolated outer membrane vesicles (OMVs)^87^ from wild-type ETBF or the isogenic Δ*bft* mutant and administered them to mice colonized with either NTBF or ETBF Δ*bft*. BFT^+^ OMVs, but not BFT^−^ OMVs, induced intestinal inflammation, impaired NTBF fitness, and enhanced colonization by ETBF Δ*bft* (Figures S3J–S3L). Together, these data demonstrate that BFT licenses an oxidative metabolic program that enables *B. fragilis* to adapt to and exploit the inflamed gut environment. Through BFT-dependent host remodeling and enhanced tolerance of oxidative conditions, ETBF, despite being classically considered an anaerobe, can couple lactate oxidation to oxygen respiration and gain a selective advantage in the inflamed intestine.

### Oxidative lactate respiration is required for ETBF colonization in the inflamed intestine

After establishing that ETBF engages an oxidative metabolism under microanaerobic conditions, we next tested whether lactate oxidation and oxygen respiration are required for ETBF fitness.^75,88,89^ Indeed, deleting the genes required for oxygen respiration (Δ*cydAB*),^45^ lactate utilization (Δ*ldh1* or Δ*lutABC* Δ*ldh2*; note that repeated attempts to generate the triple mutant were unsuccessful), or oxidative central metabolism (Δ*sucCD* Δ*asct*) in ETBF significantly impaired its ability to benefit from lactate oxidation under microaerobic conditions (Figures 1I and 1K) but had little to no effect on bacterial fitness when ETBF was cultured anaerobically (Figure S4A).

**Figure 2.**
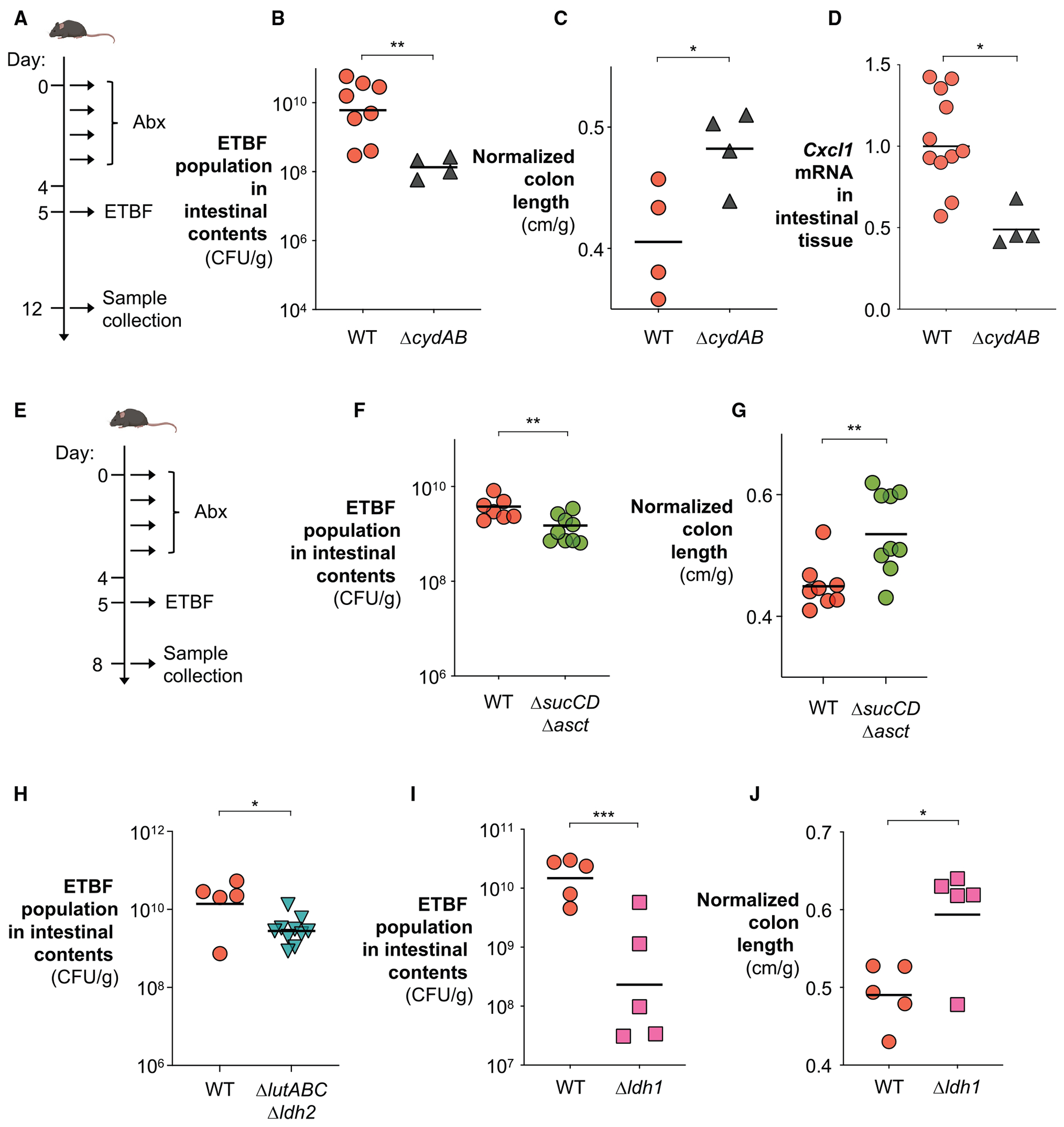
Oxygen respiration and oxidative metabolism contribute to ETBF colonization and inflammation (A–D) Groups of C57BL/6 mice were colonized with the indicated ETBF strains for 7 days. (A) Schematic representation of the experiment. (B) ETBF populations in intestinal contents quantified by plating on selective media. (C) Colon length normalized to body weight. (D) *Cxcl1* transcript levels in intestinal tissue measured by real-time quantitative PCR. (E–J) C57BL/6 mice were colonized with the indicated ETBF strains for 3 days. (E) Schematic representation of the experiment. (F, H, and I) ETBF populations in intestinal contents quantified by plating on selective media. (G and J) Colon length normalized to body weight. Bars represent the geometric means. *p < 0.05; **p < 0.01; ***p < 0.001. See also Figures S4–S6 and S13.

Given that BFT promotes ETBF colonization (Figures 1A–1C and S2), we next asked how ETBF exploits BFT-mediated changes for gut colonization. Because ETBF couples lactate oxidation to oxygen respiration *in vitro*, and actively transcribes genes involved in oxidative metabolism in the inflamed gut (Figure 1), we hypothesize that ETBF relies on oxidative metabolism to exploit the environment created by BFT for efficient gut colonization. Consistent with this notion, ablating the terminal oxidase^45^ (Δ*cydAB*) in the oxygen respiration pathway significantly attenuated ETBF colonization and partially reduced infection-associated intestinal inflammation (Figures 2A–2D). Weighted gene co-expression network analysis (WGCNA) of the intestinal tissue transcriptome revealed that the Δ*cydAB* mutant exhibited reduced infection-associated changes in host processes such as epithelial-mesenchymal transition, reinforcing the notion that ETBF alters host processes to reshape the gut nutritional environment (Figures S4B and S4C).

Engaging an oxidative central metabolism could enable ETBF to couple lactate oxidation with oxygen respiration through an electron transport chain,^75,90^ facilitating its gut colonization. To test this hypothesis, we assessed the *in vivo* fitness of mutants lacking lactate dehydrogenases (Ldh1; LutABC and Ldh2) and key oxidative TCA components (SucCD and Asct; SucAB). In agreement with our *in vitro* observations (Figure 1K), these genes were critical for ETBF fitness and pathogenicity *in vivo* (Figures 2E–2J). In particular, the Δ*ldh1* mutant exhibited an even more pronounced defect in colonizing the intestinal mucus layer, suggesting that lactate utilization in this oxygenated niche is a pathogen-adaptive strategy (Figure S4D). In contrast, deletion of *sucAB* did not result in a notable fitness cost, indicating possible functional redundancy provided by an alternative, uncharacterized enzyme (Figure S4E).

Notably, the reduced colonization observed in the Δ*sucCD* Δ*asct* and Δ*ldh1* mutants was accompanied by attenuated intestinal inflammation (Figures 2G, 2J, and S4F), whereas deletion of the other two lactate dehydrogenases (Δ*lutABC* Δ*ldh2*) or *sucAB* alone had no impact on the inflammatory response compared with the wild-type strain (Figures S4G and S4H). All mutants were generated as non-polar deletions, and reintroduction of a single copy of the deleted gene under its native promoter at a neutral chromosomal locus partially or fully restored fitness both *in vitro* and *in vivo* (Figures S5A–S5H and S6A–S6F). In cases of partial rescue, incomplete restoration of transcript abundance likely reflects differences in regulatory context between the native and complemented loci^91–98^ (Figures S5I–S5K). Importantly, the observed fitness defects were unlikely to result from diminished toxin production, as the metabolic mutants expressed *bft* at levels higher than the wild-type strain early during ETBF colonization (Figures S6G–S6I). By contrast, deletion of the corresponding genes in a NTBF background did not impair *in vivo* colonization (Figures S6J and S6K), indicating that these pathways are selectively required in the BFT-driven inflammatory niche. Together, these findings demonstrate that oxygen respiration, oxidative metabolism, and selective lactate oxidation are key metabolic drivers of ETBF fitness in the inflamed gut, revealing a previously unappreciated capacity of this anaerobe to exploit oxygenated inflammatory niches.

### BFT rewires colonocyte metabolism, increasing oxygen and lactate levels in the gut

**Figure 3.**
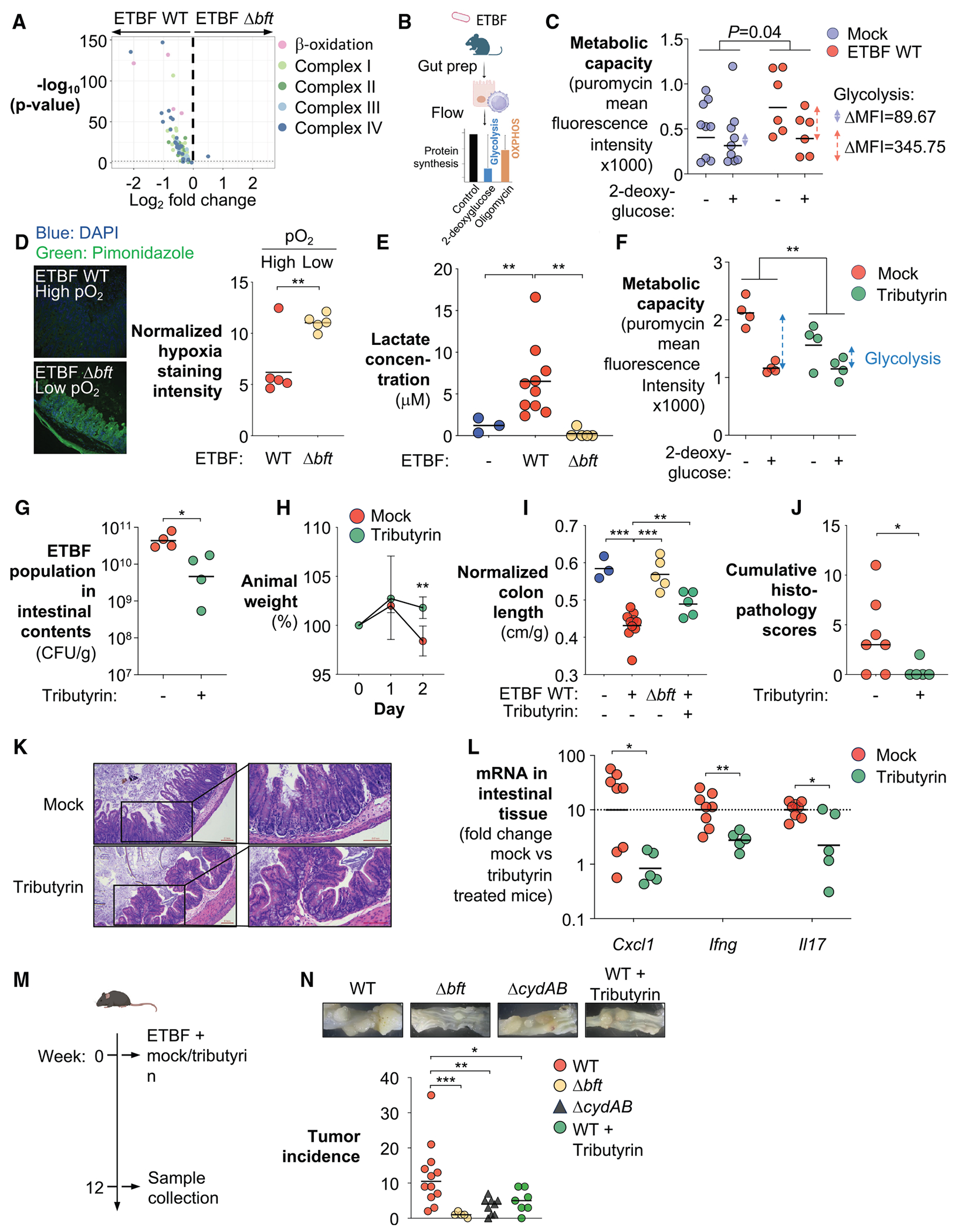
BFT-driven metabolic reprogramming of colonocytes promotes ETBF colonization (A–E) Groups of C57BL/6 mice were colonized with the indicated ETBF strains for 7 days. (A) RNA-seq analysis of intestinal tissues revealed BFT-dependent repression of genes involved in colonocyte oxidative metabolism (β-oxidation, electron transport chain complexes I–IV). (B and C) Metabolic capacity and glycolytic dependency of gut epithelial cells were profiled using the SCENITH assay. (B) Schematic of the SCENITH method. (C) Quantification of metabolic activity and glycolytic dependency in gut epithelial cells. (D) Mice were injected intraperitoneally with pimonidazole 30 min before euthanasia. Hypoxia was assessed using α-pimonidazole antiserum and Alexa 488-conjugated secondary antibody (green fluorescence), with DAPI counterstaining of nuclei (blue fluorescence). Hypoxia staining intensity was normalized to nuclear signal. (E) Lactate concentrations in intestinal contents measured by LC-MS. (F–L) ETBF-colonized C57BL/6 mice were treated with tributyrin or vehicle control for 7 days. (F) Quantification of metabolic activity and glycolytic dependency in gut epithelial cells using the SCENITH assay. (G) ETBF populations in intestinal contents quantified by plating on selective media. (H) Animal weight. (I) Colon length normalized to body weight. (J) Cumulative histopathology scores based on H&E-stained intestinal tissue. (K) Representative H&E-stained images of intestinal tissue. Scale bars, 100 μm and 10 μm (zoomed view). (L) Transcript levels of inflammatory markers were determined by real-time quantitative PCR. (M and N) *Apc*^Min^ mice were colonized with the indicated ETBF strains and received a single dose of tributyrin or a mock control. Samples were collected at 12 weeks post inoculation. (M) Experimental schematic. (N) Total tumor counts in the colon. Bars represent the geometric means. *p < 0.05; **p < 0.01; ***p < 0.001. See also Figures S7, S12, and S13.

Previous studies have shown that BFT elicits a range of host epithelial changes that may converge to reshape the intestinal nutritional landscape. These include the induction of cleavage of zonula adherens junction protein E-cadherin^99^ and hyperplasia through dysregulating Wnt/β-catenin/c-Myc signaling.^24,100^ Crypt hyperplasia, in turn, expands the population of immature colonocytes, which preferentially engage in anaerobic glycolysis rather than the oxidative phosphorylation typical of differentiated colonocytes.^101,102^ Beyond direct effects on epithelial cells, BFT also triggers pathological activation of T-helper 17 cells (T_H_-17),^26,30^ which could further exacerbate the switch to glycolysis through the effector cytokine interleukin 17 (IL-17).^103,104^ Collectively, these effects drive a shift from oxidative metabolism to glycolysis in colonocytes,^36,43,102,105–109^ thereby increasing lactate export and allowing oxygen to diffuse from the lamina propria into the lumen.^89,110^ Consistent with this hypothesis, RNA-seq of colonic tissue from ETBF-infected mice revealed BFT-dependent repression of genes involved in β-oxidation and electron transport chain complexes I–IV (Figure 3A).

To further assess the metabolic state of colonocytes during ETBF infection, we employed the single cell energetic metabolism by profiling translation inhibition (SCENITH) assay to profile metabolic responses in various cell types *ex vivo* via flow cytometry.^111^ SCENITH leverages puromycin incorporation into nascent proteins to measure protein synthesis levels, which serve as a reliable indicator of global metabolic activity.^112^ By using inhibitors of oxidative phosphorylation and glycolysis, we could evaluate the relative contributions of these pathways (Figure 3B). In agreement with previous reports^101,102,110^ and our transcriptomic analysis (Figure 3A), colonocytes primarily operate an oxidative metabolism at homeostasis (Figure 3C). However, upon ETBF infection, colonocytes underwent a notable metabolic shift toward glycolysis (Figure 3C).

A shift from oxidative to glycolytic metabolism in colonocytes reduces oxygen consumption from the underlying vasculature, allowing oxygen to diffuse into the gut lumen.^110,113^ Concomitantly, lactate is secreted as a metabolic end product.^114,115^ Elevated luminal oxygen levels can also impair oxygen-sensitive enzymes in obligate anaerobic commensals, disrupting key metabolic pathways, including central carbon metabolism.^116^ This oxidative stress may force commensals to reroute pyruvate toward lactate production,^117^ further contributing to lactate accumulation in the gut. Indeed, measurement of intestinal oxygenation using pimonidazole, a compound that forms irreversible adducts with proteins or DNA in hypoxic tissues,^118^ revealed that ETBF infection led to increased oxygen tension (Figure 3D). Additionally, lactate quantification revealed a parallel rise in luminal lactate in a BFT-dependent manner, suggesting that the shift to anaerobic glycolysis in colonocytes creates an oxidative, lactate-rich niche (Figure 3E).

If heightened oxygen and lactate foster ETBF growth, restoring epithelial oxidative metabolism should counteract infection. To test this, we therapeutically administered tributyrin, a short-chain fatty acid precursor that promotes oxidative metabolism in colonocytes,^110^ at 2 days post-ETBF inoculation. Feature-level clustering (Figure S7A), WGCNA (Figure S7B), and gene set enrichment analysis (Figure S7C) revealed that tributyrin suppressed glycolytic gene expression. Consistent with these transcriptional changes, tributyrin partially reversed the ETBF-induced metabolic reprogramming of colonocytes toward glycolysis (Figure 3F) and significantly reduced ETBF colonization (Figure 3G). Furthermore, tributyrin treatment ameliorated ETBF-induced weight loss, attenuated intestinal inflammation, and mitigated associated histopathological changes (Figures 3H–3L). Of note, tributyrin did not exhibit direct antibacterial activity against ETBF (Figure S7D) or significantly alter microbiota composition (Figures S7E–S7I), indicating that its protective effect derives from modulation of host metabolism rather than microbial interference. Collectively, these findings support a model in which ETBF exploits elevated luminal oxygen to establish and sustain colonization during gut inflammation.

We next examined whether operating an oxidative metabolism facilitates ETBF-induced tumorigenesis. For this purpose, we utilized the multiple intestinal neoplasia (Min) (heterozygous for the adenomatous polyposis coli [*Apc*] gene) mouse model. Although *Apc*^Min^ mice are considered a small intestine tumorigenesis model, independent studies have demonstrated that ETBF colonization converts *Apc*^Min^ mice into a colon tumor model.^26,119–121^ Notably, ETBF-infected *Apc*^Min^ mice develop colonic tumors within weeks, well before small intestinal adenomas are clinically evident or detectable.^122^ To disrupt ETBF’s respiratory metabolism, we either (1) genetically ablated the terminal oxidase complex (Δ*cydAB*), thereby disrupting ETBF’s ability to respire, or (2) pharmacologically restored epithelial hypoxia by administering a single dose of tributyrin (Figure 3M). 12 weeks post infection, we assessed colonic tumor incidence. Consistent with our hypothesis, both strategies significantly reduced tumor burden (Figure 3N). These findings support a model in which ETBF-induced tumorigenesis is potentiated by its capacity to exploit oxygen and engage in oxidative metabolism, suggesting a link between microbial respiration and cancer promotion in the gut.

### BFT reprograms colonocyte metabolism through interference with epithelial processes

**Figure 4.**
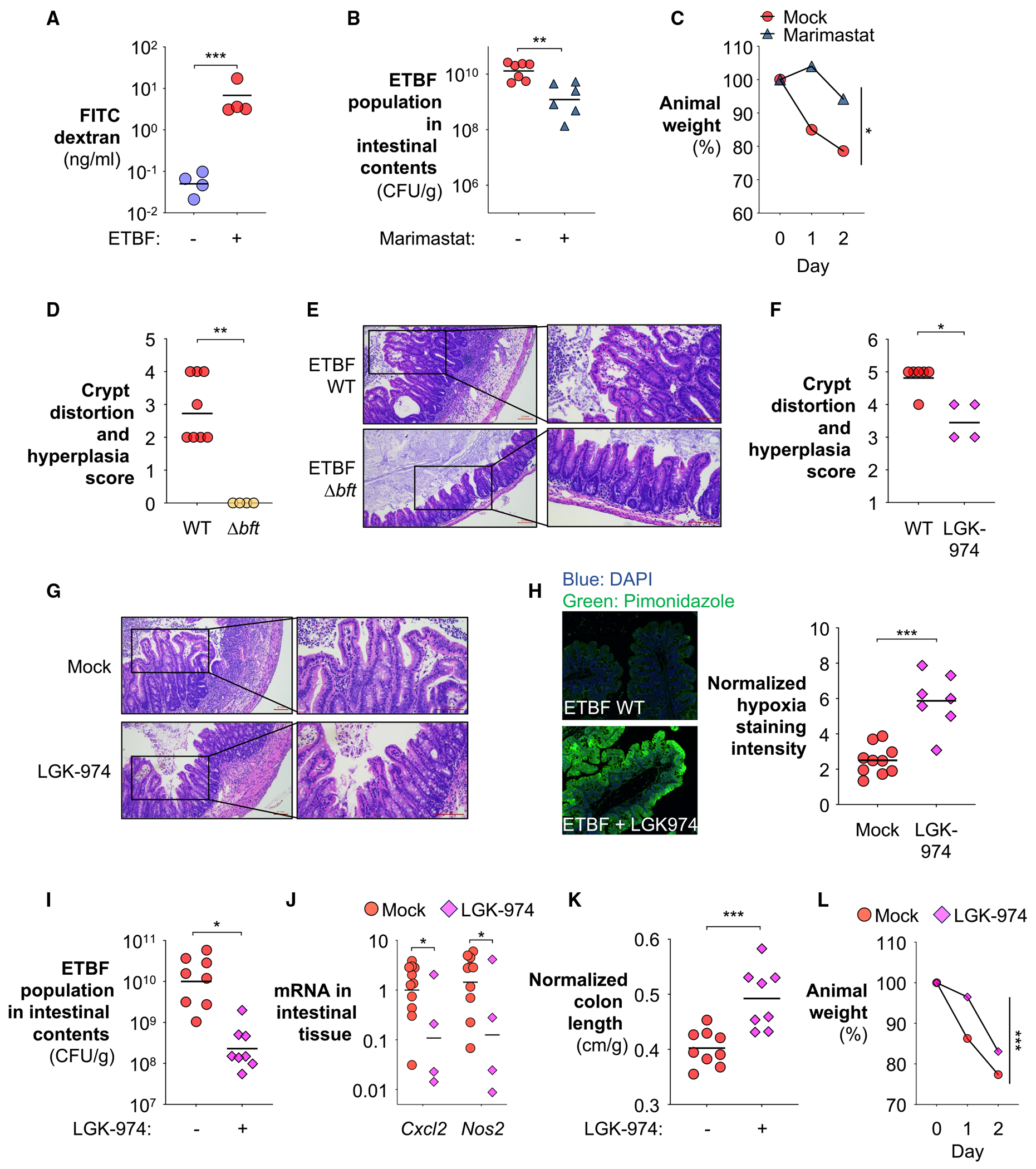
Disruption of epithelial processes promotes ETBF colonization and inflammation (A) C57BL/6 mice were inoculated with either the ETBF wild-type strain or a sham control, followed by intragastric gavage with 4-kDa FITC-dextran. Plasma FITC-dextran levels were measured 4 h post gavage to assess intestinal permeability. (B and C) ETBF-infected C57BL/6 mice were treated with the MMP (matrix metalloproteinase) inhibitor marimastat or vehicle control for 3 days. (B) Animal weights and (C) ETBF burdens in intestinal contents quantified by plating on selective agar. (D and E) C57BL/6 mice were inoculated with either the ETBF wild-type strain or an isogenic Δ*bft* mutant for 7 days. (D) Crypt distortion and hyperplasia scores of the cecum tissue. (E) Representative H&E-stained images of cecal tissue. Scale bars, 100 μm and 10 μm (zoomed view). (F–L) C57BL/6 mice colonized with the ETBF wild-type strain were treated with the Wnt/β-catenin inhibitor LGK-974 or vehicle control for 7 days. (F) Crypt distortion and hyperplasia scores in the cecum. Histopathology scores were generated using the same scoring criteria across all experiments. (G) Representative H&E-stained images of cecal tissue. Scale bars, 100 μm and 10 μm (zoomed view). (H) Epithelial oxygenation measured by normalized hypoxia staining. (I) ETBF burdens in large intestinal contents quantified by plating. (J) Transcript levels of inflammatory cytokines measured by real-time quantitative PCR. (K) Normalized colon length. (L) Animal weight. Bars represent the geometric means. *p < 0.05; **p < 0.01; ***p < 0.001. See also Figures S8, S12, and S13.

A hallmark feature of BFT activity is the induction of proteolytic cleavage of the tight junction protein E-cadherin,^33,99^ compromising colonic barrier integrity. Consistent with this, administration of fluorescein isothiocyanate (FITC)-dextran revealed that ETBF infection markedly increased gut permeability (Figure 4A). Recent work has implicated BFT, a zinc-dependent metalloprotease, in the cleavage of E-cadherin.^123^ However, multiple studies indicate that BFT-induced E-cadherin processing also engages host-dependent proteolytic pathways, in addition to direct toxin activity.^31,33,99,124^ Accordingly, treatment with marimastat,^125^ a broad-spectrum inhibitor of host matrix metalloproteases capable of cleaving E-cadherin,^126^ reduced ETBF colonization and ameliorated animal weight loss (Figures 4B and 4C), supporting a role for host protease-dependent epithelial remodeling downstream of BFT.

E-cadherin proteolysis also leads to the release and nuclear translocation of β-catenin, which activates T cell factor (TCF)-dependent proliferation, driving epithelial crypt hyperplasia.^33,34,127^ Indeed, ETBF infection promoted epithelial hyperplasia in a BFT-dependent manner (Figures 4D and 4E). This excessive division of epithelial cells causes an expansion of undifferentiated, transit-amplifying (TA) cells from their typical location within the crypts to the surface of the epithelium^43^ (Figures S8A and S8B), leading to crypt elongation (Figures 4D and 4E). Unlike mature colonocytes, TA cells primarily undergo anaerobic glycolysis, which does not contribute to hypoxia.^102,128^ Consequently, colonocyte hyperplasia can promote luminal oxygenation and lactate accumulation.

To assess whether crypt hyperplasia modulates the intestinal nutrient landscape, we inhibited Wnt/β-catenin signaling with LGK-974^129,130^ during ETBF infection. LGK-974 attenuated ETBF-induced crypt hyperplasia and its associated histological distortion (Figures 4F and 4G). Consistent with a reversal from glycolysis to oxidative metabolism, LGK-974 partially reverted the BFT-driven shift toward glycolysis in colonocytes and decreased epithelial oxygenation (Figures 4H and S8C). Importantly, partial normalization of colonocyte metabolism via LGK-974 significantly reduced ETBF loads and alleviated inflammation (Figures 4I and 4J), as evidenced by reductions in inflammatory cytokine expression, colon shortening, and weight loss (Figures 4K and 4L). A similar reduction in ETBF colonization was also observed at the peak of infection (day 3, Figure S8D). LGK-974 did not introduce major alterations in overall microbiota composition (Figures S8E–S8I), indicating that its protective effects are primarily host-mediated. Together, these results indicate that toxin-driven epithelial changes establish a pathological oxidative environment that ETBF exploits for robust colonization.

### BFT-dependent bile acid depletion promotes ETBF colonization

**Figure 5.**
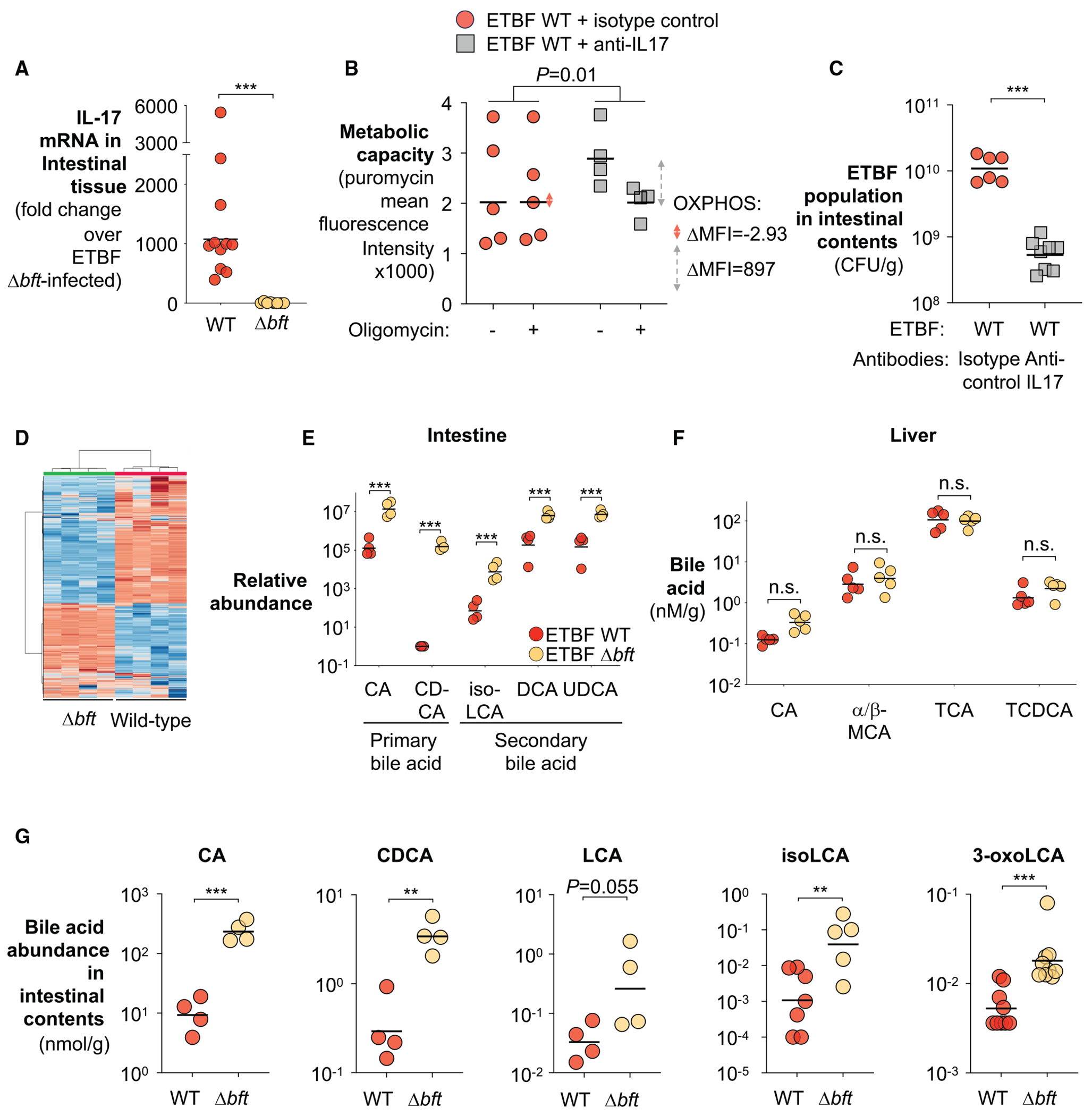
BFT depletes bile acids in the gut (A) C57BL/6 mice were colonized with either the ETBF wild-type strain or the isogenic Δ*bft* mutant for 7 days. *Il17* mRNA levels in intestinal tissue quantified by real-time quantitative PCR. (B and C) Groups of antibiotic-pretreated C57BL/6 mice were colonized with the indicated ETBF strains and treated by intraperitoneal injection with anti-IL-17 neutralizing antibodies or isotype control every 2 days. Intestinal tissues and contents were collected at 3 days post infection. (B) Quantification of epithelial metabolic activity and glycolytic dependency by the SCENITH analysis. (C) ETBF abundance in intestinal contents quantified by selective plating. (D–G) C57BL/6 mice were colonized with either the ETBF wild-type strain or the isogenic Δ*bft* mutant for 7 days. (D and E) Intestinal metabolites analyzed by LC-high-resolution mass spectrometry (HRMS)/MS. (D) Heatmap showing differentially abundant metabolites between groups. (E) Relative abundance of possible bile acid species in intestinal contents. (F and G) Targeted LC-MS/MS quantification of primary bile acids in the (F) liver and (G) intestinal contents. Bars represent the geometric means. n.s., not significant; **p <0.01; ***p < 0.001. See also Figures S9, S12, and S13.

ETBF infection elicits a range of extra-epithelial responses, including a pro-inflammatory cascade that contributes to disease pathogenesis.^30^ One hallmark of this cascade is the induction of a pathological T cell program marked by the production of IL-17 (Figure 5A), a cytokine critical for ETBF pathogenesis.^26^ IL-17 not only amplifies local inflammation but may also exacerbate metabolic reprogramming in epithelial cells, promoting a shift toward glycolysis.^103,104,131^ To test whether IL-17 signaling is functionally required for ETBF-induced epithelial metabolic remodeling, we acutely blocked IL-17 activity *in vivo* using neutralizing anti-IL-17 antibodies with the corresponding isotype controls.^26,132^ Consistent with this hypothesis,^103,104^ acute IL-17 neutralization markedly attenuated ETBF-induced epithelial metabolic remodeling (Figure 5B). Moreover, IL-17 blockade significantly reduced ETBF colonization *in vivo* (Figure 5C). Together, these findings provide direct experimental evidence that IL-17 signaling is a necessary component of a feedforward loop linking inflammation, epithelial metabolic reprogramming, and ETBF persistence. However, the mechanisms by which ETBF triggers IL-17 production and how the response feeds back to support bacterial persistence remain incompletely defined.

To address this gap, we performed untargeted metabolomic profiling on intestinal contents from ETBF-infected mice (Figure 5D). Remarkably, several primary bile acids, cholic acid (CA) and chenodeoxycholic acid (CDCA), steroidal compounds synthesized in the liver and secreted into the small intestine to aid digestion,^133^ were significantly depleted in a BFT-dependent manner (Figure 5E). This loss is unlikely due to reduced biosynthesis, as both hepatic bile acid biosynthesis gene expression and bile acid levels remained unchanged (Figures 5F and S9A–S9D).

Similarly, secondary bile acids derived from microbial transformation of these primary bile acids,^134^ including deoxycholic acid (DCA), isolithocholic acid (isoLCA), and taurourso-deoxycholic acid (UDCA), were also markedly diminished (Figure 5E). Targeted quantification using liquid chromatography-tandem mass spectrometry (LC-MS/MS) confirmed extensive, BFT-dependent depletion of both primary and secondary bile acids (Figure 5G). Notably, a subset of secondary bile acids, such as 3-oxolithocholic acid (3-oxoLCA) and isoLCA, potently suppresses intestinal T_H_17 differentiation and the resulting IL-17 production^105^ through inhibiting the transcription factor retinoic acid receptor-related orphan receptor γt (RORγt).^105,135,136^ This effect likely extends beyond T_H_17 cells, as RORγt also regulates the functions of other IL-17-expressing immune cells, including intraepithelial lymphocytes (IELs), innate lymphocytes, and γδ T cells.^137,138^ As such, the depletion of these secondary bile acids could broadly promote IL-17 signaling through the activation of RORγt-expressing immune cells.

**Figure 6.**
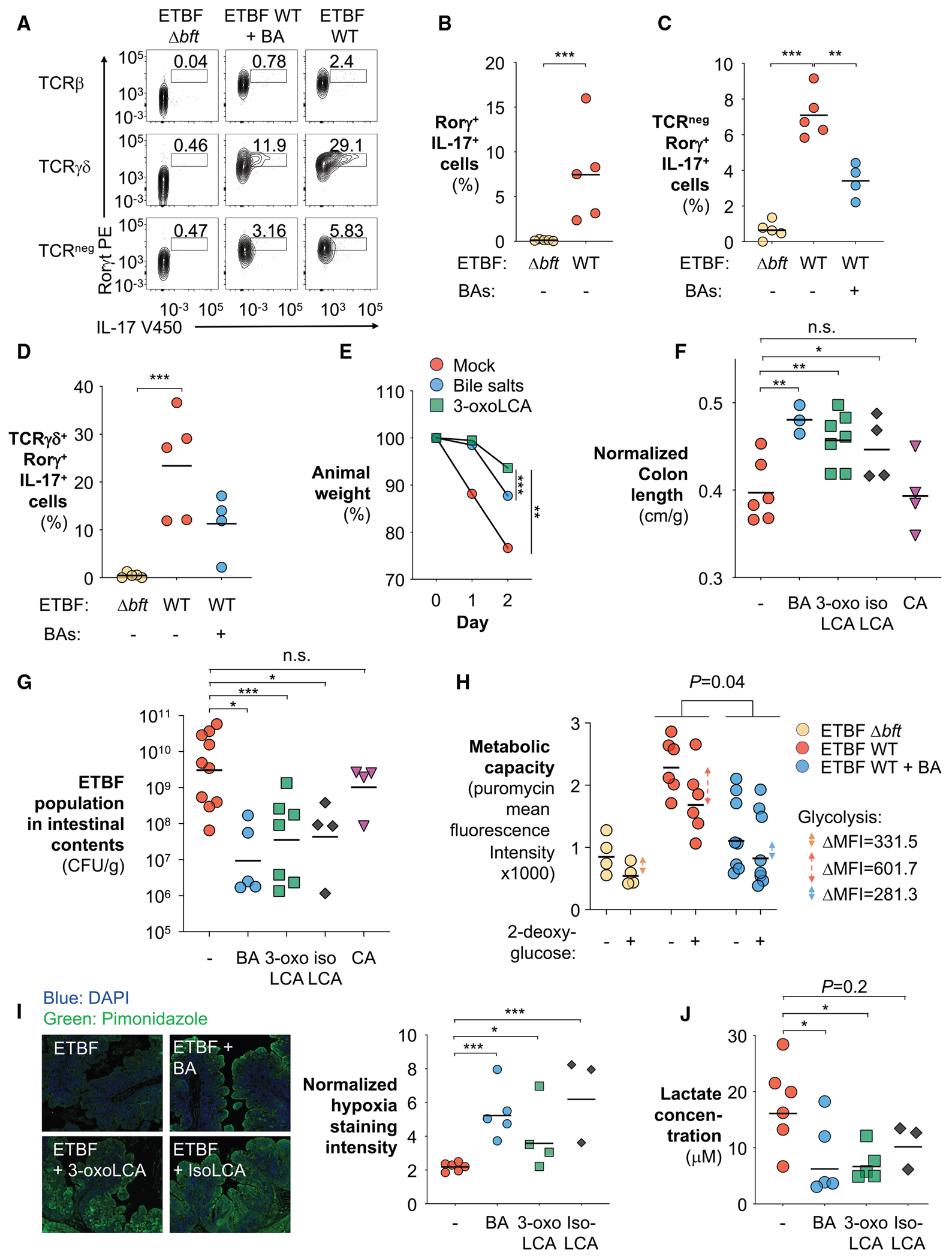
ETBF rewires colonocyte metabolism by impacting bile acid abundance (A–D) Groups of C57BL/6 mice were colonized with wild-type ETBF or the isogenic Δ*bft* mutant and given either vehicle control or primary bile salts in drinking water for 7 days. (A) Flow cytometry analysis of IL-17-expressing intestinal lymphocytes. (B–D) Quantification of IL-17-producing (B) RORγt^+^ immune cells, (C) non-T cell IELs, and (D) γδ T cells. (E–J) Groups of ETBF-colonized C57BL/6 mice were intragastrically administered with the indicated bile acids for 7 days. (E) Animal weight. (F) Colon length normalized to body weight. (G) ETBF abundance in intestinal contents quantified by plating on selective agar. (H) Quantification of metabolic activity and glycolytic dependency in gut epithelial cells using the SCENITH assay. (I) Intestinal oxygenation assessed by hypoxia staining. (J) Targeted LC-MS/MS quantification of intestinal lactate levels. Bars represent the geometric means. n.s., not significant; *p < 0.05; **p < 0.01; ***p < 0.001. See also Figures S10, S12, and S13.

Given the BFT-dependent depletion of 3-oxoLCA and isoLCA observed during ETBF infection (Figure 5G) and the key role of IL-17 in ETBF pathogenesis,^139^ we probed whether bile acid depletion promotes ETBF infection by enhancing IL-17 induction. Profiling IL-17 expression in the ETBF-infected intestine revealed robust BFT-dependent activation of IL-17-producing RORγt^+^ immune cells (Figures 6A and 6B), which include non-T cell IELs (TCR^neg^, RORγt^+^, and IL-17^+^), T_H_17 cells (TCRβ^+^, RORγt^+^, and IL-17^+^), and γδ T cells (TCRγδ^+^, RORγt^+^, and IL-17^+^) (Figures 6C, 6D, and S10A–S10C), consistent with previous reports.^26,132,140^ Importantly, supplementing primary bile acids in drinking water significantly reduced IL-17 production, particularly in non-T cell IELs and γδ T cells, with the latter being critical for ETBF pathogenesis^132^ (Figures 6A, 6C, 6D, and S10A–S10C). Notably, bile acid supplementation also suppressed IL-17 induction in an ETBF-independent inflammatory model using fungal β-glucan curdlan and dextran sulfate sodium^113,141–146^ (Figures S10D–S10F), indicating that bile acids directly restrain IL-17 responses rather than indirectly affecting them through altered ETBF colonization.

We next investigated whether bile acids suppress ETBF infection. Under physiological conditions, approximately 95% of the bile acids secreted are recycled back to the liver, while ~5% reach the colon and undergo microbial transformation into secondary bile acids.^147^ To mimic this physiological context, ETBF-infected mice were given a mixture of primary bile acids at a physiological dose (5 μmol/day/kg), matching colonic exposure under homeostasis.^147^ Consistent with our hypothesis, supplementation of primary bile acids markedly reduced ETBF-associated animal weight loss, intestinal inflammation, and pathogen colonization (Figures 6E–6G). Furthermore, direct supplementation of secondary bile acids, specifically 3-oxoLCA and isoLCA, robustly attenuated ETBF pathogenesis, underscoring secondary bile acid depletion as a critical virulence strategy of ETBF (Figures 6E–6G and S10G). Feature-level clustering analysis of the cecal transcriptome demonstrated distinct molecular signatures associated with supplementation of primary bile acids, 3-oxoLCA, and isoLCA (Figure S10H). Transcriptome analysis further revealed that primary bile acids promoted enrichment of pathways linked to mitochondrial respiration and oxidative phosphorylation, aligning with our observation of restored epithelial metabolic homeostasis (Figures S10I and S10J). Importantly, supplementation with CA, a primary bile acid that does not yield T_H_17-inhibiting secondary bile acids such as isoLCA and 3-oxoLCA,^136^ did not impact ETBF colonization or disease severity (Figures 6F and 6G). This observation supports the hypothesis that the observed protective effects are specifically mediated by secondary bile acid-dependent immune modulation rather than the general antimicrobial properties of bile acids.^148^

Given that IL-17, the key effector cytokine of the T_H_17 response, has been linked to epithelial adaptation to anaerobic glycolysis,^103,104,131^ we next examined whether ETBF manipulates epithelial metabolism through a bile acid-IL-17 signaling axis by profiling colonocyte metabolic states using the SCENITH assay. Notably, supplementation with primary bile acids significantly suppressed glycolysis in colonocytes (Figure 6H). Furthermore, restoring secondary bile acid levels, either through primary or secondary bile acid supplementation, reduced epithelial oxygenation and luminal lactate accumulation (Figures 6I and 6J). These treatments also attenuated ETBF-induced T cell differentiation, supporting the idea that a pathological T cell response contributes to epithelial metabolic reprogramming (Figure S10K). Together, these findings indicate that ETBF exploits bile acid signaling to reprogram epithelial metabolism, thereby facilitating its colonization of the gut.

### BFT exploits the bile acid recycling pathway to deplete bile acids in the gut

**Figure 7.**
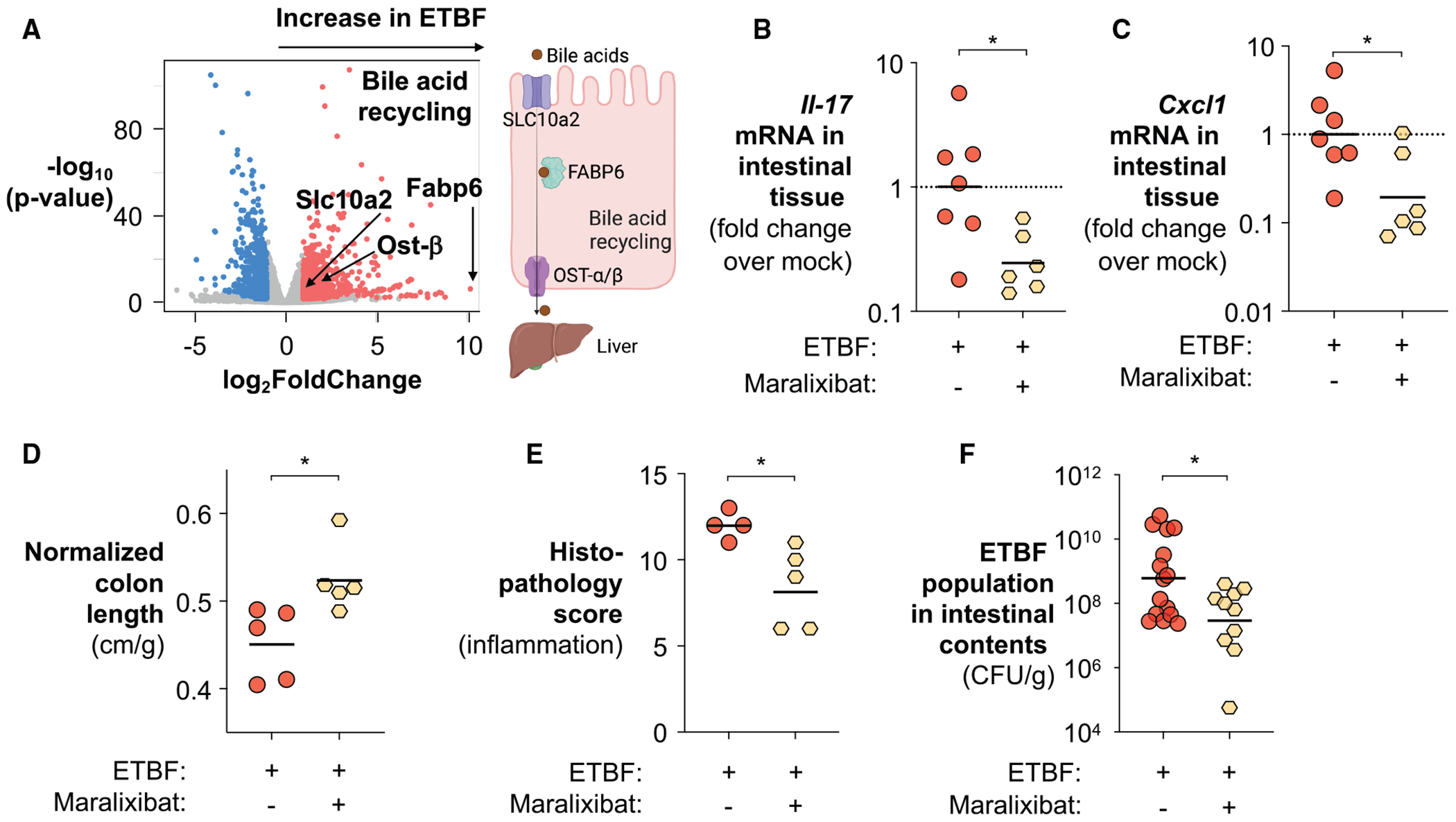
ETBF exploits the bile acid recycling pathway to promote bile acid depletion and gut inflammation (A) C57BL/6 mice were colonized with either wild-type ETBF or the isogenic Δ*bft* mutant for 7 days. The transcriptome of the cecal tissue was profiled by RNA-seq. (B–F) ETBF-colonized C57BL/6 mice received a maralixibat-fortified diet or a control diet for 7 days. (B) *Il17* transcript levels in intestinal tissue measured by real-time quantitative PCR. (C) *Cxcl1* transcript levels measured by real-time quantitative PCR. (D) Colon length normalized to body weight. (E) Cumulative histopathology scores assessing cecal inflammation. (F) ETBF abundance in intestinal contents quantified by plating on selective media. Bars represent the geometric means. *p < 0.05. See also Figures S11, S12, and S13.

We next investigated the mechanism by which BFT promotes bile acid depletion in the intestinal lumen. As BFT did not alter hepatic bile acid levels or the biosynthesis pathway (Figures 5F and S9), this suggests that processes other than bile acid biosynthesis may be implicated in the observed depletion. Given that the majority of secreted bile acids are returned to the liver via the bile acid recycling pathway, we hypothesized that ETBF may exploit this pathway to reshape the intestinal bile acid landscape.^149^ In this recycling pathway, the bile acid transporter SLC10A2 (solute carrier family 10 member 2) mediates uptake of bile acids into enterocytes, after which fatty acid binding protein 6 (FABP6) shuttles them from the luminal side to the basolateral side of the enterocyte for efflux back to the liver via transporters such as organic solute transporter OST-α/β.^150^ Transcriptomic profiling of colonic tissues from ETBF-infected mice revealed that BFT upregulated several key components of the bile acid recycling machinery, including *Slc10a2*, *Fabp6*, and *Ost-α/β* (Figure 7A). With bile acid biosynthesis unchanged, enhanced recycling likely reduces the fraction of bile acids that reaches the colon for microbial conversion into immunomodulatory secondary bile acids. Both SLC10A2 and FABP6 proteins are expressed in the large intestine (Figure S11A), supporting the hypothesis that BFT promotes host-mediated bile acid reuptake to deplete secondary bile acids and amplify IL-17]-driven inflammation.

To further test this, we impeded bile acid recycling using maralixibat, a selective inhibitor of the primary bile acid transporter SLC10A2.^151^ Compared with the vehicle-treated control, maralixibat treatment restored primary bile acid levels in the large intestine of ETBF-infected mice (Figures S11B and S11C). Consistent with our hypothesis, this intervention reduced *Il17* and *Cxcl1* transcript levels, markers of IL-17 signaling and inflammation (Figures 7B and 7C), alleviated colon shortening, and reduced pathogen burden in ETBF-infected animals (Figures 7D–7F). Additionally, cecal transcriptome analysis revealed that maralixibat downregulated genes involved in interferon signaling and γδ T cell activation (Figure S11D), and a broader downregulation of T cell activation pathways was identified by WGCNA (Figure S11E). Importantly, 16S rRNA sequencing of the cecal contents showed an augmentation of *Firmicutes*, known critical members of a homeostatic microbiota, with no significant changes in alpha diversity or beta diversity between treatment groups (Figures S11F–S11J), indicating that the observed effects were unlikely due to shifts in the microbiota. Moreover, none of the host-targeting interventions significantly altered ETBF abundance or *bft* expression at early stages of infection (Figure S12), suggesting that disease attenuation reflects modulation of host responses rather than direct suppression of bacterial growth or toxin expression. Together, these findings support a model in which BFT manipulates host bile acid recycling to suppress the accumulation of immunomodulatory secondary bile acids in the colon, thereby amplifying IL-17-driven inflammation and promoting ETBF colonization (Figure S13).

## DISCUSSION

To thrive in the competitive environment of the gut, many enteric pathogens deploy virulence factors to reshape the intestinal niche, gaining a competitive advantage over commensal microbes. Pioneering research from the Sears laboratory and others identified BFT as the primary virulence factor of ETBF, capable of eliciting profound changes in host physiology. These changes include the cleavage of E-cadherin, induction of Wnt/β-catenin signaling, and activation of transcription factor STAT3 (signal transducer and activator of transcription 3) in gut epithelial cells.^99,100,152^ In immune cells, BFT also induces robust STAT3 activation, promoting IL-17 induction in subsets such as T_H_17 and δγ-T cells.^132^ However, the precise mechanisms by which ETBF benefits from orchestrating these events remain unclear. Here, we show that ETBF reshapes the colonic nutritional landscape by engaging both epithelial and immune cell compartments. BFT drives epithelial hyperplasia and the accumulation of undifferentiated, glycolytic colonocytes. Simultaneously, it activates the bile acid recycling pathway, reducing colonic bile acid levels and derepressing a pathological T cell response. The resulting IL-17 production, in turn, reinforces metabolic reprogramming in colonocytes, creating a feedforward loop that increases oxygen and lactate availability, two metabolites that enhance ETBF colonization (Figure S13). Unexpectedly, BFT also regulates ETBF metabolism via unknown mechanisms, enabling its adaptation to growth under microanaerobic conditions in the absence of host cells.

Our observation that BFT promotes oxygen diffusion into the gut lumen to fuel ETBF colonization has several implications for the pathogenesis of ETBF. First, these observations indicate that ETBF, classically regarded as an anaerobic pathogen, engages an oxidative respiratory metabolism to exploit the altered nutritional landscape for gut colonization. This capability is partly facilitated by enzymes that bridge the two branches of the TCA (e.g., SucCD, Asct, and Sdh). Together with respiratory lactate dehydrogenases and terminal cytochrome bd oxidase (CydAB), ETBF likely runs its electron transport chain more efficiently, generating more ATP and biosynthetic precursors compared with competing gut microbes that rely solely on anaerobic fermentation (Figure 1).

This respiratory capability may explain previous findings that *B. fragilis* can utilize nanomolar oxygen (nanaerobe^65,72^) and appears to be better adapted than other *Bacteroidetes* to thrive within the mucosal environment. Of note, our results suggest that ETBF can grow robustly in the presence of micromolar oxygen, suggesting that this pathogenic strain may have metabolically adapted to the oxidative, inflamed gut. We do not fully understand why ETBF exhibits higher oxygen tolerance than the NTBF. Our data suggest that the bacterial toxin BFT, located on the *B. fragilis* pathogenicity island (BFPAI), likely acquired through horizontal gene transfer, enhances resistance to oxygen via an uncharacterized pathway.^79,153^ Of note, this phenotype is isoform specific, as BFT-2 and BFT-3, but not BFT-1, confer a fitness advantage during hypoxic growth. The molecular basis of this activity remains to be defined.

Beyond supporting oxidative metabolism in ETBF, increased oxygen and ROS, arising from both host inflammatory responses and the intrinsic chemical reactivity of oxygen in anaerobic environments, could damage oxygen-sensitive enzymes in anaerobic commensals.^116^ This metabolic disruption may force these microbes to divert pyruvate toward lactate production.^45,117^ The microbially derived lactate, often in the D-enantiomer form, could contribute to the increase in the total lactate pool. Consistently, ETBF codes for dehydrogenases capable of utilizing both L- and D-lactate (LutABC and Ldh2, and Ldh1, respectively), positioning it to exploit the expanded lactate pool. Moreover, increased oxygen and ROS levels are associated with the depletion of less aerotolerant species that may otherwise compete with ETBF for similar nutrients or deploy a bactericidal type VI secretion system.^51,114,153–155^ Thus, by sculpting an oxidative niche, ETBF both fuels its own growth and suppresses its microbial competitors. Importantly, this distinct metabolic program could potentially be leveraged to selectively target and remove ETBF. For example, we previously exploited the molybdenum cofactor dependency of *Enterobacteriaceae* and used tungstate to selectively inhibit their inflammatory expansion.^156^ Similarly, targeting ETBF’s dependence on metabolically reprogrammed colonocytes could limit its persistence in the gut (Figure 3).

The bile acid-IL-17 signaling axis also offers a potential therapeutic entry point. However, given the broad physiological roles of both primary and secondary bile acids, including regulation of intestinal barrier integrity,^157^ wound healing responses,^158^ immune system functions,^105^ and gut microbiota composition,^134^ strategies to restore bile acid homeostasis need to be approached with caution. These broad impacts of bile acids on intestinal physiology likely contribute to the modest phenotypic impact observed with maralixibat, a selective inhibitor of the bile acid recycling pathway (Figure 7). For example, excessive inhibition of bile acid reabsorption could lead to the accumulation of DCA, a secondary bile acid known to impair colonic epithelial wound healing.^158,159^ This could paradoxically exacerbate ETBF infection by further compromising epithelial integrity and enhancing bacterial access to the lamina propria. These findings underscore the need to explore combination strategies, such as pairing maralixibat with agents that promote epithelial repair, to optimize therapeutic efficacy against ETBF infection while minimizing unintended physiological disruptions.

In summary, this work uncovers a previously underappreciated link between bile acid metabolism, IL-17 response, and colonocyte metabolism. We establish a mechanistic crosstalk between the enteric pathogen ETBF and colonocyte metabolism in the context of colonic inflammation, revealing how toxin-driven metabolic rewiring of colonocytes creates an oxidative niche that supports selective colonization by ETBF in the inflamed gut (Figure S13).

### Limitations of the study

Although ETBF’s capacity for oxidative metabolism improves its fitness during inflammation, it retains the ability to ferment complex glycans.^160^ This metabolic flexibility likely contributes to the significant, but relatively modest, *in vivo* phenotype observed when key components of ETBF’s oxidative metabolism were disrupted (Figure 2). These effects may have been further obscured by a key limitation of our study: the requirement to use antibiotics to permit consistent, robust ETBF colonization.^26,34^ Antibiotic treatment depletes resident microbiota, potentially opening nutrient niches that are normally occupied. This ecological disruption could also liberate alternative nutrient sources, thereby masking the specific contributions of oxygen and lactate—metabolites elevated through BFT-mediated host metabolic reprogramming—to ETBF fitness. Although the current murine model enabled us to dissect the role of oxidative metabolism in ETBF pathogenesis, future development of antibiotic-free colonization models may offer a more physiologically relevant framework to uncover the full spectrum of metabolic strategies that ETBF employs to colonize the inflamed gut. Finally, although we show that BFT unexpectedly enables ETBF growth under microanaerobic conditions in the absence of host cells, the molecular mechanism underlying this bacterium-intrinsic effect remains to be elucidated.

## RESOURCE AVAILABILITY

### Lead contact

Further information and requests for resources and reagents should be directed to and will be fulfilled by the lead contact, Wenhan Zhu (Wenhan.zhu@vumc.org).

### Materials availability

All unique materials generated in this study are available from the lead contact.

## STAR★METHODS

### METHOD DETAILS

#### Bacterial strains, growth conditions, *in vitro* competition assay, and ATP assay

The enterotoxigenic *Bacteroides fragilis* (ETBF) strain 86-5443-2-2 and the isogenic Δ*bft* strain are generous gifts from Dr. Cynthia Sears.^30^ *B. fragilis* strains 1284, YCH46, J38-1, I1345, CL03T12C07, CL05T12C13, and CL07T12C05 are generous gifts from Dr. Laurie Comstock. All clinical *B. fragilis* isolates were obtained in Vanderbilt University Medical Center (VUMC).

ETBF strain 86-5443-2-2 and indicated non-toxigenic *Bacteroides fragilis* (NTBF) strains were grown anaerobically (90% N_2_, 5% CO_2_, 5% H_2_; vinyl anaerobic chamber, Coy Lab) in brain-heart-infusion supplemented (BHIS) media (0.8% brain heart infusion from solid, 0.5% peptic digest of animal tissue, 1.6% pancreatic digest of casein, 0.5% sodium chloride, 0.2% glucose, 0.25% disodium hydrogen phosphate, 0.005% haemin, 0.0001% /vitamin K, pH 7.4) or BHIS plates (BHIS broth, 15 g/L agar) containing 50 μg/mL gentamicin (Gen) for 2 days at 37 °C. When appropriate, agar plates and media were supplemented with 20 μg/mL anhydrotetracycline (aTc), 20 μg/mL cefoxitin (Cfx), 50 μg/mL kanamycin (Kan), 25 μg/mL erythromycin (Erm), 25 μg/mL clindamycin (Clin) or 200 μg/mL 5-fluoro-2′-deoxyuridine (FUdR).

For microaerobic growth, single colonies of the indicated strains were cultured anaerobically for 24 hours in BHIS before subculturing into modified semi-defined media (SDM)^181^ (1.5 g/L KH_2_PO_4_, 0.5 g/L NH_4_SO_4_, 0.9 g/L NaCl, 150 mg/L L-methionine, 5 μg/L vitamin B_12_, 1 mg/L resazurin, 1 g/L tryptone, 0.2% NaHCO_3_, 0.005% protoporphyrin IX) supplemented with 1 mM lactate. Cultures were inoculated at the density of (2.5 x 10^4^ CFU) into an open, microanaerobic atmosphere containing 1-10 μM O_2_ (0.1-1% O_2_, 5% CO_2_, 2.5% H_2_; In vitro Hypoxia system, Coy Lab) and incubated at 37 °C for up to 72 hours.

For *in vitro* competition assays, wild-type and mutant strains of ETBF or NTBF were cultured as described above. Cultures were mixed at a 1:1 ratio and co-inoculated into SDM under microaerobic conditions (0.1-1% O_2_). After 24 hours of incubation, cultures were serially diluted and plated onto selective BHIS agar containing the appropriate antibiotics. Plates were incubated anaerobically at 37 °C for 48 h, after which colony-forming units (CFUs) for each strain were enumerated.

For the ATP assay, single colonies were cultured in SDM anaerobically before subculturing into lactate-supplemented SDM in a microaerobic atmosphere (0.1 % O_2_) for 16 hours. 2.5 x 10^6^−2.5 x 10^7^ cells were used for assaying ATP concentration using an ATP determination kit (BacTiter-Glo Microbial Cell Viability Assay kit, Promega). Sample luminescence was read using white-well plates in a BioTek H1 plate reader and compared to a standard curve of known ATP concentrations.

*E. coli* strains were routinely cultured in LB broth (10 g/L tryptone, 5 g/L yeast extract, 10 g/L sodium chloride) or on LB plates (LB broth, 15 g/L agar) at 37 °C supplemented with 100 μg/mL carbenicillin.

#### Plasmids

All the primers and plasmids used in this study are listed in the key resources table. Suicide plasmids were routinely propagated in *E. coli* S17 λ*pir*. The flanking regions of ETBF strain 086-54443-2-2 *cydAB, lutABC, ldh1, ldh2, asct, sucAB, sucCD* were amplified and assembled into pETBF-Exchange-*tdk* (pEET) using the Gibson Assembly Cloning Kit (New England Biolab, Boston) to give rise to pRF237, pRF231, pML102, pML108, pML191, pRF317, and pRF247, respectively.

The open reading frame BFT-2 together with the 700 bp native promoter region from *B. fragilis* strain 86-5443-2-2 were amplified and assembled into the integrative vectors pNBU2-Cm^R^ or pNBU2-Cfx^R^ using Gibson Assembly (New England Biolab, Boston), generating plasmids pYW283 and pYW279, respectively.

Similarly, BFT-1 together with the native 700 bp promoter region from *B. fragilis* strain J38-1 were amplified and assembled into pNBU2-Cfx^R^ to generate pYW827. The corresponding promoter-ORF fragment of BFT-3 from *B. fragilis* strain Korea 570 was assembled into pNBU2-Cfx^R^, yielding pYW823.

For inducible expression studies, the integration vector pNBU2_Cm^R^-TetR-P1T_DP-GH023 containing the P1T_DP anhydrotetracycline (aTc)-inducible promoter was routinely propagated in *E. coli* S17 λ*pir*. The ORF of BFT-2 from *B. fragilis* strain 86-5443-2-2 was amplified and assembled by Gibson Assembly into pNBU2_catP-TetR-P1T_DP-GH023, a derivative of pNBU2_*erm-tetR*-P1T_DP-GH023^80^ derivative, generating pYW508.

#### Construction of mutants by allelic exchange

All bacterial mutant strains constructed using the method below are listed in the key resources table. For ETBF mutants, suicide plasmid pEET containing the flanking regions of genes of interest was conjugated using *E. coli* S17-1 λ*pir* as the conjugative donor strain into the ETBF. Exconjugants with suicide plasmid integrated into the recipient chromosome were selected on BHIS plates supplemented with appropriate antibiotics. 5-fluoro-2-deoxy-uridine (FudR, 200 μg/mL in BHIS) plates were used to select for the second crossover event.

#### Phylogenetic analysis of genes involved in oxidative central metabolism

RefSeq genome annotations were downloaded using the NCBI Datasets command line tool^177^ (v16.40.1) on April 8^th^, 2025. Complete assemblies for *Bacteroides fragilis*, *Phocaeicola vulgatus*, *Bacteroides uniformis*, *Bacteroides ovatus*, *Bacteroides thetaiotaomicron*, *Bacteroides caccae*, *Bacteroides salyersiae*, *Bacteroides xylanisolvens*, *Bacteroides cellulosilyticus*, and *Phocaeicola dorei* were downloaded, as well as scaffold assemblies for *Hoylesella oralis*, *Bacteroides acidifaciens*, *and Bacteroides nordii*, which were used due to a lack of complete assemblies. Only the latest assembly version was used, and metagenome assembled genomes, assemblies from large, multi-isolate projects, and atypical assemblies were excluded. Annotated protein sequences were used as input for Orthofinder^178^ (v3.0.1b1), which produced the phylogeny and the presence/absence calls for each orthogroup. A phylogeny for the species was using ggtree^175^ (v3.16.0).

#### Animal Experiments

All experiments were conducted in accordance with the policies of the Institutional Animal Care and Use Committee at Vanderbilt University Medical Center. C57BL/6J wild-type (cat# 000664), were obtained from Jackson Laboratory (Bar Harbor) and housed in sterile cages under specific pathogen-free conditions on a 12-hour light cycle, with *ad libitum* access to food and sterile water at Vanderbilt University Medical Center.

Three-week-old male and female mice were randomly assigned into treatment groups before the experiment. To permit stable *B. fragilis* colonization, mice received streptomycin (5 mg/ml) and clindamycin (0.1 mg/ml) in drinking water for 2 days (starting at Day 0), followed by clindamycin alone for an additional 2 days. On day 4, antibiotics were withdrawn and mice were returned to sterile drinking water.

For single-colonization experiments with enterotoxigenic *B. fragilis* (ETBF), mice were orally inoculated on day 5 with 1 × 10^9^ CFU of the indicated ETBF strain or left uninfected as controls. For competitive colonization experiments, mice were inoculated with an equal mixture of 0.5 × 10^9^ CFU of the indicated ETBF strain and 0.5 × 10^9^ CFU of the corresponding mutant strain.

For non-toxigenic *B. fragilis* (NTBF) colonization models, mice underwent the same antibiotic pretreatment regimen and were inoculated on day 5 with 1 × 10^9^ CFU of the indicated NTBF strain or left uninfected. In competitive colonization experiments, mice received an equal mixture of 0.5 × 10^9^ CFU of the NTBF strain and 0.5 × 10^9^ CFU of the indicated NTBF or ETBF strain, as specified. Animals were euthanized at the indicated time points.

For acute IL-17 neutralization, mice were pretreated with antibiotics as outlined above. On day 4, mice received an intraperitoneal injection of 500 μg per animal of either anti-mouse IL-17A antibody (bioXcell) or an isotype-matched mouse IgG1 control (bioXcell), as previously described.^26,171^

Twenty-four hours later, mice were inoculated intragastrically with 1 × 10^9^ CFU of wild-type ETBF. On day 6, mice received a second intraperitoneal injection of anti-mouse IL-17A antibody or isotype control at the same dose. Animals were euthanized on day 7.

For the tumorigenesis model, three-week-old *Apc*^Min^ male and female mice, together with their littermate controls were randomly assigned to treatment groups before the experiment. Mice received streptomycin (5 mg/ml) and clindamycin (0.1 mg/ml) in drinking water for 2 days, followed by clindamycin alone for an additional 2 days to enable stable *Bacteroides fragilis* colonization. On day 4, antibiotics were switched to sterile drinking water. On day 5 mice were then inoculated with 1 x 10^9^ CFU of the indicated ETBF strains or remained uninfected, followed by a single dose of tributyrin treatment. 12 weeks post infection, animals were euthanized, colonic tissue fixed with 4% paraformaldehyde, and tumor incidence was quantified.

For bacterial outer membrane vesicle (OMV) isolation and *in vivo* administration, single colonies of either wild-type ETBF WT or the isogenic Δ*bft* mutant were cultured anaerobically for 24 h in BHIS. The electron-dense layer fraction was isolated by density gradient centrifugation as previously described^182^ and subsequently subcultured into modified SDM. Cultures were grown anaerobically for an additional 16 h.

Bacterial cultures were centrifuged at 5,000 × g for 15 min at 4 °C, and supernatants were filtered through 0.22 μm polyethersulfone (PES) membranes (Pall Corporation) to remove residual cells and debris. OMVs were pelleted by ultracentrifugation at 150,000 × g for 2 h at 4 °C using a Beckman 45 Ti rotor. OMV preparations were resuspended in PBS and quantified by total protein content using a Bradford assay (Bio-Rad). OMVs were normalized to 40 μg total protein per 100 μL PBS for *in vivo* administration, as described previously.^183^

To deliver BFT *in vivo* via OMV, mice were pretreated with streptomycin (5 mg/mL) and clindamycin (0.1 mg/mL) in drinking water for 2 days, followed by clindamycin alone for an additional 2 days. On day 5, mice were inoculated intragastrically with 1 × 10^9^ CFU of either wild-type NTBF or ETBF Δ*bft*. Beginning on day 6, mice received OMVs by oral gavage twice daily for 4 consecutive days.

To induce ETBF-independent intestinal IL-17 responses, seven-week-old female mice were randomly assigned to treatment groups. Mice received oral gavage of curdlan (Sigma-Aldrich) at 10 mg/mL in vehicle (100 μL per mouse) or vehicle alone (5% glucose; Fisher Chemical) once daily for 14 days prior to induction of colitis. Dextran sulfate sodium (DSS; Thermo Scientific) was then administered at 2.5% (w/v) in drinking water for 5 days, with fresh DSS solution prepared daily.^113,141–146^ Following DSS treatment, mice received 2 mM bile salts (Sigma-Aldrich) in drinking water or sterile water for an additional 5 days.

For all experiments, mice were humanely euthanized at the indicated time points. After euthanasia, liver, cecal, and colonic tissue were collected, flash-frozen, and stored at −80 °C for subsequent mRNA analysis or fixed in 10% formalin for histopathological analysis. For untargeted metabolomics, cecal contents were collected and flash frozen in liquid nitrogen, stored in −80 °C until metabolites were extracted. For culture-dependent quantification of bacterial load, colonic and cecal contents were harvested in sterile PBS, and the load of ETBF was quantified by plating serial-diluted intestinal contents on selective agar.

#### Hybrid Selection-RNAseq

Groups of C57BL/6 were challenged with either ETBF wild-type strain or the isogenic Δ*bft* as described above. After euthanasia, cecal samples was lysed immediately after collection in 500μl buffer (0.2M of NaCl and 20mM of EDTA), 210μl of 20% SDS, and 500μl of phenol, chloroform and isoamyl alcohol mixture using lysing Matrix B. Total RNA was extracted and purified from three sites per mouse (intestinal tissue, mucus layer, and luminal contents) across eight mice (24 biological samples in total) using the RNeasy Mini Kit.

RNA was used to generate Illumina cDNA libraries using a modified version of the RNAtag-seq protocol.^184,185^ Briefly, RNA was fragmented, depleted of genomic DNA, dephosphorylated, and ligated to DNA adapters carrying 5′-AN_8_-3′ barcodes of known sequence with a 5′ phosphate and a 3′ blocking group. To ensure sufficient input materials for hybrid capture, libraries were generated using 2.25 ug of total RNA per sample split across 9 technical replicates processed in parallel. Barcoded RNAs from four biological samples were combined in each pool. Pooled RNA underwent rRNA depletion using the Human RiboCop rRNA Depletion Kit (Lexogen, Cat. No. 144), followed by reverse transcription using a primer targeting the constant region of the barcoded adapter, with 3′ adapter addition via template switching (SMARTScribe, Clontech).^186^ cDNA was enriched by PCR for 14 cycles using primers targeting the constant regions of the adaptors with attached Illumina P5/P7 sequences carrying unique indexes per pool. cDNAs from replicate pools generated in parallel were combined and concentrated and used as input for enrichment. The yield of library pool after 14 PCR cycles ranged from 350 (mucus, ETBF Δ*bft*) to 1,600 ng (lumen, ETBF WT). These amplified indexed libraries containing barcoded samples were subjected to hybrid capture enrichment as described below.

Enrichment of ETBF sequences using a custom capture panel and v1 hybridization and wash reagents (Twist Biosciences) was performed in 6 separate capture reactions, each containing approximately 250 ng pre-capture RNAtag library pool. We followed the manufacturer’s instructions except that we added 5 ug mouse Cot-1 DNA (Thermo Fisher) instead of the human blocker solution to the hybridization and used HiFi HotStart ReadyMix (Roche) for PCR amplification of the catch. To empirically determine the minimum cycle number to generate approximately equal quantities of PCR product despite different amounts of ETBF in the three intestinal sites (highest in lumen, lowest in tissue) and ETBF WT (higher) vs. ETBF Δ*bft* (lower), we performed scaled-down 10 ul PCR reactions from 2 ul capture-bead slurry with 8, 12 or 16 cycles. The number of PCR cycles necessary to generate ~100 fmol of each capture library from 25 ul bead slurry ranged from 5 (ETBF WT in lumen) to 11 (tissue).

Pre-capture RNAtag pools were sequenced on two Illumina NovaSeq S4 lanes to an average depth of 9.4x10^8^ read pairs per sample. Capture libraries were sequenced on one lane to an average depth of 4.8x10^8^ read pairs per sample. The sequencing reads generated are available at the NCBI BioProject Database under accession number PRJNA1269620.

#### Analysis of Hybrid Selection-RNAseq data

Sequencing reads from each sample in a pool were demultiplexed based on their associated barcode sequence using custom scripts (https://github.com/broadinstitute/split_merge_pl). Up to 1 mismatch in the barcode was allowed provided it did not make assignment of the read to a different barcode possible. Barcode sequences were removed from the first read as were terminal G’s from the second read that may have been added by SMARTScribe during template switching.

*B. fragilis*-derived reads were aligned to a the GCF 023702735.1 ASM2370273v1 reference sequence using BWA^187^ and read counts were assigned to RefSeq annotated genes and other genomic features using custom scripts (https://github.com/broadinstitute/BactRNASeqCount). Mouse-derived reads were aligned to *Mus musculus* Ensembl sequence GRCm38r94p6 mm10 using bbmap_37.10 (https://jgi.doe.gov/data-and-tools/bbtools/) and read counts were assigned to annotated transcripts using Salmon_0.8.2.^188^

Differential expression analysis was conducted with DESeq2^174^ and/or edgeR.^189^ Visualization of raw sequencing data and coverage plots in the context of genome sequences and gene annotations was conducted using GenomeView.^190^

#### Targeted quantification of mRNA levels in intestinal tissue and contents

Colonic or cecal tissue was homogenized in a bead beater (Precellys 24 Touch, Bertin Technologies) and RNA was extracted using TRI reagent (Molecular Research Center, Cincinnati). Total RNA from the cecum or colon content was extracted using the RNeasy PowerFecal Pro Kit (QIAGEN) per manufacturer’s instructions. DNA contamination was removed using the Turbo DNA-free Kit (Ambion, USA) per the manufacturer’s recommendations. cDNA was generated by SuperScript VILO cDNA Synthesis Kit (Thermo Fisher, USA). Real-time PCR was performed using PowerUp SYBR Green Master Mix (Applied Biosystem, USA), data were acquired in a CFX Maestro 2.3 (Bio-Rad, USA). Target gene transcription of each sample was normalized to *Gapdh* mRNA levels (host) or *mp2* mRNA levels (ETBF).

#### Western blot analysis of FABP6 and SLC10A2 expression in intestinal tissue

Colonic proteins were extracted using RIPA lysis buffer. For each 5 mg of tissue, 600 μL of ice-cold lysis buffer was added, and samples were homogenized using a bead beater (Precellys 24 Touch, Bertin Technologies). Lysates were agitated for 2 hours at 4 °C and then centrifuged at 16,000 g for 20 minutes at 4 °C. Supernatants were collected, and protein concentrations were determined by measuring absorbance at 280 nm using an Epoch Microplate Spectrophotometer (BioTek Instruments).

Samples were boiled at 95 °C for 1 minute, and 50 μg of total protein per sample was resolved on 4-20% TGX precast gels (Bio-Rad). Proteins were transferred onto polyvinylidene fluoride (PVDF) membranes via wet transfer (Bio-Rad). Membranes were blocked in TBS containing 3% non-fat dry milk and 0.1% Tween-20, then incubated overnight at 4 °C with primary antibodies against FABP6 and SLC10A2. After washing, membranes were incubated with appropriate IRDye-conjugated secondary antibodies for 1 hour at room temperature and scanned using an Odyssey Western Blot Imagers (LICORbio). Relative expression levels were quantified using ImageJ, with uniform adjustment of brightness levels across samples.

#### Colonocyte Isolation

The colon and cecum of ETBF-infected animals were removed and kept on ice in RPMI containing 10% FBS. Luminal contents were flushed out with HBSS + 5% FBS + 2mM EDTA. The colon and cecum were then cut longitudinally and into smaller pieces, placed in HBSS medium containing 5% FBS, 2 mM EDTA and 1.5 mM DTT, and shaken at 37 °C for 50 minutes at 150 rpm. After the incubation, the supernatant and the tissue were separated, with the supernatant filtered and centrifuged for 5 minutes at 1200 rpm at 4 °C. The resulting pellet was resuspended in HBSS containing 5% FBS and 2mM EDTA and kept on ice. The tissue was digested in HBSS (contains Ca^2+^ and Mg^2+^) supplemented with collagenase (1.5 mg/mL) and DNase 1 (1000X). After shaking for 25 minutes at 100 rpm, HBSS containing 5% FBS and 2mM EDTA was added to stop the digestion, the remaining tissue was filtered out using cell strainer, and the digested cells were centrifuged for 5 minutes at 1200 rpm at 4 °C. After centrifugation, the two populations (from supernatant and tissue) of cells were combined and further purified using Percoll gradient centrifugation. Cells at the interface of the 40% and 70% layer were collected, centrifuged for 5 minutes at 1200 rpm at 4 °C, washed using RPMI containing 10% FBS, and finally resuspended in 500 μL of the same media. Cell count and viability were quantified using Countess 3 cell counter (Thermo Fisher Scientific).

#### SCENITH assay

Cells were seeded at 1 x 10^6^ cells/ml of RPMI containing 10% FBS in 96-well plates and incubated in a 37 °C CO_2_ incubator for 1 hour. Wells were treated with DMSO Control, 2-Deoxy-D-Glucose (DG, final concentration 250 mM) or Oligomycin (Oligo, final concentration 1 μM). Puromycin (final concentration 10 μg/ml) was added, and the cells were returned to the incubator for 20 minutes. After puromycin treatment, cells were washed in cold PBS and stained with a 1:100 cocktail mix of anti-CD326-APC, CD45 RED Fluo 710, TCR gamma/delta Fluo 450 antibodies, for 30 minutes at 4°C in PBS, 5% FCS and 2 mM EDTA (FACS wash buffer). After washing with cold PBS, ghost viability dye (BV510) 1:100 in FACS buffer was added, and cells were incubated at 4 °C for 20 minutes in the dark. After incubation, cells were washed in FACS buffer and fixed and permeabilized using FOXP3 fixation and permeabilization buffer (Thermofisher eBioscience) following manufacturer’s instructions. Intracellular staining of puromycin was performed by incubating cells for 1 hour at RT with anti-Puromycin antibody (PE) 1:100 in permeabilization buffer. After washing, cells were resuspended in 250 ml FACS buffer (2% FBS + 2mM EDTA in PBS) and transferred in FACS tubes and data were acquired using Fortessa (BD Biosciences). Flow cytometry data were analyzed using FlowJo (BD Biosciences).

#### IL-17-producing immune cell profiling

100,000 to 500,000 cells per well were plated 200 μl/well of stimulation buffer (RPMI containing 10 % FBS, 0.67 μl/ml GolgiStop protein transporter inhibitor, 500 μg/ml ionomycin, 25 μg/ml phorbol 12-myristate 13-acetate (PMA)) in 96-well plates, and incubated in a CO_2_ incubator at 37 °C for 4 hours. Cells were stained with 1:100 TCR γ/δ biotinylated antibodies in FACS buffer (2% FBS + 2 mM EDTA in PBS), and incubated 25 minutes at 4°C. After incubation, cells were washed and incubated with a 1:100 cocktail mix of Streptavidin PE610, CD45 PECy7, TCRβ APC Cy7 for 25 minutes at 4°C. Cells were washed with cold PBS, and ghost viability dye (BV510) was added at a 1:100 ratio in FACS buffer. Cells were then incubated at 4 °C for 20 minutes in the dark. After incubation, cells were washed in FACS buffer, fixed and permeabilized using FOXP3 fixation and permeabilization buffer (Thermo Fisher eBioscience). Cells were washed with 150 μl/well of Perm-Wash buffer (Thermo Fisher eBioscience), and incubated in the dark at RT for 40 minutes with an intracellular antibody cocktail in Perm-Wash buffer. After incubation, cells were washed with Perm-Wash buffer, followed by a second wash in FACS buffer. Cells were finally resuspended in 200 μl FACS buffer and transferred to FACS tubes to be acquired in a FACS machine. Data were acquired using Fortessa (BD Biosciences), and flow cytometry data were analyzed using FlowJo (BD Biosciences).

#### Histopathology

Cecal and colonic tissue were fixed in phosphate-buffered formalin for 48 h and embedded in paraffin. Sections were stained with hematoxylin and eosin. Stained sections were blinded and evaluated using the same criteria listed in the Table S1.

#### Hypoxia staining

ETBF-infected mice with indicated treatment were peritoneally injected with 60 mg/kg pimonidazole (Hypoxyprobe) 30 minutes prior to euthanasia. After euthanasia, samples are collected as described above and fixed in 10% formalin. Tissues were embedded, sectioned and mounted on glass slides. Slides of cecal and colonic tissue were deparaffinized and incubated for 10 minutes at 37 °C with 200 ml of proteinase K (New England Biolabs) solution in TE buffer (10 mM Tris, 1 mM EDTA, pH=8.0). The slides were washed in PBS and blocked for 1 hour at 4 °C with 150 ml of M.O.M mouse IgG Blocking reagent (Vector Laboratories). Samples were incubated with 100 μl of α-pimonidazole rabbit antisera (Hypoxyprobe) at a 1:100 ratio overnight at 4 °C. Samples were washed in PBS and incubated with 100 μl of Alexa-488 conjugated goat anti-rabbit secondary antibody (Cell Signaling, 1:150) for 90 minutes at RT. After washing with PBS, slides were counterstained with Hoechst for 5 minutes, followed by additional PBS washes and mounting. Tiled fluorescence images (5 × 5 fields, 15% overlap) were acquired with Nikon ECLIPSE Ti-2 fluorescence microscope with a 20x objective and the Perfect Focus System enabled. Imaging stitching was performed in real time using NIS-Elements (version 6.10.01). Regions of interest (ROI) were selected manually to exclude well-oxygenated lamina propria. The intensity of pimonidazole staining within each ROI was normalized to the Hoechst signal in the same region to yield normalized Hypoxia staining intensity.

#### Quantification of lactate

Colon and cecal contents of ETBF-infected mice were collected in sterile PBS. Contents were weighed, diluted with 1x PBS to a final density of 40 mg/mL, and homogenized by gentle vortexing at 5 °C. Insoluble debris was removed by centrifugation at 3,000 xg for 10 minutes at 5 °C. The supernatants were collected and stored at −20 °C until the day of analysis.

Lactate and a stable isotope-labeled internal standard lactate-^13^C_3_ (Cambridge Isotopes) were derivatized with the reagent dansyl hydrazine and the carboxyl activating agent 1-Ethyl-3-(3-dimethylaminopropyl)carbodiimide (EDC) to their corresponding dansyl hydrazone derivatives in 250 mM aqueous sodium phosphate pH 4 buffer containing 40% (v/v) acetonitrile. Briefly, 20 μL aliquots of thawed extracts were spiked with 5 nmol lactate-*d*_3_, diluted 1:7 with the derivatization buffer described above, and derivatized at room temperature with the addition of dansyl hydrazine (25 μL x 50 mg/mL in acetonitrile) and EDC (25 μL x 150 mg/mL in water). After one hour at room temperature, 25 μL water containing 5% (v/v) TFA was added to quench the reactions. Following centrifugation at 10,000 x *g* for 30 min at 5 °C, quenched reaction mixtures were transferred to 2-mL autosampler vials equipped with low-volume polypropylene inserts and Teflon-lined rubber septa. The sample injection volume was 10 μL. Calibration standards were prepared in 1x PBS and derivatized in the same manner. LC-MS/MS analysis was performed using a Thermo TSQ Quantum triple-stage quadrupole mass spectrometer interfaced to a Waters Acquity UPLC system. The mass spectrometer was operated in positive ion mode, with detection by multiple reaction monitoring (MRM) using the following transitions: Lactate, 338 → 170, CE 27; Lactate-^13^C_3_, 341 → 170, CE 27. The following analyzer and ESI source parameters were used for the detection of analyte and internal standard: Q1 peak width 0.7 Da (FWHM), Q3 scan width 2 Da, spray voltage 4 kV, capillary temperature 300 °C, tube lens 120, N_2_ sheath gas 50; N_2_ auxiliary gas 10; in-source CID 12. Data acquisition and quantitative spectral analysis were done using Thermo Xcalibur version 2.0.7 SP1 and Thermo LCQuan version 2.7, respectively. Calibration curves were constructed by plotting peak areas against analyte concentrations for a series of ten calibration standards, ranging from 0.10 to 1000 total nmol lactate. A weighting factor of 1/C^2^ was applied in the linear least-squares regression analysis to maintain homogeneity of variance across the concentration range. A Waters Acquity BEH C18 reverse phase analytical column (2.1 x 100 mm, 1.7 μm) was used for all chromatographic separations. Mobile phases were made up of 0.2 % HCOOH + 10 mM NH_4_^+^ Formate in (A) water and in (B) acetonitrile/methanol (9:1). Gradient conditions were as follows: 0–1.0 min, B = 5 %; 1–8 min, B = 5–100 %; 8–9.5 min, B = 100 %; 9.5–10 min, B = 100–5 %; 10–15 min, B = 5 %. The flow rate was maintained at 300 μL/min, and the total chromatographic run time was 15 min. A software-controlled divert valve was used to transfer the LC eluent from 0 to 3 min and from 5.5 to 15 min of each chromatographic cycle to waste.

#### Bile acid quantification

Bile acids (BAs) were measured as their Girard P hydrazide derivatives.^191^ Briefly, to 25 μL of homogenate, a mixture of deuterium-labeled bile acid internal standards was added (5 μL x 25 μg/mL of each: cholate-d_4_, deoxycholate-d_4_, glycocholate-d_4_, chenodeoxycholate-d_4_, ursodeoxycholate-d_4_, and lithocholate-d_4_; all from CDN Isotopes). Spiked samples were then extracted with 750 μL of a solution of methanol/methyl tert-butyl ether/chloroform (1.3:1:1 v/v/v). Following centrifugation at 10,000 x g, the supernatants were removed and evaporated under a gentle stream of nitrogen gas. Residues were reconstituted in 100μL pyridine hydrochloride buffer (300 mM, pH 4.5) before adding 25 μL x 50 mg/mL Girard P reagent in acetonitrile/water (1:1) and 25 μL x 150 mg/mL of the carboxyl activating agent 1-Ethyl-3-(3-dimethylaminopropyl)carbodiimide (EDC) in water to convert BAs to their corresponding Girard P hydrazides. After shaking overnight at 4 °C, reactions were quenched with 25 μL x 5 % (v/v) TFA in water. Quenched reactions were centrifuged at 18,000 x g, then transferred to 2 mL autosampler vials equipped with low-volume polypropylene inserts and Teflon-lined rubber septa. The sample injection volume was 10 μL. BA calibration standards were prepared in water and derivatized in the same manner. Liquid chromatography-high resolution mass spectrometry (LC-HRMS) analysis was performed using a Thermo Q Exactive HF hybrid quadrupole/orbitrap high resolution mass spectrometer interfaced to a Vanquish Horizon HPLC system (Thermo Fisher). High resolution mass spectra were acquired in positive ion mode over a precursor ion scan range of m/z 300 to 800 at a resolving power of 60,000 using the following ESI source parameters: spray voltage 4 kV; capillary temperature 300°C; HESI temperature 100 °C; s-lens 95; N2 sheath gas 40; N2 auxiliary gas 10. Extracted ion chromatograms were constructed for each BA based on the following [M+H]+ exact masses and a mass tolerance of +/− 5 ppm: CA, 542.3588; CA-d4, 546.3840; DCA and CDCA, 526.3639; DCA-d4 and CDCA-d4, 530.3890; LCA and iso-LCA, 510.3690; LCA-d4, 514.3941; 3-oxo-LCA, 508.3534. Data acquisition and quantitative spectral analysis were done using Thermo Xcalibur version 4.1.31.9 and Thermo LCQuan version 2.7, respectively. Calibration curves were constructed by plotting peak areas against analyte concentrations for a series of eleven calibration standards, ranging from 0.15 to 1500 total pmol of each BA. A weighting factor of 1/C2 was applied in the linear leastsquares regression analysis to maintain homogeneity of variance across the concentration range. An Acquity HSS C18 reverse phase analytical column (2.1 x 150 mm, 1.7 μm, Waters, Milford, MA) was used for all chromatographic separations. Mobile phases were made up of 0.2 % formic acid + 10 mM ammonium formate in (A) water/methanol/acetonitrile (8:1:1) and in (B) methanol/acetonitrile (4:1). Gradient conditions were as follows: 0–1.0 min, B = 10 %; 1–15 min, B = 10–100 %; 15–17.5 min, B = 100 %; 17.5–18 min, B = 100–10 %; 18–22 min, B = 10 %. The flow rate was maintained at 300 μL/min, and the total chromatographic run time was 22 min. A software-controlled divert valve was used to transfer the LC eluent from 0 to 5 min of each chromatographic cycle to waste.

#### Untargeted metabolomics

Samples were stored at −80°C until analyzed via liquid chromatography-high resolution tandem mass spectrometry (LC-HRMS and LC-HRMS/MS)-based metabolomics in the Vanderbilt Center for Innovative Technology (CIT) using previously described methods.^192–194^ Briefly, frozen mouse intestinal content samples (n=8, 4 biological replicates for each sample group) were lysed in 1 ml ice-cold lysis buffer (1:1:2, v:v:v, acetonitrile: methanol: ammonium bicarbonate 0.1M - pH 8.0) and sonicated individually using a probe tip sonicator at 50% power (10 pulses). Homogenized samples were normalized by weight to an equal amount per sample. Proteins were precipitated from individual samples by adding 800 μL of ice-cold methanol, followed by overnight incubation at −80°C. Precipitated proteins were pelleted by centrifugation (15k rpm, 15 min), and metabolite extracts were dried in vacuo.

Individual extracts were reconstituted in 100 μl of acetonitrile/water (3:97, v:v) with 0.1% formic acid containing isotopically-labeled carnitine-D9, tryptophan-D3, valine-D8, and inosine-4N15, and centrifuged for 5 min at 10,000 rpm to remove insoluble material. A pooled quality control (pooled QC) sample was prepared by pooling equal volumes of individual samples. The pooled QC was used for column conditioning (8 injections prior to sample analysis), retention time alignment and to assess mass spectrometry instrument reproducibility throughout the sample set.

Global, untargeted mass spectrometry analyses were performed on a high-resolution Q-Exactive HF hybrid quadrupole-Orbitrap mass spectrometer (Thermo Fisher Scientific, Bremen, Germany) equipped with a Vanquish UHPLC binary system (Thermo Fisher Scientific, Bremen, Germany). Extracts (5 μL injection volume) were separated on a Hypersil Gold C18, 1.9 μm, 2.1 mm × 100 mm column (Thermo Fisher) held at 40°C. Reversed phase liquid chromatography (RPLC) was performed at 250 μL/min using solvent A (0.1% FA in water) and solvent B (0.1% FA in acetonitrile/water 80:20) with a gradient length of 30 min as previously described.^195,196^ Intact MS analyses were acquired over the mass-to-charge ratio (m/z) range of 70-1,050 in positive ion mode at 120,000 resolution with a scan rate of 3.5 Hz, automatic gain control (AGC) target of 1x10^6^, and maximum ion injection time of 100 ms, and MS/MS (or tandem) spectra were collected at 15,000 resolution, AGC target of 2x10^5^ ions, with a maximum ion injection time of 100 ms.

#### Metabolomics data processing and statistical analysis

Raw mass spectrometry (MS) data were imported, processed, normalized, and evaluated using Progenesis QI software (version 3.0; Non-linear Dynamics, Newcastle, UK). Relative abundances of spiked, heavy-labeled QA/QC standards showed < 13% variability for sample preparation and <3% for instrument variability. All MS and MS/MS runs were aligned to a pooled quality control (QC) reference sample. Detected ions (i.e., unique retention time and m/z pairs), were de-adducted and de-isotoped to produce distinct analytical features. Data was normalized to all compounds, and statistical significance was evaluated using analysis of variance (ANOVA) on the normalized compound abundance values. Both tentative and putative metabolite annotations (Confidence Level 1-3)^197^ were assigned based on accurate mass measurements (mass error < 5 ppm), isotopic pattern similarity, MS/MS fragmentation spectrum, and retention time matching by querying the Human Metabolome Database^198^ and the CIT’s in-house reference library.

Metaboanalyst 5.0 (www.metaboanalyst.ca/) was used to perform high level visualizations including heat maps for compounds with statistical significance (p-value ≤ 0.05).^199^ The untargeted metabolomics data is available at the NIH Common Fund’s National Metabolomics Data Repository (NMDR) website, the Metabolomics Workbench, https://www.metabolomicsworkbench.org, under the assigned study where it has been assigned Study ID ST003919. The data can be accessed directly via its Project DOI: https://doi.org/10.21228/M8PR93.

#### 16S rDNA amplicon sequencing and analysis

Libraries were prepared from DNA isolated from cecal contents using Qiagen PowerSoil Pro Kit and the V3-V4 hypervariable region of 16S rRNA coding sequences was amplified. The library was constructed and sequenced using an Illumina MiSeq system, generating 250 bp paired-end, chimera-removed reads.^200^ The downstream analysis was performed and visualized with QIIME2^179^ version 2024.5. Taxonomic profiling was performed against SILVA database version 132.^180^ The sequencing reads generated during the current study are available at the European Nucleotide Archive repository under accession No. ENA: PRJEB89357 (secondary accession ENA: ERP172384).

#### Bulk RNAseq and analysis

For *in vitro* bacterial RNAseq, ETBF was cultured in BHIS before subculture in lactate-supplemented BHIS anaerobically or in the presence of 0.1% O2 for 3 hours at 37 °C. Bacteria RNAprotect (Qiagen) was added to preserve the transcript. Bacteria were lysed using Lysing Matrix B (MP Bio) and Tri-reagent (Molecular Biology Center), and RNA was purified using the RNeasy mini kit (Qiagen). Paired-end, 150bp library was constructed using an RNAtag-seq strategy^184^ and sequenced on an Illumina NovaSeq (Illumina, CA). Reads were trimmed using BBMap software suite mapped to the ETBF genome (GCF_023702735.1_ASM2370273v1) using Bow-tie2.^184^ Mapped reads were quantified using featureCounts software package^201^ and differential expression analysis was performed using DESeq2 software.^174^ The sequencing reads generated for this analysis are available at the European Nucleotide Archive repository under accession No. ENA: PRJEB89361 (secondary accession ENA: ERP172389).

For mouse tissue RNAseq, RNA was extracted from cecal tissues of ETBF infected mice using Tri-reagent (Molecular Biology Center) and RNeasy mini kit (Qiagen). Tissues were homogenized using Lysing Matrix C (MP Bio) and Tri-reagent (Molecular Biology Center), and RNA was purified using the RNeasy mini kit (Qiagen). The transcriptome was sequenced using the methods described above. Reads were mapped to the mouse genome (mm10, UCSC Genome Browser) using Bowtie2,^184^ and differential expression analysis was performed using DESeq2 software.^174^ Feature-level clustering, gene set enrichment analysis, and Weighted Gene Coexpression Network Analysis were performed on BigOmics Analytics.^172^ The sequencing reads generated for this analysis are available at the European Nucleotide Archive repository under accession No. ENA: PRJEB89362 (secondary accession ENA: ERP172390).

#### Whole genome sequencing

Bacteroides fragilis isolates were cultured in BHIS. Genomic DNA was extracted using the Quick-DNA Fungal/Bacterial Miniprep Kit (Zymo Research) according to the manufacturer’s instructions. Paired-end sequencing libraries (~150 bp reads) were prepared using an Illumina DNA Prep kit (Illumina, CA) and sequenced on an Illumina NovaSeq platform (Illumina, CA). Genome assembly and bacterial species identification were performed using the BV-BRC platform193 (ver. 3.55.17). The sequencing reads generated for this analysis are available at the European Nucleotide Archive repository under accession No. ENA: PRJEB107014 (secondary accession ENA: ERP188052).

#### Genome-Scale Metabolic Modeling

A genome-scale metabolic model for the enterotoxic strain of *B. fragilis* was reconstructed following established protocols^202^ and the COBRA toolbox version 2.13.3 for Matlab.^203^ In particular, an existing manually-curated and data-validated GEM for *B. fragilis* strain 638R was used as a template,^204^ along with several other gram-negative template GEMS from the BiGG database^205^: multiple *E. coli* GEMs,^206,207^ *Shigella boydii*,^207^ *Shigella dysenteriae*,^207^ *Shigella flexneri*,^207^ and *Klebsiella pneumoniae*.^208^ Metabolic genes used in each of these prior reconstructions were compared to genes in this strain of *B. fragilis* using BLAST (E-value < 10^−30^, identity > 50%); any matching genes and their associated reactions were added to a draft model then manually curated for accuracy. The model was curated to grow in defined media known to be suitable to *B. fragilis*, realistic ATP production, and mass balance as was done for the reconstruction of the existing 638R model.^204^

The model was then transformed into context-specific models for the analysis of fluxes in each of the three sample sites (cecum tissue, lumen, and mucus) utilizing StanDep,^209^ an algorithm for deciding which reactions should be active in different conditions based on transcriptomic data. To simulate the complex medium of the human gut, all nutrients were provided to the models in low amounts (up to one millimole per gram dry weight per hour). The reduced models for each site were then optimized for maximal growth using parsimonious flux balance analysis.^210^ This was then repeated for the isogenic ETBF mutant lacking *bft*. The underlying model for the mutant was not altered before StanDep processing as *bft* is not a metabolic gene present in the reconstruction.

### QUANTIFICATION AND STATISTICAL ANALYSIS

Unless noted otherwise, data analysis was performed in GraphPad Prism v10.4.2. Values of bacterial population sizes, competitive indices, fold changes in mRNA levels, and normalized colon length were normally distributed after transformation by the natural logarithm. A two-tailed Student’s *t*-test was used for ln-transformed data. Unless otherwise stated, *, P < 0.05; **, P < 0.01; ***, *P* < 0.001; ns, not statistically significant. In all mouse experiments, *N* refers to the number of animals from which samples were taken. The outlined statistical details of experiments, including the exact value of *N*, definition of center, and dispersion can be found in the figures or figure legends. Shapiro-Wilk test was used to determine the normality of log-transformed data. Sample sizes (i.e. the number of animals per group) were not estimated *a priori* since effect sizes in our system cannot be predicted. No predicted statistical outliers were removed since the presence or absence of these potential statistical outliers did not affect the overall interpretation. Mice that were euthanized early due to health concerns were excluded from analysis.

## Supplementary Material

1

Supplemental information can be found online at https://doi.org/10.1016/j.cell.2026.04.012.

## Figures and Tables

**Table:** KEY RESOURCES TABLE

REAGENT or RESOURCE	SOURCE	IDENTIFIER
Bacterial and virus strains		
*Enterotoxigenic Bacteroides fragilis* 86-5443-2-2	Mundy and Sears^161^	WZ1023
*B. fragilis* Δ*bft*	Chung et al.^30^	WZ1025
*B. fragilis* Δ*tdk*	This study	RF54
*B. fragilis* Δ*tdk* Δ*bft*	This study	RF59
*B. fragilis* Δ*tdk* Δ*bft*::pNBU2 (Cm^R^)	This study	YW249
*B. fragilis* Δ*tdk* Δ*bft*::pNBU2-*bft* (Cm^R^)	This study	YW624
*B. fragilis* Δ*tdk* Δ*bft*:: pNBU2_*catP-tetR*-P1T_DP-GH023 (Cm^R^)	This study	YW524
*B. fragilis* Δ*tdk* Δ*bft*::pNBU2_*catP-tetR*-P1T_DP-GH023-*bft* (Cm^R^)	This study	YW528
Non-enterotoxigenic *B. fragilis* ATCC 25285	Cato and Johnson^162^	WZ901
NTBF ATCC 25285 Δ*tdk*	This study	WZ916
NTBF ATCC 25285 Δ*tdk*::pNBU2 (Cm^R^)	This study	YW716
NTBF ATCC 25285 Δ*tdk*::pNBU2 (Cfx^R^)	This study	YW267
NTBF ATCC 25285 Δ*tdk*::pNBU2-*bft-1* (Cfx^R^)	This study	YW856
NTBF ATCC 25285 Δ*tdk*::pNBU2-*bft-2* (Cfx^R^)	This study	YW439
NTBF ATCC 25285 Δ*tdk*::pNBU2-*bft-3* (Cfx^R^)	This study	YW854
NTBF ATCC 25285 Δ*tdk* Δ*ldh1*:: pNBU2 (Cm^R^)	This study	RF447
NTBF ATCC 25285 Δ*tdk* Δ*asct*:: pNBU2 (Cm^R^)	This study	RF448
Non-enterotoxigenic *B. fragilis* TM4000	Tang et al.^70^	WZ861
NTBF TM4000::pNBU2 (Cm^R^)	This study	YW725
NTBF TM4000::pNBU2-*bft-2* (Cfx^R^)	This study	YW735
*B. fragilis* Δ*tdk* Δasct ::pNBU2 (Cfx^R^)	This study	ML207
*B. fragilis* Δ*tdk* Δasct ::pNBU2-*asct* (Cfx^R^)	This study	ML252
*B. fragilis* Δ*tdk* Δ*cydAB*	This study	RF252
*B. fragilis* Δ*tdk* Δ*cydAB*:: pNBU2-*cydAB* (Cm^R^)	This study	RF450
*B. fragilis* Δ*tdk* Δ*lutABC* Δ*ldh2*	This study	LS82
*B. fragilis* Δ*tdk* Δ*lutABC* Δ*ldh2* ::pNBU2-*lutABC* (Cm^R^), ::pNBU2-*ldh2* (Cfx^R^)	This study	RF435
*B. fragilis* Δ*tdk* Δ*ldh1*	This study	RF314
*B. fragilis* Δ*tdk* Δ*ldh1* ::pNBU2-*ldh1* (Cfx^R^)	This study	ML251
*B. fragilis* Δ*tdk* Δ*sucCD* Δ*asct*	This study	WZ1481
*B. fragilis* Δ*tdk* Δ*sucCD* Δ*asct* ::pNBU2-*sucCD* (Cm^R^), pNBU2-*asct* (Cfx^R^)	This study	RF449
*B. fragilis* Δ*tdk* Δ*sucAB*	This study	LS80
*E. coli* S17-1 λ*pir*; *zxx::*RP4 2-(Tet^r^::Mu) (Kan^r^::Tn7) λ*pir*	Simon et al.^163^	S17-1 λ*pir*
*B. fragilis* Korea 570	Chung et al.^82^	YW686
*B. fragilis* 1284	Sheahan et al.^164^	YW755
*B. fragilis* YCH46	Kuwahara et al.^165^	YW756
*B. fragilis* J38-1	Shumaker et al.^166^	YW757
*B. fragilis* I1345	Franco et al.^79^	YW758
*B. fragilis* CL03T12C07	Rakoff-Nahoum et al.^167^	YW759
*B. fragilis* CL05T12C13	Rakoff-Nahoum et al.^167^	YW760
*B. fragilis* CL07T12C05	Chatzidaki-Livanis et al.^168^	YW761
*Clinical B. fragilis* 24-0219395	This study	YW763
*Clinical B. fragilis* 24-0236579	This study	YW764
*Clinical B. fragilis* 24-0236580	This study	YW765
*Clinical B. fragilis* 24-0253831	This study	YW766
*Clinical B. fragilis* 24-0270355	This study	YW767
*Clinical B. fragilis* 25-0025361	This study	YW768
*Clinical B. fragilis* 25-0045109	This study	YW769
*Clinical B. fragilis* 25-0046408	This study	YW770
*Clinical B. fragilis* 25-0066227	This study	YW771
*Clinical B. fragilis* 25-0074525	This study	YW772
*Clinical B. fragilis* 25-0092798	This study	YW773
*BF-25*-0092798::pNBU2 (Cm^R^)	This study	YW866
*BF-25*-0092798::pNBU2::*bft-2* (Cm^R^)	This study	YW915
*Clinical B. fragilis* 25-0095685	This study	YW774
*Clinical B. fragilis* 25-0103606	This study	YW775
*Clinical B. fragilis* 25-0140735	This study	YW776
*Clinical B. fragilis* 25-0145162	This study	YW777
*Clinical B. fragilis* 25-0145372	This study	YW778
Recombinant DNA		
pKNOCK-*bla-ermGb*	Koropatkin et al.^169^	pKNOCK
pKNOCK-*bla-ermGb*::*tdk*	Koropatkin et al.^169^	pExchange-tdk
pKNOCK-*bla*::Promoter *ermGb*::*cfxA*	This study	pKNOCK-ETBF
pKNOCK-*bla*::Promoter *ermGb*::*cfxA*::*tdk*	This study	pEET
pNBU2-*bla-catP*	This study	pNBU2-Cm^R^
pNBU2-*bla-cfxA*	This study	pNBU2-Cfx^R^
pNBU2-*catP-tetR*-P1TDP-GH023	This study	pNBU2_*catP-tetR*-P1T_DP-GH023
pNBU2_*catP-tetR*-P1T_DP-GH023::*bft-2*	This study	pYW508
pNBU2-Cfx^R^::*P*_bft-1_-*bft-1*	This study	pYW827
pNBU2-Cm^R^::*P*_bft-2_ -*bft-2*	This study	pYW283
pNBU2-Cfx^R^::*P*_bft-2_ -*bft-2*	This study	pYW279
pNBU2-Cfx^R^::*P*_bft-3_ -*bft-3*	This study	pYW823
Upstream and downstream regions of ETBF *cydAB* in pEET	This study	pRF237
Upstream and downstream regions of ETBF *lutABC* in pEET	This study	pRF231
Upstream and downstream regions of ETBF *asct* in pEET	This study	pML191
Upstream and downstream regions of ETBF *ldh2* in pEET	This study	pML108
Upstream and downstream regions of ETBF *ldh1* in pEET	This study	pML102
Upstream and downstream regions of ETBF *sucAB* in pEET	This study	pRF317
Upstream and downstream regions of ETBF *sucCD* in pEET	This study	pRF247
Oligonucleotides		
Δ86_*tdk* 1 up: 5’- gctctagaactagtggatccGCTTTA CAAGAAGATCGAG-3’	This study	N/A
Δ86_*tdk* 1 down: 5’-gagctcccaTTGTATATGATCT TCTGAAAATAATAC-3’	This study	N/A
Δ86_*tdk* 2 up: 5’-tgccatttatTTGTATATGATCTTCT GAAAATAATAC-3’	This study	N/A
Δ86_*tdk* 2 down: 5’-tcgaattcctgcagcccgggATTCC TGAGTCAAAGACTC-3’	This study	N/A
ETBF Δtdk verification primer F: 5’- AGCGAGGAT TTAAGACTGTA-3’	This study	N/A
ETBF Δtdk verification primer R: 5’- GTTCCTCAA TACGTTGAGTT-3’	This study	N/A
Δ86_*lutABC* 1 up: 5’-gctctagaactagtggatccTGCA CATAAGCTGATTCTTC-3’	This study	N/A
Δ86_*lutABC* 1 down: 5’-gagctcccatATAAATGGCA TTGATATAACAGG-3’	This study	N/A
Δ86_*lutABC* 2 up: 5’-tgccatttatATGGGAGCTCATG GAGCG-3’	This study	N/A
Δ86_*lutABC* 2 down: 5’-tcgaattcctgcagcccgggCAG AGTCACTACTTAAATCAACTCGTAG-3’	This study	N/A
Δ86_*lutABC* verification primer F: 5’-CCAACGGGAT CAGGAAGAAA-3’	This study	N/A
Δ86_*lutABC* verification primer R: 5’-ACAGACGTGA CTGTGGTATTG-3’	This study	N/A
Δ86_*ldh2* 1 up: 5’- ggccgctctagaactagtggATAAATAA GGGGTTGACACC-3’	This study	N/A
Δ86_*ldh2* 1 down: 5’- agatatcggaTGTACCCGGCCTA TAAAAC-3’	This study	N/A
Δ86_*ldh2* 2 up: 5’- gccgggtacaTCCGATATCTTGCAA GAAC-3’	This study	N/A
Δ86_*ldh2* 2 down: 5’- gaattcctgcagcccgggGGATTCA CTTTACGGAAAGAC-3’	This study	N/A
Δ86_*ldh2* verification primer F: 5’- TGCGAATGCCAG AGAACCGG-3’	This study	N/A
Δ86_*ldh2* verification primer R: 5’- TTAATATCCACCA TTGACAT-3’	This study	N/A
Δ86_*ldh1* 1 up: 5’- gctctagaactagtggatccCTCGAAATG TTTTGATTTGAAAATC-3’	This study	N/A
Δ86_*ldh1* 1 down: 5’- ttatgatgaaAATCAGAAGCCTTT GGTG-3’	This study	N/A
Δ86_*ldh1* 2 up: 5’- gcttctgattTTCATCATAAGGTTT GGTAC-3’	This study	N/A
Δ86_*ldh1* 2 down: 5’- tcgaattcctgcagcccgggTTGGCATA ATCACTGAAATAC-3’	This study	N/A
Δ86_*ldh1* verification primer F: 5’- GAAAAAATGAGAGAA TAACC-3’	This study	N/A
Δ86_*ldh1* verification primer R: 5’- GATAAGCATCTGCGG CTGTTC-3’	This study	N/A
Δ86_*sucCD* 1 up: 5’- tagtggatccTTCAACGGTACGGA CGAAG-3’	This study	N/A
Δ86_*sucCD* 1 down: 5’- agggttcaccGTAAGTGGAGAA AATCTCCTTTG-3’	This study	N/A
Δ86_*sucCD* 2 up: 5’- ctccacttacGGTGAACCCTCGG AAATAC-3’	This study	N/A
Δ86_*sucCD* 2 down: 5’- gcagcccgggCGAAGCAAACC AATGAAATG-3’	This study	N/A
Δ86_*sucCD* verification primer F: 5’- GTATTCCCCGGT CAAGGTGC-3’	This study	N/A
Δ86_*sucCD* verification primer R: 5’- CTCCAATTGT CTCTAATTCC-3’	This study	N/A
Δ86_*asct* 1 up: 5’-cgctctagaactagtggatccATGGTGA AAGTGACAGC-3’	This study	N/A
Δ86_*asct* 1 down: 5’-cagccttgtcAGCGGAGACATGA AAAAC-3’	This study	N/A
Δ86_*asct* 2 up: 5’-tgtctccgctgacaaggCTGGCAGCTTC-3’	This study	N/A
Δ86_*asct* 2 down: 5’-cgaattcctgcagcccgggGTTTTCTTCT TCATCGAATGAAAGAACTC-3’	This study	N/A
Δ86_*asct* verification primer F: 5’-TTACTTTCCCTTTCTC GGTATTG-3’	This study	N/A
Δ86_*asct* verification primer R: 5’-TCACCGGCCTCTACC GAAGC-3’	This study	N/A
Δ86_*sucAB* 1 up: 5’-cgctctagaactagtggatcTTGACCTGCA CCCTTAACTCTAC-3’	This study	N/A
Δ86_*sucAB* 1 down: 5’-tcttttcatcATACCCGGCAGGTC CACC-3’	This study	N/A
Δ86_*sucAB* 2 up: 5’-tgccgggtatgatgaaAAGATATATAAA GCAGCCAAAGAGTTGC-3’	This study	N/A
Δ86_*sucAB* 2 down: 5’-cgaattcctgcagcccgggGATGCGG GGAGCTAAGCCTTG-3’	This study	N/A
Δ86_*sucAB* verification primer F: 5’- TCGTTTCTGTGGC TATCACA-3’	This study	N/A
Δ86_*sucAB* verification primer R: 5’- CCTGATCAATCCG CAGATGG-3’	This study	N/A
pNBU2 linearization F: 5’- CCCCGGGCTGCAGGAATT −3’	This study	N/A
pNBU2-flag linearization F: 5’- aggatgacgatgacaagtagccc cgggctgcaggaatt −3’	This study	N/A
pNBU2 linearization R: 5’- GATCCACTAGTTCTAGAGCGGC −3’	This study	N/A
pNBU2_*catP-tetR*-P1T_DP-GH023 linearization F: 5’- GTGGA TCCCCCGGGCTGC −3’	This study	N/A
pNBU2_*catP-tetR*-P1T_DP-GH023linearization R: 5’- GGTGTC TTTTCTTTTATATGTCTTTATTTCGTCTCTATCACTGATAGGG −3’	This study	N/A
BFT-2 ORF F: 5’- catataaaagaaaagacaccATGAAGAATGTAAAG TTACTTTTAATG −3’	This study	N/A
BFT-2 ORF R: 5’- ctgcagcccgggggatccacCTAATCGCCATCTG CTATTTC −3’	This study	N/A
Promoter-flag F: 5’- cgctctagaactagtggatcTATACGTATCCTGG ATTGAATTTC −3’	This study	N/A
Promoter-flag R: 5’- agtaactttacattcttcatTTTGTTAAAATTTAAA ATAAACATTGGTTTTATAATTG −3’	This study	N/A
Promoter and BFT-1 F: 5’- cgctctagaactagtggatcATACGTATC CCGGATTGG −3’	This study	N/A
Promoter and BFT-1 R: 5’- cgaattcctgcagcccggggCTAATCGC CATCTGCAGC −3’	This study	N/A
Promoter and BFT-2 F: 5’- cgctctagaactagtggatcTATACGTATC CTGGATTGAATTTC −3’	This study	N/A
Promoter and BFT-2 R: 5’- cgaattcctgcagcccggggCTAATCGC CATCTGCTATTTC −3’	This study	N/A
Promoter and BFT-3 F: 5’- cgctctagaactagtggatcCGGATTGGA TTTCGACATAG −3’	This study	N/A
Promoter and BFT-3 R: 5’- cgaattcctgcagcccggggCTAATCGC CATCTGCTATTTC −3’	This study	N/A
::J38-1_ *bft-1* up: 5’- cgctctagaactagtggatcATACGTATCCCG GATTGG −3’	This study	N/A
::J38-1_ *bft-1* down: 5’- cgaattcctgcagcccggggCTAATCGCC ATCTGCAGC-3’	This study	N/A
::Korea 570_ *bft-3* up: 5’- cgctctagaactagtggatcCGGATTG GATTTCGACATAG −3’	This study	N/A
::Korea 570_ *bft-3* down: 5’- cgaattcctgcagcccggggCTA ATCGCCATCTGCTATTTC −3’	This study	N/A
Mouse *Gapdh* qRT-PCR primers: 5’-TGTAGACCATGTA GTTGAGGTCA-3’; 5’-AGGTCGGTGTGAACGGATTTG-3’	Overbergh et al.^170^	N/A
Mouse *Cxcl1* qRT-PCR primers: 5’-TGCACCCAAACCG AAGTCAT-3’; 5’-TTGTCAGAAGCCAGCGTTCAC-3’	Godinez et al.^171^	N/A
Mouse *IfnG* qRT-PCR primers: 5’- TCAAGTGGCATAGAT GTGGAAGAA-3’; 5’- TGGCTCTGCAGGATTTTCATG −3’	Overbergh et al.^170^	N/A
Mouse *Il17* qRT-PCR primers: 5’- ATCGCTGCTGCCTTC ACTGTAG-3’; 5’- TGATGCTGTTGCTGCTGCTGAG-3’	This study	N/A
Mouse *Fgf15* qRT-PCR primers: 5’- ATGGCGAGAAAGT GGAACGG −3’; 5’- CTGACACAGACTGGGATTGCT −3’	This study	N/A
Mouse *Cyp7a1* qRT-PCR primers: 5’- CACCTTGAGGAT GGTTCCTATAAC −3’; 5’- CCAAAGGGTCTGGGTAGATTTC −3’	This study	N/A
Mouse *Cyp2c70* qRT-PCR primers: 5’- CAAGTCATCTCC TCCACCATAC −3’; 5’- CCAGGTTTCAGCACCAAATAAA −3’	This study	N/A
Mouse *Cyp2a12* qRT-PCR primers: 5’- CTGAGAACAAAGC CCTCTACTT −3’; 5’- CAACTTTCAGGAGGACCATACA −3’	This study	N/A
*B. fragilis bft* qRT-PCR primers: 5’- GGTTTCAACCGTCAGG TACA −3’; 5’- GCGAACTCATCTCCCAGTATAAA −3’	This study	N/A
*B. fragilis mpII* qRT-PCR primers: 5’- CCTCACACCCTTACAC ACTGG −3’; 5’- TGCATTCCAACATCCGGACC-3’	This study	N/A
*B. fragilis ldh1* qRT-PCR primers: 5’- AAGCCTAACAGTCCGT GCAG −3’; 5’- ACTGATGCTCTCGCTCAACC −3’	This study	N/A
*B. fragilis cydA* qRT-PCR primers: 5’- AAGGACAAAATGGTGC CGGA −3’; 5’- AGGCATCCACCTTACGTTCG −3’	This study	N/A
*B. fragilis cydB* qRT-PCR primers: 5’- CCAGTTCACGCTCAAG ACCA −3’; 5’- TCTTCCGGTTGTCGATGCTG −3’	This study	N/A
Chemicals, peptides, and recombinant proteins		
(NH_4_)_2_SO_4_	Sigma-Aldrich	A4915
2-Deoxy-D-glucose ≥99%	Fisher Scientific	AC111980050
5 beta -CHOLANIC ACID-3-ONE	Steraloids	C1750-000-50mg
5-fluoro-2-deoxy-uridine	Ark Pharm	AK-24802
Acid-Phenol:Chloroform:IAA, pH 4.5 (125:24:1)	Ambion	AM9720
Agar	Fisher Scientific	BP1423
Anhydrotetracycline (aTC)	Cayman Chemical Company	10009542
Anti rabbit IgG (H+L), F(ab’)2 Fragment (Alexa Fluor^®^ 488 Conjugate)	Cell Signaling Technology	4412S
Anti-CD326 (Ep-CAM) Rat Monoclonal Antibody (APC (Allophycocyanin)) [clone: G8.8], Size=100 μg	BioLegend	118214; RRID: AB_1134102
Anti-Mouse CD45, PE-Cyanine7 (30-F11)	TonBo Biosceinces	60-0451-U025; RRID: AB_2621848
Anti-Mouse IL-17A (17F3)	BioXCell	BE0173; RRID: AB_10950102
Anti-Mouse ROR γt (B2D)	Invitrogen	12-6981-80; RRID: AB_10805392
Anti-Mouse TCR-beta, APC Cyanine7 (H57-597)	Cytek	25-5961-U025; RRID: N/A
Anti-SLC10A2 Rabbit Polyclonal Antibody, Size=150 μL	Proteintech	25245-1-AP; RRID: AB_2879986
Bile salts	Sigma-Aldrich	B8756-100G
BioReagent Puromycin dihydrochloride, ≥98% (HPLC), From Streptomyces alboniger	Sigma-Aldrich	P8833-100MG
Biotin Anti-Mouse TCR γ/δ (GL3)	BioLegend	118103
Brain-heart-infusion (BHI) media	Becton Dickinson	211059
Carbenicillin disodium salt	VWR	J358-1G
Cefoxitin sodium salt	Fisher Scientific	VC00021-5G
Chenodeoxycholic acid	Cambridge Isotope Laboratories	ULM-9540-0.05
Chenodeoxycholic acid (2,2,4,4-d4, 98%)	Cambridge Isotope Laboratories	DLM-6780-0.05
Chloramphenicol (Crystalline Powder)	Fisher Scientific	BP904-100
Chloroform, anhydrous, >99%	Sigma-Aldrich	288306-1L
Cholic acid	Cambridge Isotope Laboratories	ULM-9543-0.05
Cholic acid (2,2,4,4-D4, 98%)	Cambridge Isotope Laboratories	DLM-2611-0.05
Clindamycin Hydrochloride Monohydrate	TCI Chemicals	C2256-25G
Collagenase from *Clostridium histolyticum*	Sigma-Aldrich	C2139-500MG
Curdlan from Alcaligenes faecalis	Sigma-Aldrich	C721-5G
Dansyl hydrazine	Sigma-Aldrich	635928-500MG
Deoxycholic Acid (2,2,4,4-d4, 98%)	CDN Isotopes	D-2941
Dextran Sulfate Sodium Salt, MW ca 40,000	Thermo Fisher Scientific	J63606-22
Difco LB broth-Miller	Becton Dickinson	Cat# 244620
Dilution Buffer, pH 7.0	BioXCell	IP0070
1-(3-Dimethylaminopropyl)-3-ethy; carbodiimide hydrochloride, >98%	Thermo Fisher Scientific	A10807.06
Dimethyl sulfoxide (DMSO)	Corning	MT-25950CQC
DL-Dithiothreitol solution,1 m in H2O	Sigma-Aldrich	646563-0X.5ML
DL-Dithiothreitol solution,1 m in H2O	Sigma-Aldrich	646563-10X.5ML
DNA-free DNA removal Kit	Invitrogen	AM1906
DNase 1	Sigma-Aldrich	D4527-20KU
Dry Powder Milk, nonfat	RPI	M17200-500
EDTA Disodium Salt	RPI	E57020-1000.0
Erythromycin	Sigma-Aldrich	E5389-1G
FABP6 Polyclonal antibody	Proteintech	13781-1-AP
Fetal Bovine Serum, 500 mL, Regular, USDA Safety Tested (Heat Inactivated)	Corning	35-011-CV
Gentamicin	Sigma-Aldrich	G1397-100ML
Ghost Dye(tm) Violet 510 Viability Dye	Cell Signaling Technology	59863S
Girard’s Reagent P	TCI Chemicals	G0030
Glucose	Fisher Chemical	D14-212
Glyceryl tributyrate,≥99%	Sigma-Aldrich	T8626-100ML
Glycocholic Acid (2,2,4,4-d4, 98%)	CDN Isotopes	D-3878
Goat Anti-Mouse IgG Polyclonal Antibody (IRDye 800CW), Size=500 μg	Neta Scientific	926-32210
GolgiStop protein transporter inhibitor	BD Bioscience	554724
Hemin, from Porcine, >96% (HPLC)	Sigma-Aldrich	51280-1G
HBSS (10X), no calcium, no magnesium, no phenol red	Gibco	14185052
HBSS, calcium, magnesium	Gibco	24020117
Hypoxyprobe Omni Kit (200 mg pimonidazole HCl plus 1 unit of 2627 rabbit antisera)	Hypoxyprobe	HP3-200Kit
Hypoxyprobe-Green Kit (100 mg pimonidazole HCl plus 1 unit of 4.3.11.3 mouse FITC-MAb)	Hypoxyprobe	HP6-100Kit
Isolithocholic Acid	Cayman Chemical Company	29545
Kanamycin	Fisher Scientific	BP906-5
KH_2_PO_4_	Fisher Scientific	P285
L-methionine	Sigma-Aldrich	M9625
LGK-974 5mg	Selleck Chemicals	S7143
LI-COR IRDye 800cw Donkey Anti-Rabbit Igg Secondary Antibody, 0.1 mg	Neta Scientific	925-32213
Lithocholic acid	Cambridge Isotope Laboratories	ULM-9559-0.05
Lithocholic acid (2,2,4,4-D4, 98%)	Cambridge Isotope Laboratories	DLM-9560-0.05
Medchemexpress LLC MARIMASTAT 5MG SDP	Fisher Scientific	50-187-2596
Menadione (Vitamin K_3_)	Sigma-Aldrich	M5625-25G
Methanol, anhydrous, 99.8%	Sigma-Aldrich	322415-1L
Mouse Cot-1 DNA, 1μg/μL	Invitrogen	18440016
mouse IgG1 isotype control (MOPC-21)	BioXCell	BE0083
NaCl	RPI	S23020
NaHCO_3_	Sigma-Aldrich	S6014
Oligomycin A, ≥99% (HPLC)	Sigma-Aldrich	75351-5MG
Paraformaldehyde Aqueous Solution, 16%	Electron Microscopy Sciences	15710
PE anti-Puromycin Antibody	BioLegend	381504
Percoll	Cytiva	17089101
Proteinase K, Molecular Biology Grade, 800 U/mL	New England Biolabs (NEB)	P8107S
Protoporphyrin IX	Sigma-Aldrich	P8293
Pyridine Hydrochloride, 98%	Sigma-Aldrich	243086
Rat Anti-Mouse Foxp3, Alexa Fluor^®^ 647 (MF23)	BD Pharmingen	560401
Rat Anti-Mouse IL-17A, BV421 (TC11-18H10)	BD Horizon	563354
redFluor^™^ 710 Anti-Mouse CD45 (30-F11)	Tonbo Biosciences	80-0451-U100
Resazurin	Sigma-Aldrich	R7017
RIPA Lysis and Extraction Buffer	Thermo Fisher Scientific	89900
RPMI Medium 1640 (1X)	Gibco	22400-089
Sodium Dodecyl Sulfate, 20%	Invitrogen	AM9820
Sodium L-lactate,≥99.0%	Sigma-Aldrich	71718-10G
Streptavidin PE-eFluor 610, eBioscience	Invitrogen	61-4317-82
Streptomycin Sulphate USP Grade	G-Biosciences	RC-196
Tcr gamma/delta Monoclonal Antibody (eBioGL3 (GL-3, GL3)), eFluor 450, eBioscience	Invitrogen	48-5711-82
TRI reagent	Molecular research center	TR118
Tryptone	Fisher Scientific	BP1421
Ursodeoxycholic Acid (2,2,4,4-d4)	CDN Isotopes	D-3819
α-pimonidazole rabbit antisera	Hypoxyprobe	Pab2627
Critical commercial assays		
384-well Low Flange White Flat Bottom Polystyrene TC-treated Microplates, with Lid, Sterile	Corning	3570
Bacteria RNAprotect	Qiagen	76506
BacTiter-Glo Microbial Cell Viability Assay	Promega	G8230
Foxp3 / Transcription Factor Staining Buffer Set	Invitrogen	00-5523-00
Gibson Assembly Cloning Kit	NEB	E2611
Illumina DNA Prep kit	Illumina	20060060
KAPA HiFi Hotstart ReadyMix	Roche	KK2601
Lysing Matrix B	MP Bio	1169110-CF
Lysing Matrix C	MP Bio	1169120-CF
M.O.M. (Mouse on Mouse) Immunodetection Kit, Basic	VECTOR laboratories	BMK-2202
Mini-Protean 4-20% TGX Stain-Free Gel	Bio-Rad	4568095
PowerSoil Pro Kit	Qiagen	47014
PowerUp SYBR Green Master Mix	Applied Biosystems	A25742
Q5 Hot Start 2x Master Mix	NEB	M0494L
Quick-DNA Fungal/Bacterial Miniprep Kit	Zymo Research	D6005
RNAlater^™^ Stabilization Solution	Invitrogen	AM7020
RNeasy mini kit	Qiagen	74104
RNeasy PowerFecal Pro Kit	Qiagen	78404
SuperScript VILO cDNA Synthesis Kit	Invitrogen	11754050
SYBR Green qPCR Master Mix	Life Technologies	4309155
TaqMan reverse transcription reagents	Invitrogen	N8080234
Trans-Blot Turbo RTA Mini 0.2 μm PVDF Transfer Kit	Bio-Rad	1704272
Turbo DNA-free Kit	Ambion	AM1907
Twist Standard Hybridization Reagents Kit, v1	Twist Bioscience	104179
Deposited data		
Hybrid selection RNAseq	NCBI BioProject database	PRJNA1269620
ETBF bacterial RNAseq	The European Nucleotide Archive	PRJEB89361 (secondary accession ERP172389)
Mouse tissue bulk RNAseq	The European Nucleotide Archive	PRJEB89362 (secondary accession ERP172390)
16S rRNA sequencing	The European Nucleotide Archive	PRJEB89357 (secondary: ERP172384)
Whole genome sequencing	The European Nucleotide Archive	PRJEB107014 (secondary: ERP188052)
Untargeted metabolomics data	NIH Common Fund’s National Metabolomics Data Repository	ST003919, project DOI: https://doi.org/10.21228/M8PR93)
Experimental Models: Organisms/Strains		
SPF C57BL/6 mice (wild-type)	The Jackson Laboratory	Cat# 000664
Software and algorithms		
BBMap	DOE Joint Genome Institute	V38.90
BigOmics Analytics	Akhmedov et al.^172^	V3.2.26
BioRender	BioRender.com	N/A
Bowtie2	Langmead and Salzberg^173^	V2.5.4
BV-BRC platform	UChicago	V3.55.17
CFX Maestro	Bio-Rad	V2.3
COBRA	MATLAB	V2.13.3
DESeq2	Love et al.^174^	V1.48.0
Excel for Mac	Microsoft	V16.70
FlowJo	FlowJo	V10.10
Ggtree	Yu et al.^175^	V3.16.0
Image J	Fiji	1.54p
MetaboAnalyst 5.0	Pang et al.^176^	V5.0
NCBI Datasets Command Line Tool	O’Leary et al.^177^	V16.40.1
NIS-Elements	Nikon	V6.10.01
Odyssey Western Blot Image Studio	LI-COR	V6.0
OrthoFinder	Emms and Kelly^178^	V3.0.1b1
Prism	Graph Pad	V10.4.2
Progenesis QI	Non-Linear Dynamics	V3.0
QIIME2	Bolyen et al.^179^	V2024.5
SILVA Database	Quast et al.^180^	V132
Thermo Xcalibur	Thermo Fisher Scientific	V2.0.7 SP1
Thermo LCQuan	Thermo Fisher Scientific	V2.7

## Data Availability

Data reported in this paper will be shared by the lead contact upon request. This study did not generate original code. The hsRNA-seq dataset has been deposited in the NCBI BioProject database under accession number NCBI: PRJNA1269620. ETBF and mouse tissue bulk RNA-seq data have been deposited in the European Nucleotide Archive (ENA) under accession numbers ENA: PRJEB89361 (secondary accession ERP172389) and ENA: PRJEB89362 (secondary accession ERP172390), respectively. The 16S rRNA gene sequencing data have been deposited in ENA under accession number ENA: PRJEB89357 (secondary accession ERP172384). Whole-genome sequencing reads have been deposited in ENA under accession number ENA: PRJEB107014 (secondary accession ERP188052). Untargeted metabolomics data have been deposited in the NIH Common Fund’s National Metabolomics Data Repository (Metabolomics Workbench) under Study ID ST003919 and are accessible via the project doi: https://doi.org/10.21228/M8PR93. Any additional information required to reanalyze the data reported in this paper is available from the lead contact upon request.

## Highlights

- A classically anaerobic pathogen creates and thrives in a localized oxidative niche
- BFT rewires epithelial metabolism from oxidative phosphorylation to fermentation
- Host metabolic rewiring increases lactate and oxygen in the ETBF niche
- BFT enables ETBF to adapt to this oxidative niche and promote colonization
