# Supplementary material for: An anaerobic pathogen rewires host metabolism to fuel oxidative growth in the inflamed gut
Source: Cell. Author manuscript; Available in PMC 2026 May 20. (PMC13185528; doi:10.1016/j.cell.2026.04.012)
Supplement: 1 [file NIHMS2171806-supplement-1.pdf]

CellPress
OPEN ACCESS

Supplemental figures

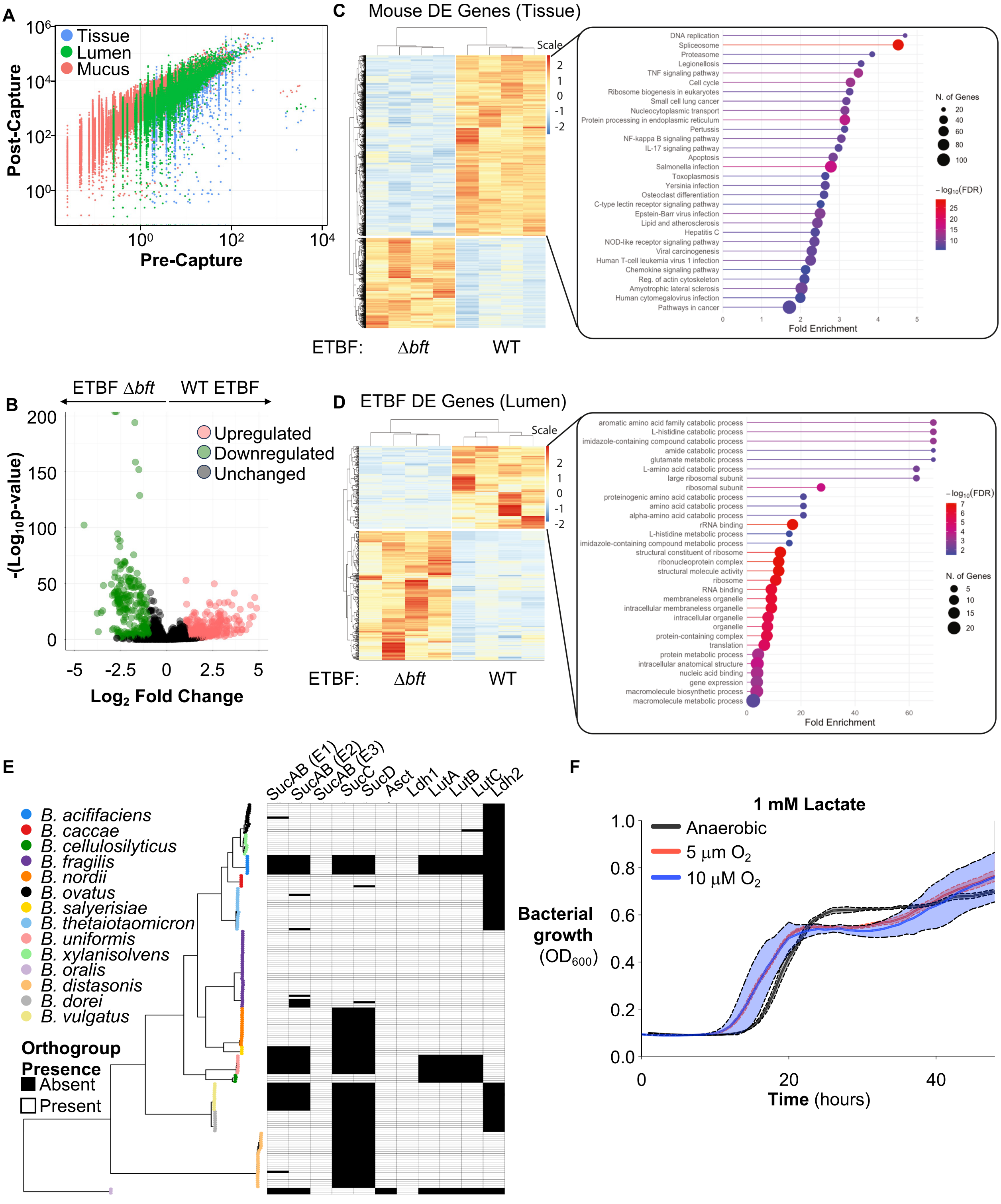

(legend on next page)

Figure S1. ETBF engages oxidative metabolism during intestinal colonization, related to Figure 1

(A–D) C57BL/6 mice were colonized with either wild-type (WT) ETBF or an isogenic Δ*bft* mutant for 7 days. hsRNA-seq was performed on luminal contents, mucus, and cecal tissue to profile the ETBF transcriptome during infection. (A) Correlation between pre-captured and post-captured ETBF transcriptomes. (B) Volcano plot of post-captured ETBF transcriptome from the intestinal lumen. (C and D) Gene Ontology (GO) enrichment analysis of (C) pre-captured mouse transcriptome and (D) post-captured ETBF transcriptome in the intestinal lumen.

(E) Conservation of genes involved in lactate oxidation and central metabolism across the *Bacteroidetes* phylum.

(F) The ETBF WT strain in semi-defined medium (SDM) supplemented with 1 mM lactate was exposed to indicated levels of O_2. Bacterial growth was measured by OD_{600}. Connected lines and shaded areas represent the mean and SEM.

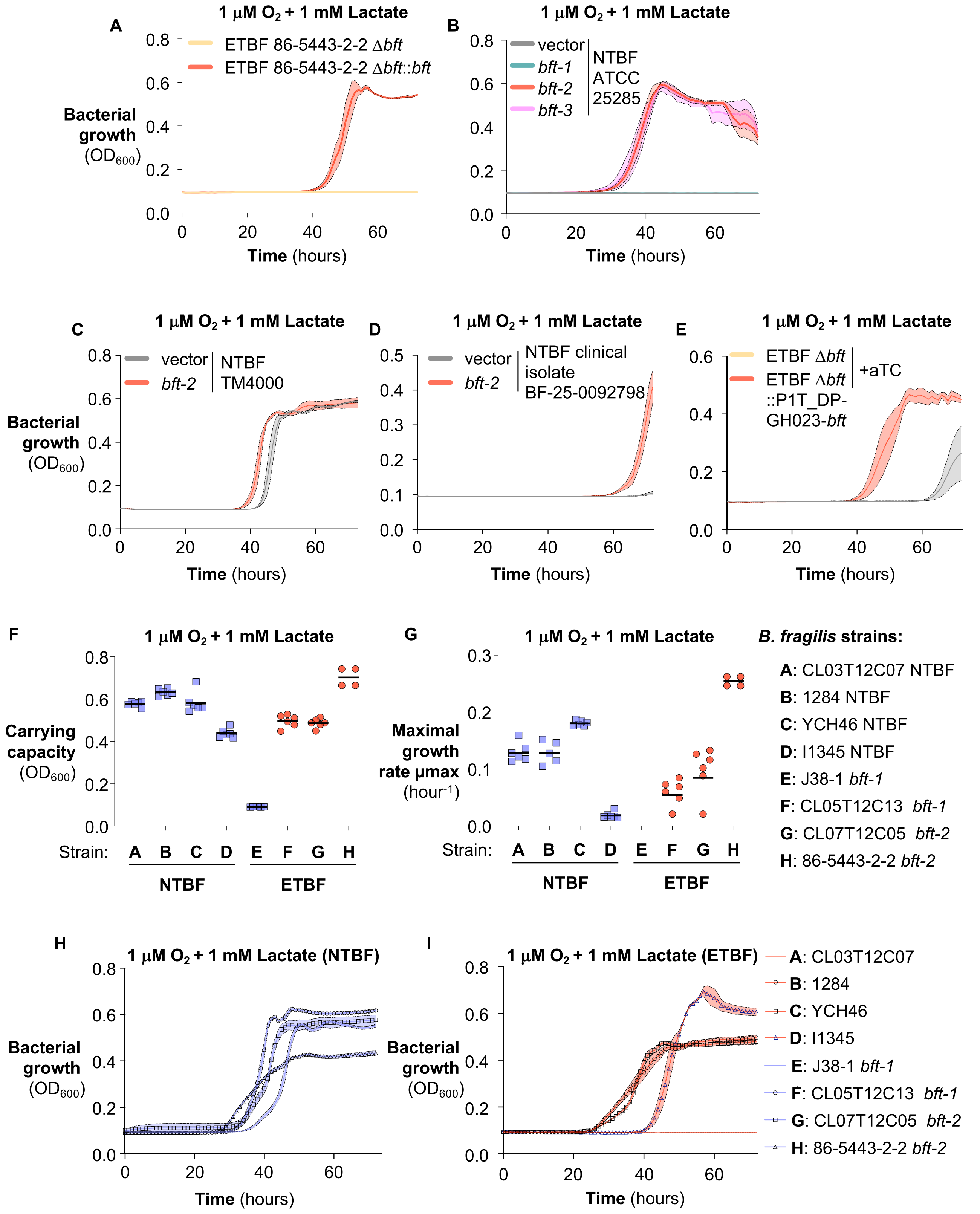

(legend on next page)

Figure S2. BFT promotes growth of *B. fragilis* under hypoxic conditions, related to Figure 1

(A–I) The indicated non-toxigenic (NTBF) or enterotoxigenic (ETBF) *B. fragilis* strains were cultured in medium supplemented with 1 mM lactate and exposed to 0.1% O_2. Bacterial growth was monitored by OD_{600}. Connected lines and shaded regions denote the mean ± SEM. (F) Maximal specific growth rate (μmax; h^{-1}), and (G) carrying capacity (final OD_{600}) were calculated from growth curves of NTBF (blue) and ETBF (red)strains. Horizontal bars denote the mean. (H and I) Growth curves for NTBF (H) and ETBF (I) strains grown under identical conditions. Shaded regions indicate mean ± SEM. Strain E (J38-1 *bft-1*) exhibited no detectable growth under these conditions and is denoted as N.G. (no growth).

CellPress
OPEN ACCESS

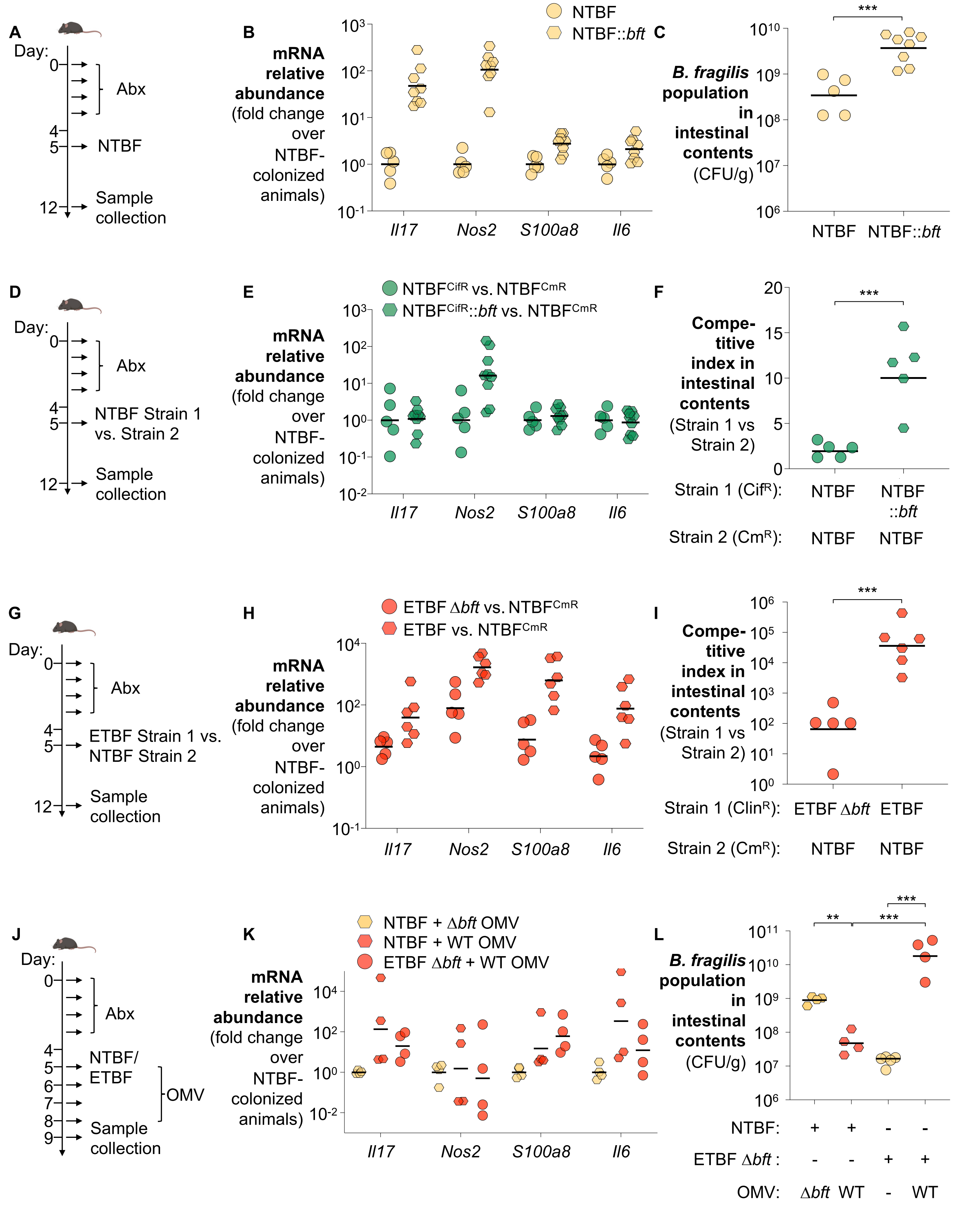

(legend on next page)

Figure S3. BFT reshapes the intestinal environment to selectively benefit *bft*-expressing *B. fragilis*, related to Figure 1

(A–C) Groups of antibiotic-pretreated C57BL/6 mice were colonized with either the WT non-toxigenic *B. fragilis* (NTBF) strain NCTC 9343 or an isogenic derivative expressing *bft* under its native promoter (NTBF::*bft*). Intestinal contents and tissue were collected at 7 days post inoculation. (A) Experimental schematic. (B) Transcript abundance of the indicated inflammatory markers quantified by real-time quantitative PCR. (C) Bacterial abundance quantified by selective plating.

(D–I) Groups of antibiotic-pretreated C57BL/6 mice were colonized with mixtures of (D–F) NTBF::*bft* and NTBF or (G–I) ETBF and NTBF. Intestinal contents and tissue were collected at 7 days post inoculation. (D and G) Experimental schematic. (E and H) Transcript abundance of the indicated inflammatory markers quantified by real-time quantitative PCR. (F and I) Competitive indices of NTBF::*bft* versus NTBF and ETBF versus NTBF quantified by selective plating.

(J–L) Groups of antibiotic-pretreated C57BL/6 mice were colonized with either the WT NTBF strain ATCC 25285 or an isogenic ETBF Δ*bft* mutant. Equal amounts of cell-free outer membrane vesicles (OMVs) derived from either WT ETBF or the corresponding Δ*bft* mutant were administered twice daily for 4 days. Intestinal contents and tissues were collected at 5 days post inoculation. (J) Experimental schematic. (K) Transcript abundance of the indicated inflammatory markers quantified by real-time quantitative PCR. (L) Abundance of the indicated strains quantified by selective plating. Bars represent the geometric means. n.s., not significant; $^{**}p < 0.01$; $^{***}p < 0.001$.

CellPress
OPEN ACCESS

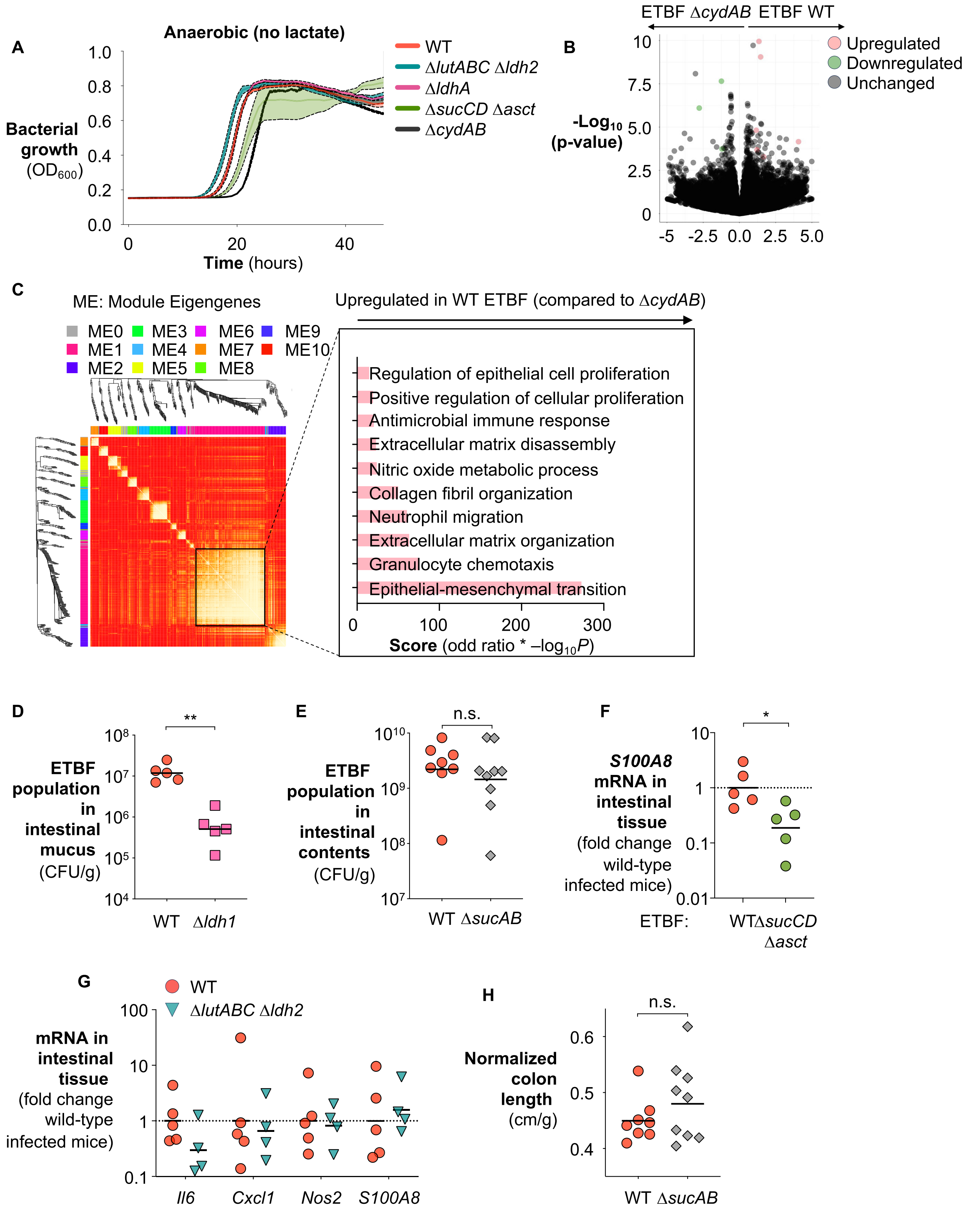

(legend on next page)

Figure S4. Oxygen respiration and oxidative metabolism contribute to ETBF colonization and inflammation, related to Figures 1 and 2

(A) Growth of the indicated ETBF strains in medium containing 1 mM lactate under 0.1% O_2. Bacterial growth was monitored by OD_{600}. Lines and shaded regions denote mean ± SEM.

(B and C) C57BL/6 mice were colonized with WT ETBF or the isogenic Δ*cydAB* mutant for 7 days. (B) Volcano plot of differentially expressed genes in the cecal transcriptome. (C) Weighted gene co-expression network analysis (WGCNA) of the cecal transcriptome.

(D–H) C57BL/6 mice were colonized with WT ETBF or the indicated mutant strains for 3 days. Bacterial populations were quantified in (D) intestinal mucus or (E) lumen by selective plating. Transcript levels of intestinal inflammatory cytokines were measured by real-time quantitative PCR in mice colonized by (F) Δ*sucCD* Δ*asct* or (G) Δ*lutABC* Δ*ldh2* mutants. (H) Colon length of Δ*sucAB*-colonized mice. Bars represent the geometric means. n.s., not significant; *$p < 0.05$; **$p < 0.01$.

CellPress
OPEN ACCESS

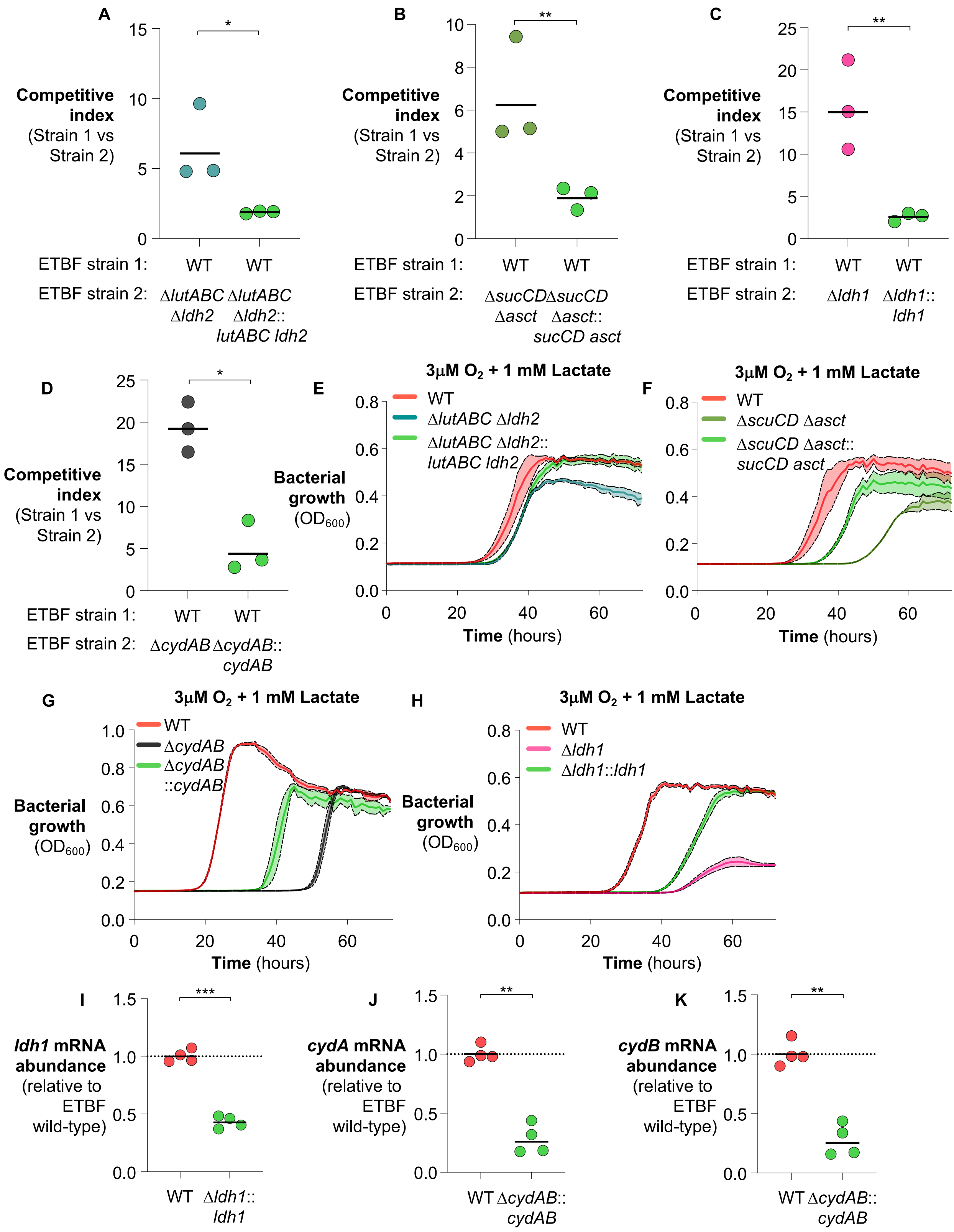

(legend on next page)

Figure S5. Genetic complementation rescues fitness defects of metabolic deletion mutants under hypoxic conditions, related to Figure 2

(A–D) WT ETBF and the indicated non-polar deletion mutants or their corresponding complemented strains were mixed at a 1:1 ratio and cultured in BHI supplemented with 1 mM lactate under hypoxic conditions (3 μM O_2). Competitive indices were quantified by selective plating.

(E–H) WT ETBF, deletion mutants, or the corresponding complemented strains were cultured individually in BHI supplemented with 1 mM lactate under hypoxic conditions (3 μM O_2), and bacterial growth was monitored by OD_{600}. Connected lines and shaded areas represent the mean and SEM.

(I–K) WT ETBF or the indicated complemented strains were cultured in BHI supplemented with 1 mM lactate under hypoxic conditions (3 μM O_2). Transcript abundance of the indicated genes was quantified by real-time quantitative PCR. Bars represent the geometric means. *$p < 0.05$; **$p < 0.01$; ***$p < 0.001$.

CellPress
OPEN ACCESS

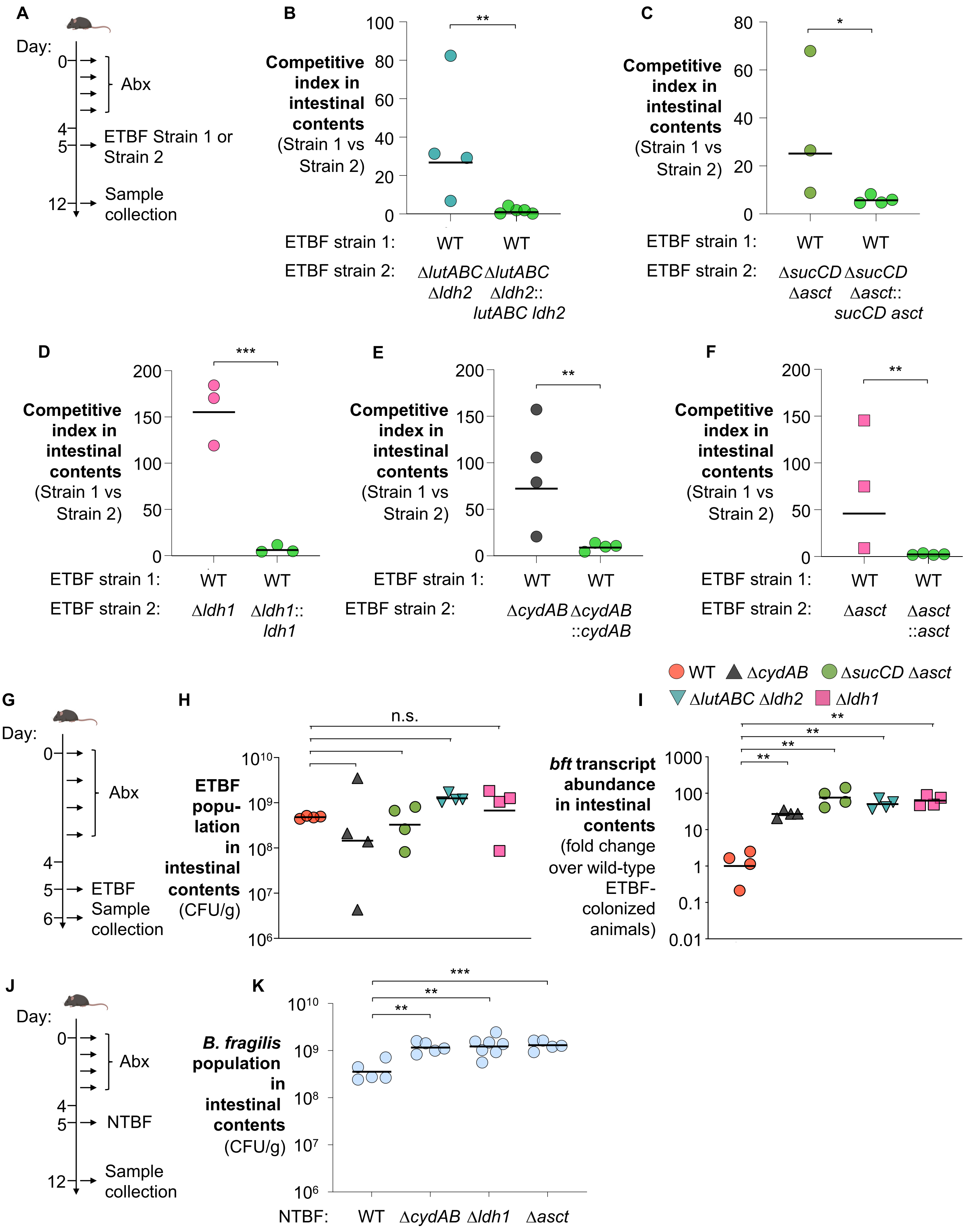

(legend on next page)

Figure S6. Genetic complementation rescues fitness defects of metabolic deletion mutants in the inflamed intestine, related to Figure 2

(A–F) Groups of antibiotic-pretreated C57BL/6 mice were colonized with mixtures of WT ETBF and the indicated deletion mutants or corresponding complemented strains. Intestinal contents were collected at 7 days post inoculation. (A) Experimental schematic. (B–F) Competitive indices of ETBF WT versus the indicated strains, determined by selective plating.

(G–I) Groups of antibiotic-pretreated C57BL/6 mice were colonized with indicated ETBF strains. Intestinal contents collected at 1 day post inoculation. (G) Experimental schematic. (H) ETBF abundance in intestinal contents quantified by selective plating. (I) *bft* expression measured by real-time quantitative PCR, normalized to the metalloprotease gene *mp2*.

(J and K) Groups of antibiotic-pretreated C57BL/6 mice were colonized with mixtures of WT NTBF or the indicated deletion mutants. Intestinal contents were collected at 7 days post inoculation. (J) Experimental schematic. (K) Abundance of indicated NTBF strains in the intestinal contents determined by selective plating. Bars represent the geometric mean. *$p < 0.05$; **$p < 0.01$; ***$p < 0.001$; n.s., not significant.

CellPress
OPEN ACCESS

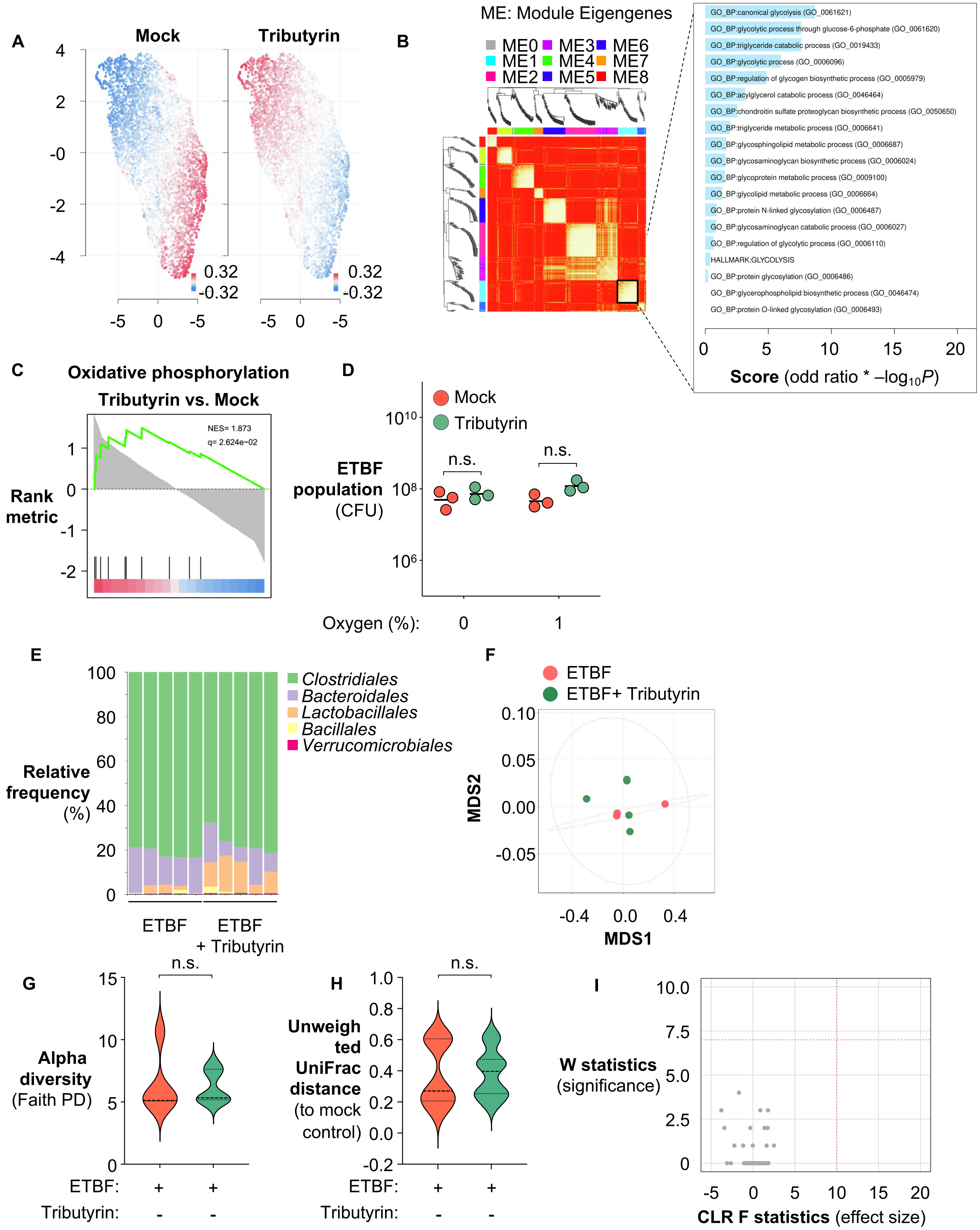

(legend on next page)

Figure S7. BFT-driven metabolic reprogramming of colonocytes promotes ETBF colonization, related to Figure 3

(A–C) C57BL/6 mice colonized with ETBF were treated with either tributyrin or vehicle control for 7 days. (A) Feature-level clustering, (B) weighted gene co-expression network analysis (WGCNA), and (C) gene set enrichment analysis of the cecal transcriptome.

(D) ETBF was cultured in brain heart infusion (BHI) medium supplemented with tributyrin and exposed to the indicated oxygen levels. Bacterial fitness was assessed by plating on selective agar.

(E–I) C57BL/6 mice colonized with ETBF were treated with tributyrin or vehicle control. After 7 days, cecal contents were collected for DNA extraction and 16S rDNA sequencing to profile gut microbiota composition. (E) Order-level microbiota composition. (F) Multidimensional scaling (MDS) analysis of microbiota composition. (G and H) Violin plots showing (G) alpha diversity (Faith's phylogenetic diversity) and (H) beta diversity (unweighted UniFrac distance). (I) Volcano plot from analysis of composition of microbiomes (ANCOM), with dotted lines indicating minimal cutoff values for differentially abundant taxa. Bars represent the geometric means. n.s., not significant. For (G) and (H), the central thick dotted line indicates the median, while the upper and lower dotted lines correspond to the first and third quartiles (the 25th and 75th percentiles) of the data.

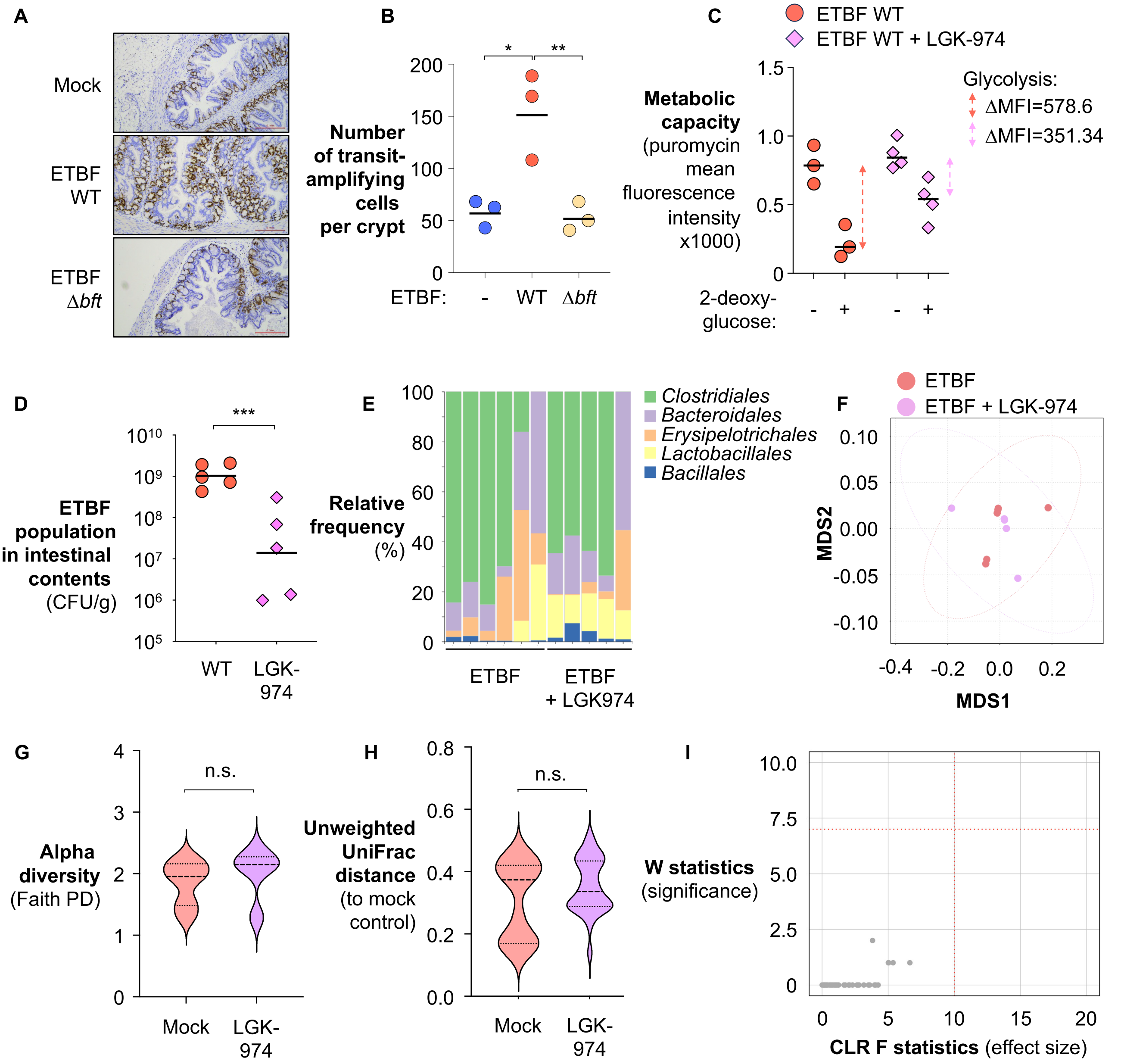

Figure S8. Disruption of epithelial processes promotes ETBF colonization and inflammation, related to Figure 4

(A and B) C57BL/6 mice were intragastrically challenged with WT ETBF, the isogenic Δ*bft* mutant, or remained uninfected for 7 days. TA epithelial cells were identified by Ki67 immunostaining. Scale bar, 100 μm. (A) Representative Ki67-stained intestinal tissue sections. (B) Quantification of Ki67⁺ non-stem cells per crypt.

(C) C57BL/6 mice colonized with ETBF WT strain were treated with the Wnt/β-catenin inhibitor LGK-974 or vehicle control. After 7 days, metabolic activity and glycolytic dependency in gut epithelial cells was quantified using SCENITH assay.

(D) C57BL/6 mice colonized with ETBF WT strain were treated with the Wnt/β-catenin inhibitor LGK-974 or vehicle control for 3 days. ETBF abundance was determined by plating on selective media.

(E–I) C57BL/6 mice colonized with ETBF WT strain were treated with the Wnt/β-catenin inhibitor LGK-974 or vehicle control. After 7 days, cecal contents were collected, DNA was extracted, and 16S rDNA sequencing was performed to profile gut microbiota composition. (E) Order-level microbiota composition. (F) Multidimensional scaling (MDS) analysis of microbiota composition. (G and H) Violin plots showing (G) alpha diversity (Faith's phylogenetic diversity) and (H) beta diversity (unweighted UniFrac distance). (I) Volcano plot from analysis of composition of microbiomes (ANCOM), with dotted lines indicating minimal cutoff values for differentially abundant taxa. For (B)–(D), bars represent the geometric means. *$p < 0.05$; **$p < 0.01$; ***$p < 0.001$. n.s., not significant. For (G) and (H), the central thick dotted line indicates the median, while the upper and lower dotted lines correspond to the first and third quartiles (the 25th and 75th percentiles) of the data.

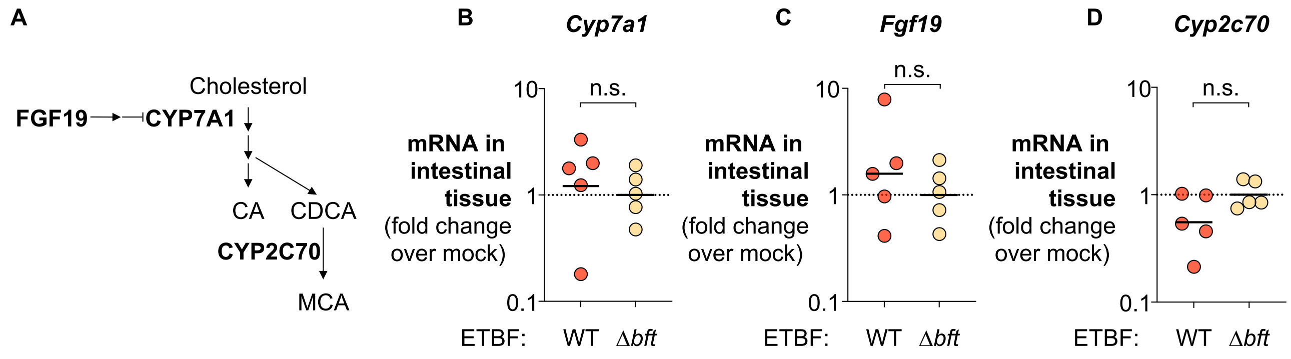

Figure S9. BFT depletes bile acids in the gut, related to Figure 5

(A–D) C57BL/6 mice were colonized with either WT ETBF or the isogenic Δ*bft* mutant. 7 days later, liver tissue was collected and RNA extracted. (A) Schematic for regulation of bile acid biosynthesis pathway in the liver. (B) *Cyp7a1*, (C) *Fgf19*, and (D) *Cyp2c70* mRNA levels in the liver quantified by real-time quantitative PCR. Bars represent the geometric means. n.s., not significant.

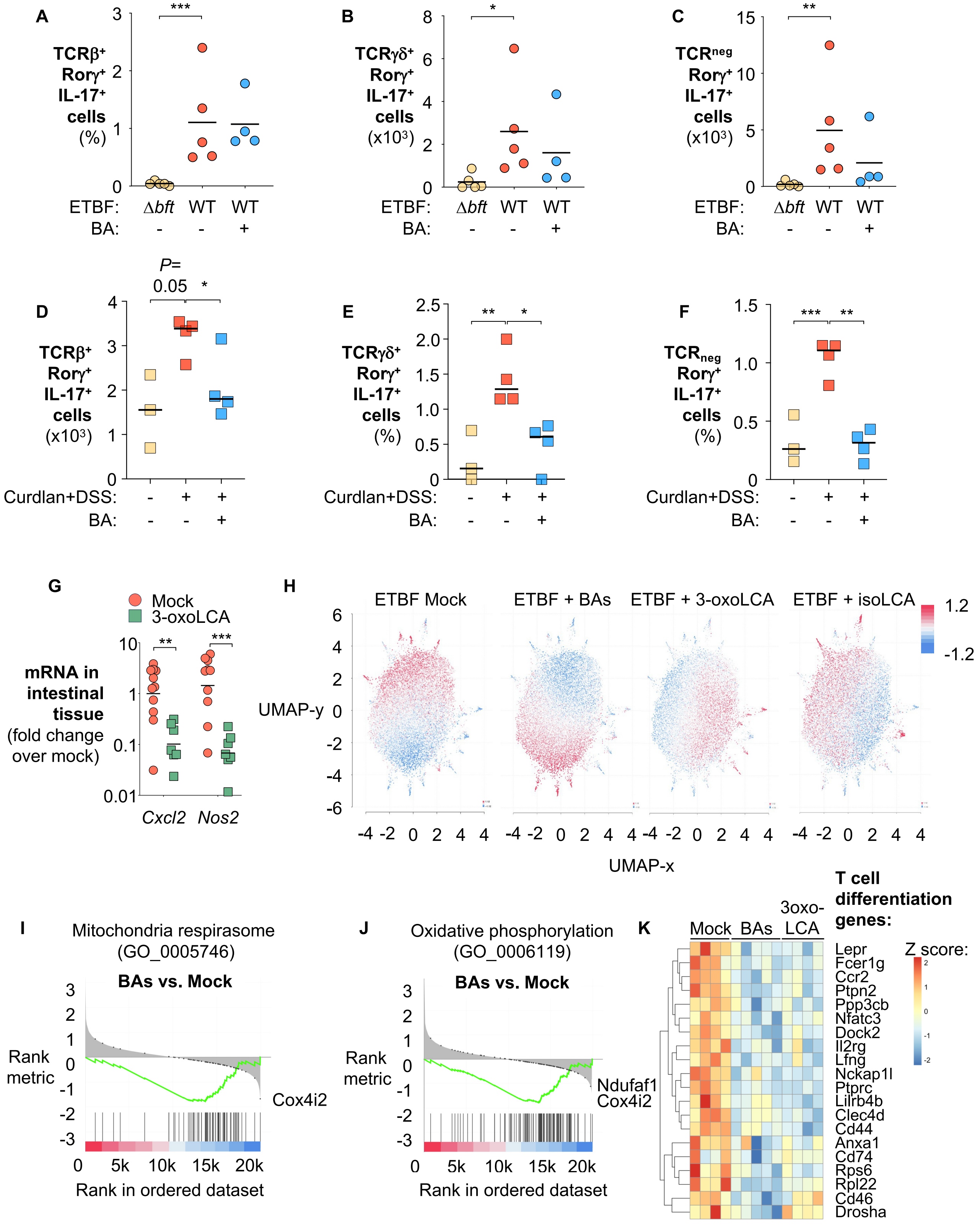

(legend on next page)

Figure S10. ETBF rewires colonocyte metabolism by impacting bile acid abundance, related to Figure 6

(A–C) Groups of C57BL/6 mice were colonized with WT ETBF or the isogenic Δ*bft* mutant and given either vehicle control or primary bile salts in drinking water for 7 days. IL-17-expressing IELs were profiled by flow cytometry. Total number of IL-17-producing (A) innate lymphoid cells and (B) γδ T cells, and the frequency of (C) T_H17 cells.

(D–F) Groups of C57BL/6 mice were mock treated or administered the yeast β-glucan curdlan for 2 weeks, followed by dextran sulfate sodium (DSS) treatments for 5 days. A subset of mice received bile acids in the drinking water for 5 days prior to tissue collection. Intestinal immune cells were then isolated and IL-17-producing populations were quantified by flow cytometry. (D–F) Numbers of IL-17$^+$ cells within TCRβ$^+$, TCRγδ$^+$, and TCR$^-$ immune cell populations.

(G–K) Groups of ETBF-colonized C57BL/6 mice were administered the indicated bile acids intragastrically for 7 days. (G) mRNA levels of inflammatory cytokines in intestinal tissue determined by real-time quantitative PCR. (H) Feature-level clustering, (I and J) gene set enrichment analysis, and (K) pathway analysis of the cecal transcriptome. Bars represent the geometric means. **p* < 0.05; ***p* < 0.01; ****p* < 0.001.

CellPress
OPEN ACCESS

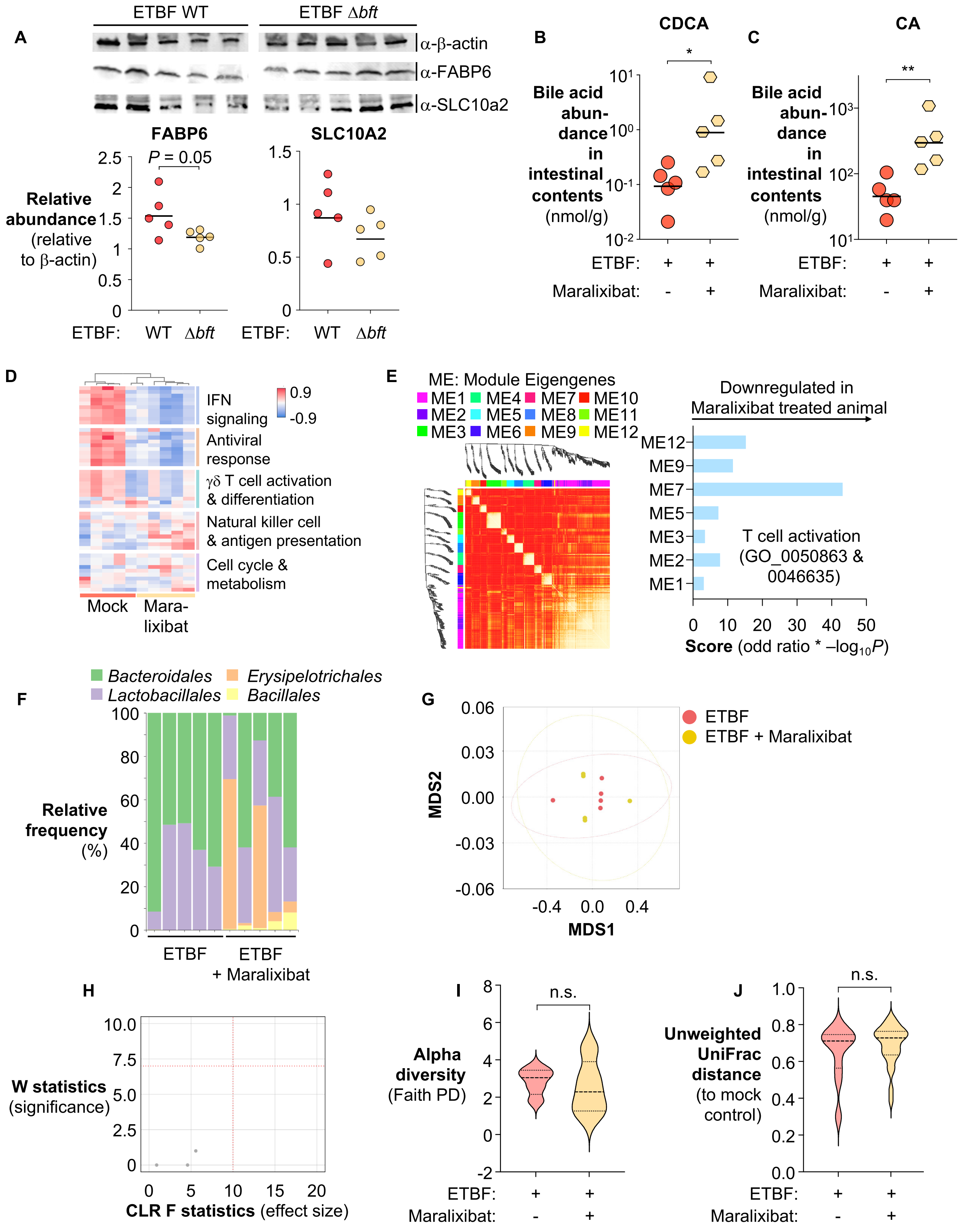

(legend on next page)

Figure S11. ETBF exploits the bile acid recycling pathway to promote bile acid depletion and gut inflammation, related to Figure 7

(A) C57BL/6 mice were colonized with either WT ETBF or the isogenic Δ*bft* mutant for 7 days. SLC10A2 and FABP6 protein levels in the colonic tissue were assessed using specific antibodies. Relative abundance was quantified in the panel below.

(B–J) C57BL/6 mice colonized with ETBF were treated with either a maralixibat-fortified diet or a control diet for 7 days. (B and C) Bile acid levels in intestinal contents measured by LC-MS/MS: (B) chenodeoxycholic acid (CDCA) and (C) cholic acid (CA). (D and E) Intestinal RNA was extracted and transcriptome profiled by RNA-seq. (D) Heatmap of differentially expressed genes and enriched signaling pathways. (E) Weighted gene co-expression network analysis (WGCNA) of the cecal transcriptome. (F–J) Gut microbiota composition was analyzed by 16S rDNA sequencing of intestinal contents. (F) Order-level microbiota composition. (G) Multidimensional scaling (MDS) analysis of microbiota composition. (H) Volcano plot from analysis of composition of microbiomes (ANCOM), with dotted lines indicating minimal cutoffs for differential abundance. (I) Violin plot showing alpha diversity (Faith's phylogenetic diversity). (J) Violin plot showing beta diversity (unweighted UniFrac distance). Bars represent the geometric means. n.s., not significant; *$p < 0.05$; **$p < 0.01$. For (I) and (J), the central thick dotted line indicates the median, while the upper and lower dotted lines correspond to the first and third quartiles (the 25th and 75th percentiles) of the data.

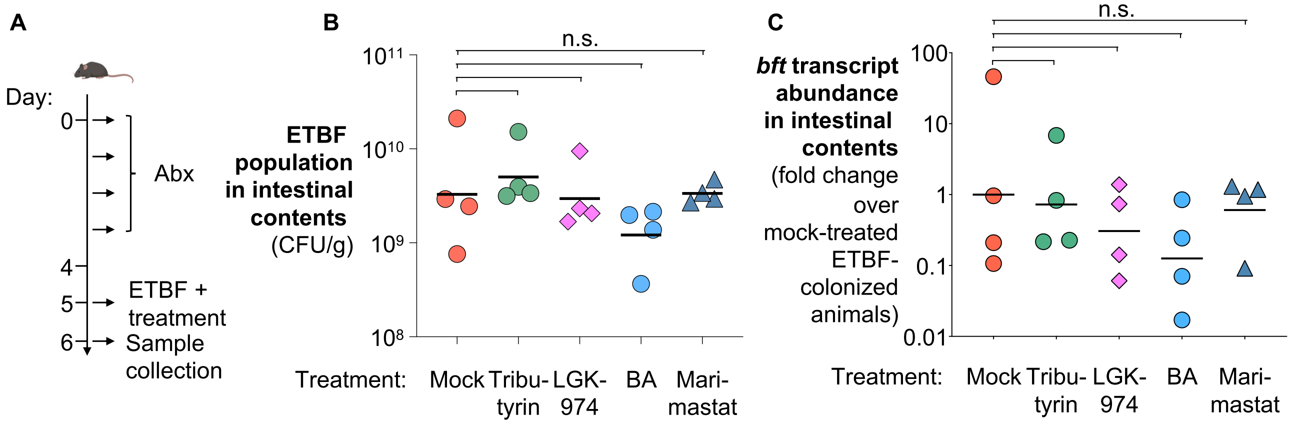

Figure S12. ETBF expresses comparable levels of *bft* in mice receiving host-targeting treatments, related to Figures 3, 4, 5, 6, and 7

(A–C) Groups of antibiotic-pretreated C57BL/6 mice were colonized with the indicated ETBF strains and treated with the indicated host-targeting interventions by intraperitoneal injection. Intestinal contents were collected at 1 day post infection. (A) Experimental schematic. (B) ETBF abundance in intestinal contents quantified by selective plating. (C) *bft* transcript abundance measured by real-time quantitative PCR. To ensure specificity of *bft* detection in the context of a complex microbiota, *bft* expression was normalized to the metalloprotease gene *mp2*. Bars represent the geometric mean. n.s., not significant.

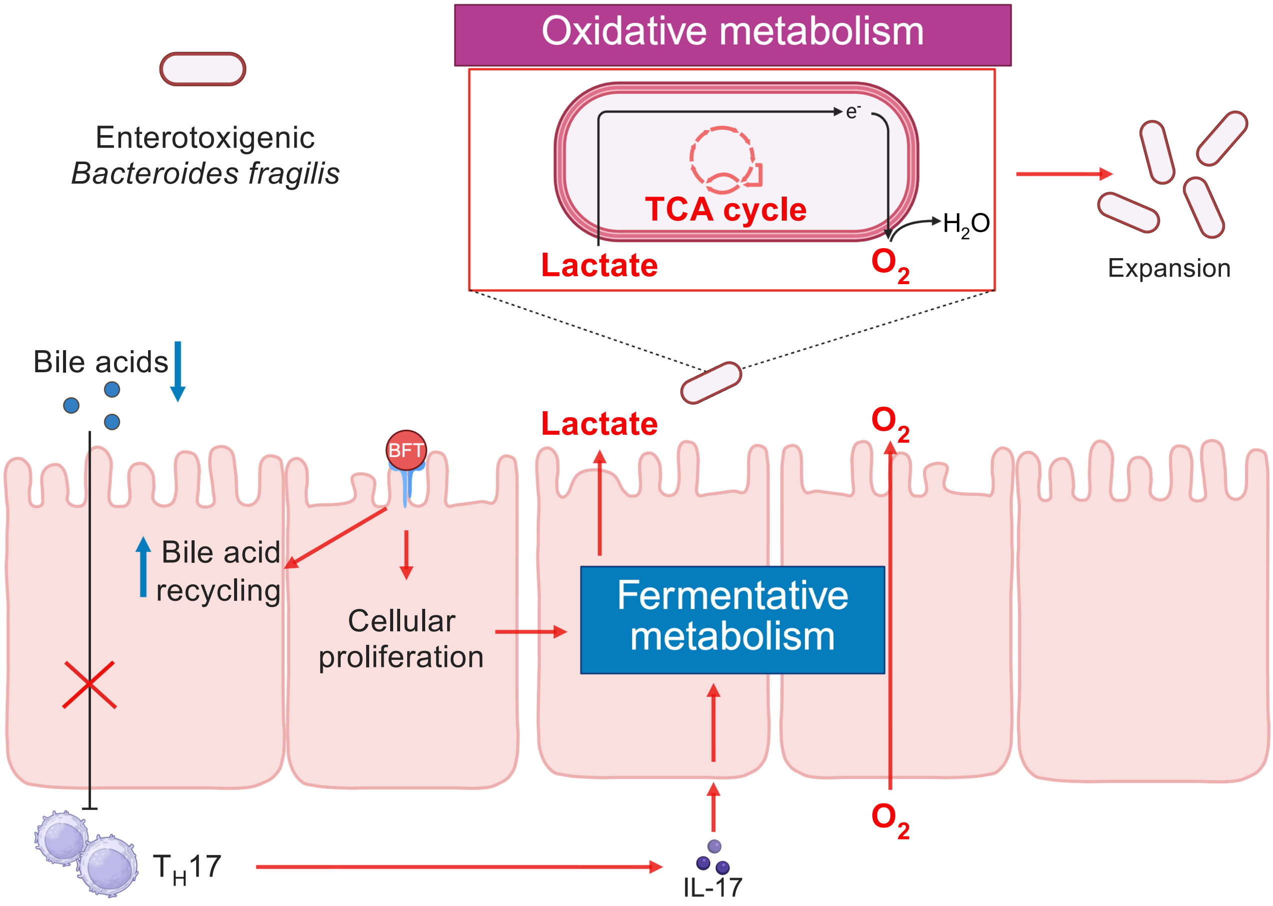

Figure S13. Proposed model for ETBF colonization of the inflamed gut, related to Figures 1, 2, 3, 4, 5, 6, and 7

We propose that BFT induces a metabolic shift in colonocytes from oxidative phosphorylation to fermentative metabolism, resulting in increased O_2 and lactate levels in the gut. These alterations create an oxidative environment within the otherwise anaerobic gut, enabling ETBF, an anaerobe, to operate an oxidative metabolism that supports colonization of the inflamed gut.
